# A Structure-Based Approach for Detection of Thiol Oxidoreductases and Their Catalytic Redox-Active Cysteine Residues

**DOI:** 10.1371/journal.pcbi.1000383

**Published:** 2009-05-08

**Authors:** Stefano M. Marino, Vadim N. Gladyshev

**Affiliations:** Department of Biochemistry and Redox Biology Center, University of Nebraska, Lincoln, Nebraska, United States of America; Wake Forest University, United States of America

## Abstract

Cysteine (Cys) residues often play critical roles in proteins, for example, in the formation of structural disulfide bonds, metal binding, targeting proteins to the membranes, and various catalytic functions. However, the structural determinants for various Cys functions are not clear. Thiol oxidoreductases, which are enzymes containing catalytic redox-active Cys residues, have been extensively studied, but even for these proteins there is little understanding of what distinguishes their catalytic redox Cys from other Cys functions. Herein, we characterized thiol oxidoreductases at a structural level and developed an algorithm that can recognize these enzymes by (i) analyzing amino acid and secondary structure composition of the active site and its similarity to known active sites containing redox Cys and (ii) calculating accessibility, active site location, and reactivity of Cys. For proteins with known or modeled structures, this method can identify proteins with catalytic Cys residues and distinguish thiol oxidoreductases from the enzymes containing other catalytic Cys types. Furthermore, by applying this procedure to *Saccharomyces cerevisiae* proteins containing conserved Cys, we could identify the majority of known yeast thiol oxidoreductases. This study provides insights into the structural properties of catalytic redox-active Cys and should further help to recognize thiol oxidoreductases in protein sequence and structure databases.

## Author Summary

Among the 20 amino acids commonly found in proteins, cysteine (Cys) is special in that it is present more often than other residues in functionally important locations within proteins. Some of these functions include metal binding, catalysis, structural stability, and posttranslational modifications. Identifying these functions in proteins of unknown function is difficult, in part because it is unclear which features distinguish one Cys function from the other. Among proteins with functionally important Cys, a large group of proteins utilizes this residue for redox catalysis. These proteins possess different folds and are collectively known as thiol oxidoreductases. In this work, we developed a procedure that allows recognition of these proteins by analyzing their structures or structural models. The method is based on the analyses of amino acid and secondary structure composition of Cys environment in proteins, their similarity to known thiol oxidoreductases, and calculations of Cys accessibility, reactivity, and location in predicted active sites. The procedure performed well on a set of test proteins and also selectively recognized thiol oxidoreductases by analyzing the *Saccharomyces cerevisiae* protein set. Thus, this study generated new information about the structural features of thiol oxidoreductases and may help to recognize these proteins in protein structure databases.

## Introduction

Compared to other amino acids in proteins, cysteine (Cys) residues are less frequent, yet often more conserved and found in functionally important locations. Protein-based Cys thiols can be divided into several broad categories wherein these residues (i) are engaged in structural disulfide bonds, (ii) coordinate metals, (iii) carry out catalysis, (iv) serve as sites of various posttranslational modification, or (v) are simply dispensable for protein function.

Structural disulfide bonds are typically observed in oxidizing environments such as periplasm in prokaryotes, and extracellular space and the endoplasmic reticulum (ER) in eukaryotes. Structural disulfides are formed by designated systems for oxidative protein folding, for example DsbA and DsbB in bacteria and protein disulfide isomerase and Ero1 in the eukaryotic ER. In addition, disulfides as stabilizing or regulatory elements may occur intracellularly. However, there are also situations when the introduced intraprotein disulfide leads to a decreased protein stability [1]. Structural stability may also be achieved when Cys residues are linked by metal ions, such as zinc and iron. In addition, Cys-coordinated metal ions may serve catalytic functions; for example, when the metal is zinc, copper, nickel, molybdenum or iron. Metal-coordinating thiols are typically found intracellularly [2],[3], but may also occur in the extracellular space.

Another important functional category of Cys residues involves catalytic Cys that act as nucleophiles. This situation occurs, for example, in Cys proteases and tyrosine phosphatases where Cys does not change redox state during catalysis, and in thioredoxins and glutaredoxins where Cys undergoes reversible oxidation and reduction. In the latter case, thiol oxidation may result in the formation of an intermediate disulfide bond with another protein thiol. In the absence of nearby Cys residue, thiol oxidation may lead to sulfenic acid (-SOH), sulfinic acid (-SO_2_H), S-nitrosothiol (-SNO), or S-glutathionylation (-SSG). In the majority of these intermediates (with the exception of sulfinic acids), the oxidized forms of Cys can be reduced by thiol oxidoreductases, such as thioredoxin and glutaredoxin, by glutathione, or by other protein and low molecular weight reductants [4],[5]. Even sulfinic acids can be reduced in a select class of proteins, for example, in peroxiredoxins by a protein known as sulfiredoxin [6]. Since these oxidized thiol forms are often reversible, they constitute a facile switch for modulating protein activity and function.

Reversible thiol oxidation has received considerable attention in recent years due to its ability to regulate proteins, protect them against stress and influence signaling. For example, sulfenic acid formation is often an intermediate step in generating disulfides [7]. Recent work has analyzed Cys-SOH formation in a set of test proteins by examining their functional sites and electrostatic properties [8]. The authors characterized several features of these proteins including significant underrepresentation of charged residues and occurrence of polar uncharged residues in the vicinity of modified Cys. Nevertheless, at present little is known about the sequence or structural features that can be employed to predict these proteins in sequence or structure databases. Much recent work has focused on S-glutathionylation [9], but common features of these modification sites are also unclear, especially as tools to identify other glutathionylation sites. Similarly, the determinants of S-nitrosylation are poorly understood. In the latter case, previously reported features include the presence of acid-base motifs flanking the modified Cys [10], and, in contrast to the Cys-SOH-containing proteins, higher frequency of charged residues.

In addition, attempts have been made to examine sites of Cys oxidation at a structural level. One study evaluated simple structural properties and aimed at identifying common features of the environment in the vicinity of Cys residues that undergo reversible redox changes [11]. Parameters that positively correlated with the occurrence of these Cys included (i) proximity to another Cys residue; (ii) low pKa (lower than ca. 9.06); and (iii) significant exposure (greater than 1.3 Å^2^) of the sulfur atom to solvent. Additional parameters reported were spatial proximity of both proton donor and proton acceptor to the redox Cys. However, this generic approach combined the analysis of catalytic and regulatory Cys, which by nature, are different. In addition, with this approach, almost all protein tyrosine phosphatases, ubiquitin-activating E1-like enzymes, thymidylate synthases and other enzymes with catalytic non-redox Cys could be detected, mainly because of their reactive (i.e., low pKa and high exposure) catalytic Cys.

Although Cys residues often serve roles critical to protein function and regulation, the presence of a Cys *per se* by no means implies any of these features. Analyses of Cys conservation may help identify some catalytic and functional Cys, but mostly for proteins with already known functions. Nevertheless, at present, sequence-based methods provide the most straightforward approach to analyze Cys function. For example, many catalytic redox Cys can be efficiently identified by searching for Cys-selenocysteine (Sec) pairs in homologous sequences [12]. This idea stems from the observations that known functions of Sec are limited to redox functions and that most selenoproteins have homologs in which Sec is replaced with a conserved Cys (implicating this Cys in redox catalysis).

We hypothesized that identification of Cys function may be assisted by examining unique features of each Cys function in proteins. In this work, we analyzed general features of catalytic redox-active Cys via functional profiles of active sites and structural analyses of reaction centers. When integrated with the tools for enzyme active site prediction and titration properties of active site residues, this approach allowed efficient prediction of thiol oxidoreductases in protein structure databases.

## Results/Discussion

### Reference Datasets

To examine common features of thiol oxidoreductase active sites, we first built a protein dataset containing previously described thiol oxidoreductases. It included representative members of protein families with known three-dimensional structures. We paid particular attention to balance the representation of thioredoxin fold (which is the most common fold found in thiol oxidoreductases) and non-thioredoxin fold oxidoreductases.

The resulting dataset consisted of 75 structures in which none of the protein domains, as defined by SCOP classification, was represented by more than 7 structures. Of these 75 proteins, 40 had thioredoxin-fold, including homologs of glutathione peroxidase (10 representatives), thioredoxin (7), glutaredoxin/thioltransferase (13), protein disulfide isomerase (3), DsbA (2), C-terminal domain of DsbC/DsbG (2), selenoprotein W (2) and ArsC (1). The non-thioredoxin fold proteins of our redox dataset included 35 proteins organized in 10 structural folds (thirteen protein families), including FAD/NAD-dependent reductase (9 representatives), Ohr/OsmC resistance protein (6), methionine-S-sulfoxide reductase (3), reductase with the protein tyrosine phosphatase fold (3), GAF-domain methionine sulfoxide reductase fRMsr (2), FAD-dependent thiol oxidase (2), methionine-R-sulfoxide reductase (2), antioxidant defense protein AhpD (2), Ero1 (1), and thiol-disulfide interchange protein DsbD (1). The complete dataset is shown in Table S1.

Our initial analyses suggested that the most challenging problem in characterizing the general features of redox-active Cys is distinguishing them from other catalytic Cys residues. Clearly, these two Cys types share active-site location and high reactivity (e.g., both redox and non-redox Cys are often strong nucleophiles). To ascertain differences between these protein classes, we built a separate control dataset of proteins containing catalytic non-redox Cys (Table S2). This set was composed of 36 proteins (organized in the form of 17 families/9 folds) including papain-like (9 representatives), penta-EF-hand (2), ubiquitin carboxyl-terminal hydrolase UCH-13 (1), FMDV leader protease (1), caspase catalytic domain (3), gingipain R (1), adenain-like (2), pyrrolidone carboxyl peptidase (1), hedgehog C-terminal autoprocessing domain (1), high molecular weight phosphotyrosine protein phosphatase (4), dual specificity phosphatase-like (2), thymidylase synthase/dCMP hydroxymethylase (2), low molecular weight phosphotyrosine protein phosphatase (1), calpain large subunit, catalytic domain (domain II) (1), dipeptidyl peptidase I (cathepsin C) domain (1), viral Cys protease of trypsin fold (2), Ulp1 protease family (1), and ubiquitin-activating enzyme (1).

**Figure 1:**
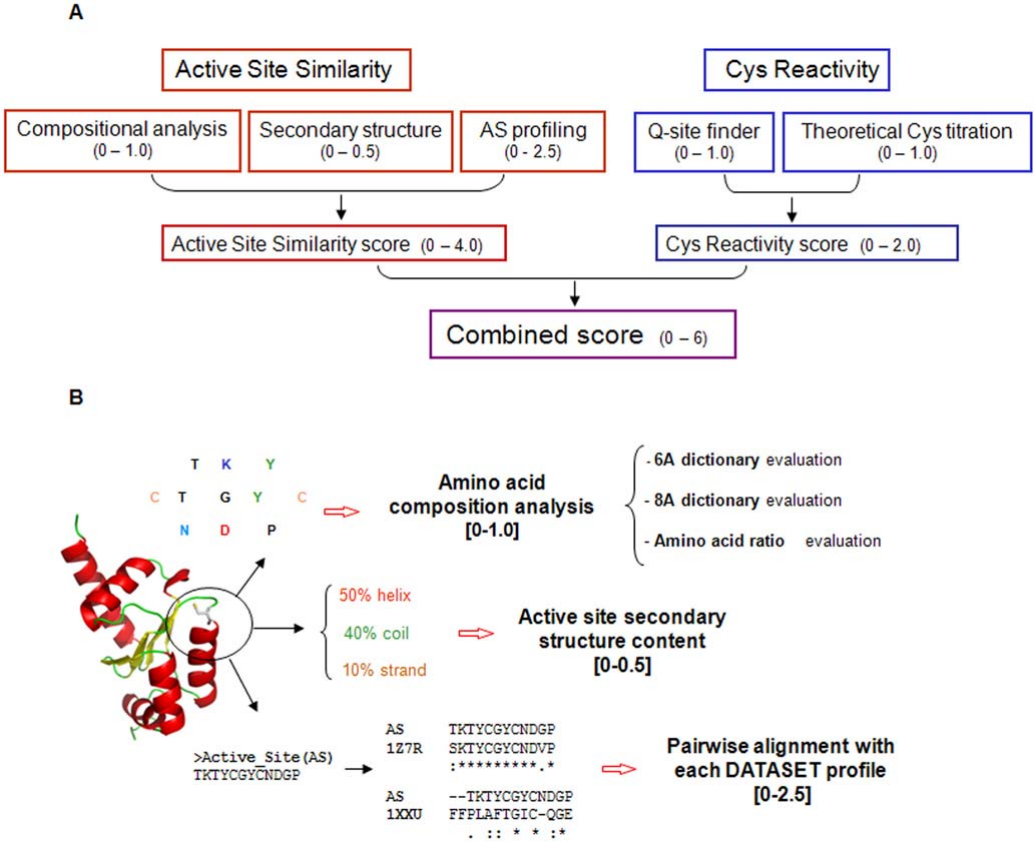
A method for prediction of thiol oxidoreductases in structure databases. (A) Overview of the complete method for prediction of thiol oxidoreductases. Red rectangles correspond to the Active Site Similarity portion of the method, and blue rectangles to the Cys Reactivity portion, each composed of several steps as discussed in the text. Each step was carried out independently converging into the final scoring function (SF) that ranged from 0 to 6.0. For each of step, the range of values in the final SF is reported in brackets, reflecting the different weights of the components. (B) The Active Site Similarity method. This part of the complete procedure is illustrated with an example of a Cys (represented in sticks)-containing protein. The active site (AS) around the Cys is analyzed in the following independent steps: (i) amino acid composition; (ii) secondary structure content; (iii) 3D structural profile analysis. For each of the three steps, the range of values in the final scoring function (SF) is reported in brackets. The amino acid composition step is further subdivided in three subparts, as detailed in the text.

The method further presented in this work is divided into two parts (Figure 1A): the first employs knowledge-based information for detection of thiol oxidoreductases by analyzing structural and compositional similarity to the active sites of known thiol oxidoreductases; and the second makes use of energy-based methods to assess properties of the catalytic redox-active Cys. For simplicity we refer to the first part as Active Site Similarity, and to the second as Cys Reactivity.

### Active Site Similarity: Compositional Analysis

The Active Site Similarity analysis included three independent steps: (i) amino acid composition of active sites at two distances from the catalytic Cys; (ii) structural profiles of active sites; and (iii) secondary structure profiles. Each of these steps contributed to the scoring function (SF).

To analyze amino acid composition of the region surrounding catalytic Cys in known thiol oxidoreductases, we determined the occurrence of amino acids within a sphere centered at the sulfur atom of the catalytic Cys with two radii, 6 Å and 8 Å (Figure 1B). For this, we separately examined thioredoxin-fold, FAD-containing, and other non-thioredoxin fold thiol oxidoreductases.

**Figure 2:**
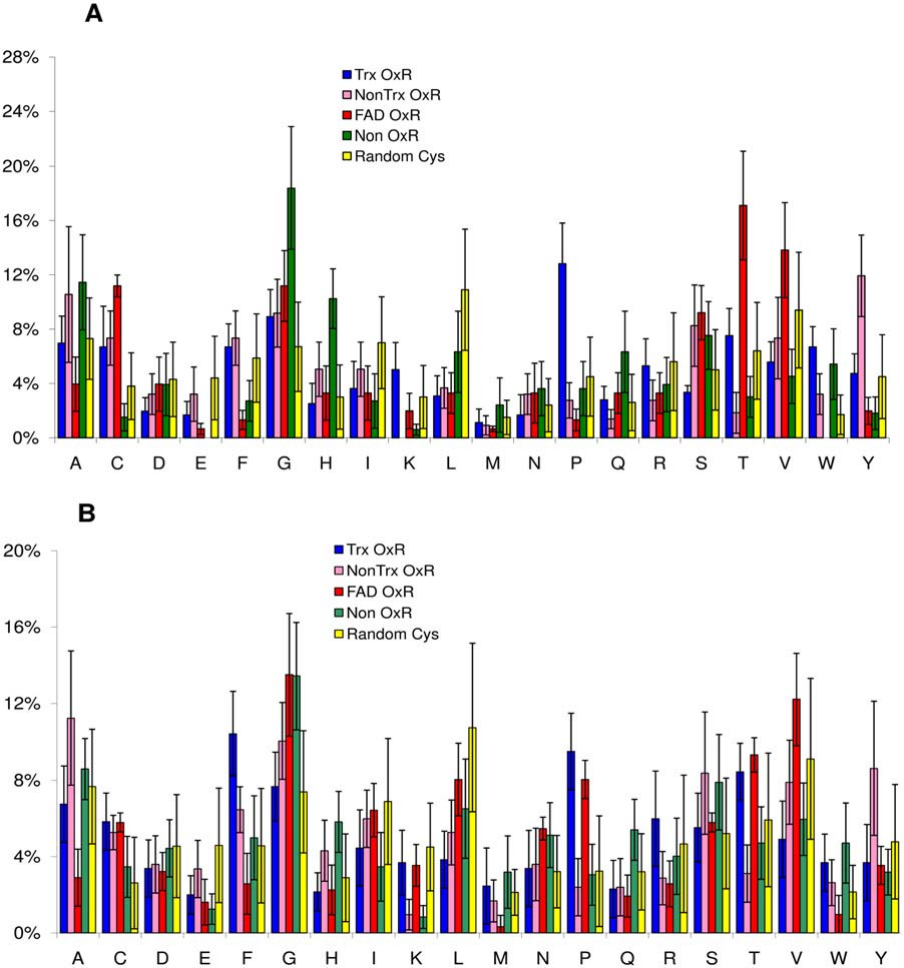
Amino acid composition of thiol oxidoreductases and control proteins. An average composition at 6Å (A) and 8 Å (B) distances from Cys for proteins in the thiol oxidoreductase and control datasets, separated by function and fold as described in the text. Abbreviations: Trx fold OxR, thioredoxin-fold thiol oxidoreductases; Non Trx fold OxR, other thiol oxidoreductases; FAD OxR, FAD-containing thiol oxidoreductases and Non OxR, proteins in the control dataset (Table S2). Random Cys refers to a set of 1800 proteins randomly selected from PDB, mentioned in the text. The calculated standard deviations are also shown.

For comparison, we analyzed two sets of randomly chosen Cys-containing proteins (800 and 1000 proteins, respectively), from which any proteins present in the thiol oxidoreductase and control datasets were excluded. Cys residues present in randomly chosen proteins represented an average composition of amino acids in the vicinity of Cys in protein structures. For each of the six so-defined groups of proteins (i.e., three groups of thiol oxidoreductases, a group containing catalytic non-redox Cys, and two groups of randomly chosen proteins) an average amino acid composition was calculated for 6 Å and 8 Å distances from the sulfur atom of Cys (Figure 2). Interestingly, each group of proteins with catalytic Cys showed unique amino acid occurrence that was also different from those of the two sets of randomly chosen proteins. This was particularly evident in the 6 Å datasets.

**Figure 3:**
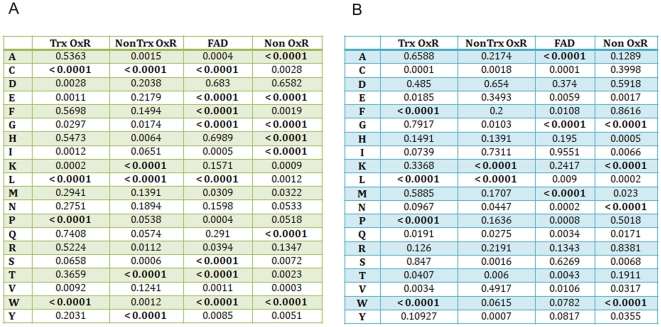
Calculated p-values for average occurrence of amino acids. (A) p-values for occurrence of each amino acid within 6 Å of the catalytic Cys (Figure 2A) for different types of proteins in our dataset. Values were obtained using t-test (comparing the whole population of each type with the whole population of the Random Cys set) using GraphPad. (B) p-values for occurrence of each amino acid within 8 Å of the catalytic Cys (Figure 2B) for different types of proteins in our dataset. Residues with most significant p-values are highlighted in bold.

However, statistical analysis of these data (standard deviations are given in Figure 2 and the p-values for frequency counts are listed in Figure 3) showed that some differences observed were not significant. Thus, a complete definition of thiol-oxidoreductases based only on amino acid frequency is not possible. Nevertheless, these data can be used, in a multi-parameter approach like the one presented here, to contribute to the description and predictability of these enzymes. Thus, we proceeded in our analyses considering the average values as shown in Figure 2.

We further employed the average occurrences of each amino acid in the vicinity of Cys as profiles (or dictionaries, to avoid confusion with the structural profiles described later on), specific for each set, in which every amino acid had its protein function-specific occurrence. The use of these dictionaries as predictive tool is straightforward: for a given protein, occurrences of amino acids located within 6 Å and separately within 8 Å of each Cys sulfur atom are calculated, compared with the dictionaries of each reference protein class, and scored.

The occurrence that receives the highest score is assigned to the corresponding protein class. For example, when a score is closest to those of thiol oxidoreductase dictionaries, it is considered positive, and in all other cases it is considered negative. In the former case, a positive value (0.375 from each of the 6 Å and 8 Å distance calculations) is given to the final SF while in other cases a null value is given. Thus, the dictionary component of the Compositional analysis can give an overall contribution of up to 0.75 to the SF.

These analyses detected differences in amino acid occurrence around catalytic Cys between thiol oxidoreductases and proteins containing catalytic non-redox Cys residues. In addition, within thiol oxidoreductases, the amino acid composition of FAD-containing enzymes was unique. For example, thioredoxin-fold thiol oxidoreductases showed an overall high representation of aromatic residues near the catalytic Cys, whereas FAD-containing thiol oxidoreductases showed lower occurrences of these residues. Thus, for this step of the procedure, FAD-containing thiol oxidoreductases were not considered. It should be noted that this did not affect the overall analysis as other steps of the method performed well with these enzymes and they could still be identified by the overall method. With this restriction, we found that several amino acids, including Pro, Cys, Trp, Tyr and Phe, were overrepresented in thiol oxidoreductases (Figure 2). At the same time, Met, His, Gly, and Glu were found to be less frequent in these proteins.

Based on this information, we empirically defined the following formula that allowed separation of thiol oxidoreductases and other Cys-containing proteins: (W+Y+F+1.5C+0.5P)/(G+H+Q+2M), where letters correspond to abundances of amino acids (in single letter code) and the numbers are coefficients. In developing this formula, we sampled different coefficients and applied the formulae to true positive and control (S1 and S2) datasets. The coefficients most efficiently separating thiol oxidoreductases from other proteins were kept.

The ratio in the formula reflected common features of thiol oxidoreductases, distinguishing them from enzymes containing non-redox catalytic Cys. For example, active sites of thiol oxidoreductases preferred non-polar aromatic residues. While all aromatic amino acids were overrepresented (compared to their average values in control sets, see Random Cys in Figure 2), histidine was less frequent (but it had high frequency in non-redox proteins with catalytic Cys). Consequently, all aromatic residues appeared in the numerator of the formula, but histidine was placed in the denominator. Other features of catalytic Cys were also included in the formula such as the well known preference for a second Cys (often a resolving Cys) in the proximity of the catalytic Cys, while the enzymes containing non-redox catalytic Cys showed a significant underrepresentation of additional Cys in the active sites. Proline is also often observed in thiol oxidoreductases, but is less frequently found in other enzymes (Figure 2).

Although the chemical basis for differences in the use of amino acid in the vicinity of Cys is not fully clear, the application of this formula was found to be quite effective. Generally, values higher than 1.0 corresponded to thiol oxidoreductases. For example, 79% thiol oxidoreductases (Table S1) had scores higher than 1.0, whereas in the control dataset (Table S2), 88% proteins had a score lower than 1.0.

When representatives of the Random Cys sets were screened with the formula, the ratio of false positive prediction (i.e., non thiol oxidoreductases scoring higher than 1.0) somewhat increased, e.g., among 100 analyzed proteins from the Random Cys set 1, 22% scored above 1.0. Interestingly, many of these scoring proteins contained metal-binding Cys. This was mainly because Cys residues clustered in these proteins (e.g., in zinc finger or iron-sulfur cluster-containing proteins). Thus, the contribution to the SF from this last component of the Compositional analysis was lower than that of the dictionaries, adding a value of up to 0.25 to the SF. Finally, when the three components of the Compositional analysis (analysis of dictionaries for 6 Å and 8 Å and the application of the formula) were considered, the contribution to the SF ranged from 0 to 1.0 (Figure 1).

### Active Site Similarity: Structural Profile Analysis

A previous study assessed structural similarity of reaction centers by profiling functional sites in proteins [13]. It built a signature sequence of amino acids located in the active sites. In our work, segments of amino acids in the active sites were extracted from the structure and combined into a single contiguous sequence (called either structural profile or active site signature). A similar approach was recently employed to examine proteins with Cys oxidized to sulfenic acid [8], in which active sites were defined as an area located within 10 Å from the oxidized Cys. This study [8] proposed that pairwise alignments between signatures can be effective in predicting protein function by analyzing an unknown profile against a set of known profiles.

We used this idea and employed the 8 Å active site signatures derived from each thiol oxidoreductase in our dataset (Table S1) as the set of known profiles. It should be noted that, compared to the original procedure [13], the parameters for weighting pairwise alignments (i.e., relative weights for similarities, gaps and identities) were empirically optimized to achieve the best separation of thiol oxidoreductases and reference datasets (Figures S1 and S2). The optimized parameters for equation 1 are described in detail in the Methods section.

The ability of this procedure to separate thiol oxidoreductases from other proteins is remarkable; using an appropriate cut off for the output of equation 1 (for example, 0.4 in Figure S1) as described in the Methods section, no false positives were detected. This feature (i.e., very low false positive rate) opened up an opportunity, based on the structural profile analysis, to assign a wider range of values as contributing to the SF. In particular, values higher than 1.0 could be given to the SF when the output of equation 1 is sufficiently high. However, values higher than 1.0 were appended to the SF only under the conditions where the probability of false positive predictions was either null or very low. The contribution of this procedure to the SF ranged from 0 to 2.5 with the latter occurring only when the profile of a putative protein under examination was almost identical to that of a known thiol oxidoreductase. Further details on this part of the procedure are given in the Methods section.

### Active Site Similarity: Secondary Structure

We analyzed secondary structure composition within 6 Å from the catalytic Cys for all proteins in our datasets (Figure S3). A marked preference for alpha helical and loop geometries around the Cys was found in thiol oxidoreductases. In turn, beta strands were infrequent (with notable exception of MsrBs).

We implemented these observations with a simple function requiring helical composition exceeding 35% and loops exceeding the composition of strands. As alluded above, some thiol oxidoreductases (MsrBs, fRMsrs and arsenate reductases) were missed at this step of the analysis. Since this procedure could potentially miss other candidate thiol oxidoreductases, its contribution to the SF ranged from 0 to 0.5.

When the three steps of the procedure (i.e., amino acid composition, structural profile and secondary structure composition of the active sites) were applied together to thiol oxidoreductase and control datasets, a nearly complete separation of thiol oxidoreductases and other proteins was achieved (Figure S4). Each thiol oxidoreductase (Table S1) received scores higher (≥1.5) than any control protein (Table S2 and a representative subset of the randomly chosen proteins), with a single exception: a low molecular weight tyrosine phosphatase (PDB code 1D1P) scored as high as some of the low scoring thiol oxidoreductases. However, this phosphatase showed marked analogy to thiol oxidoreductases (e.g., some proteins annotated as low molecular weight tyrosine phosphatases are in fact arsenate reductases). We discuss this feature in greater detail later in the text (see results of the Yeast Analysis).

### Cys Reactivity

We hypothesized that properties of redox-active catalytic Cys could also be suitable for distinguishing thiol oxidoreductases from proteins with other Cys types. In addition, proteins with catalytic Cys could potentially be distinguished from those with non-catalytic Cys by virtue of thiol oxidoreductases being enzymes. Thus, we examined available active site prediction programs with respect to recognition of Cys active sites in thiol oxidoreductases. These programs included Q-site finder (http://www.modelling.leeds.ac.uk/qsitefinder/), Pocket finder (http://www.modelling.leeds.ac.uk/pocketfinder/), THEMATICS (http://pfweb.chem.neu.edu/thematics/submit.html), SARIG (http://bioinfo2.weizmann.ac.il/˜pietro/SARIG/V3/index.html) and FOD (http://bioinformatics.cm-uj.krakow.pl/activesite/). All of these programs are freely accessible via web service, but some calculations could be slow (e.g., THEMATICS).

**Figure 4:**
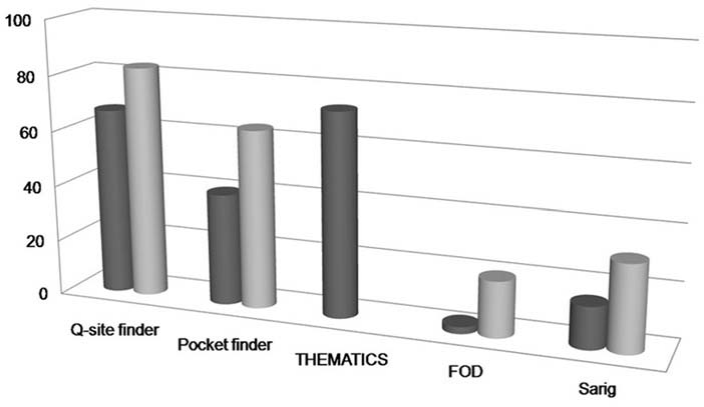
Application of methods for active site recognition to thiol oxidoreductases. Benchmarking of five publicly available programs for prediction of active site residues: Q-site finder, Pocket finder, THEMATICS, FOD and SARIG. For Q-site finder and Pocket finder, the percentage of correctly predicted proteins is shown wherein only the first three ranked sites (dark grey cylinder) or all 10 sites (light grey cylinder) are considered. For FOD, the dark grey bar shows a true positive rate using the standard cutoff, while the light grey bar represents a true positive rate when a more permissive cutoff is employed (i.e., active site residues have a normalized ΔH score>0.5). For SARIG, the dark grey bar bar represents a true positive rate with the standard cutoff, while the light grey bar shows a true positive rate when a more permissive cutoff values (closeness Z-score>0.75 and 3 Å^2^<RSA<200 Å^2^) are employed.

For each program, we examined randomly chosen 15 thioredoxin fold and 15 non-thioredoxin fold thiol oxidoreductases (Figure 4). Two programs, FOD and SARIG, were ineffective in predicting catalytic sites of thiol oxidoreductases. Pocket Finder performed slightly better but still clearly missed many active sites with catalytic redox-active Cys. The best methods for thiol oxidoreductase prediction proved to be Q-site finder and THEMATICS. The use of THEMATICS is limited by its speed. Thus, Q-site finder was further employed. Scoring of this method is detailed in the Methods section. Briefly, if a catalytic Cys ranked within the first 3 sites, a positive value (1.0) was given to the SF, and a zero value was given if the sulfur atom of Cys was not predicted in any of the 10 ranked sites. Intermediate situations resulted in the contributions to the SF, declined in the range between 0 and 1, as detailed in the Methods section.

The final step of our algorithm examined Cys titration curves. As discussed in the Introduction, pKa and exposure have recently been proposed as parameters that distinguish redox-regulated Cys from other Cys types [11]. However, when applied to our dataset, they proved to be ineffective in detecting differences between redox and non-redox catalytic Cys residues (Figure S5). This is indeed not surprising, as sulfur exposure and a reasonably low Cys pKa should be necessary features for both thiol oxidoreductases and enzymes with other nucleophilic catalytic Cys.

Thus, we examined other properties and methods that could account for accessibility and reactivity of catalytic redox Cys. While Q-site finder may possibly account for effective accessibility of Cys to small molecular probes [14], it provides no information on Cys chemistry. An alternative was to directly employ theoretical titration curves of active site Cys residues. Indeed, the main idea of THEMATICS is based on the observation that theoretical titration curves and their deviation from standard Henderson-Hasselbach (HH) behavior can inform on the location of active site residues (if they are titrable). Analysis of the theoretical titration curve of a titrable residue is often more informative than simple calculations of its pKa [15]–[18].

**Figure 5:**
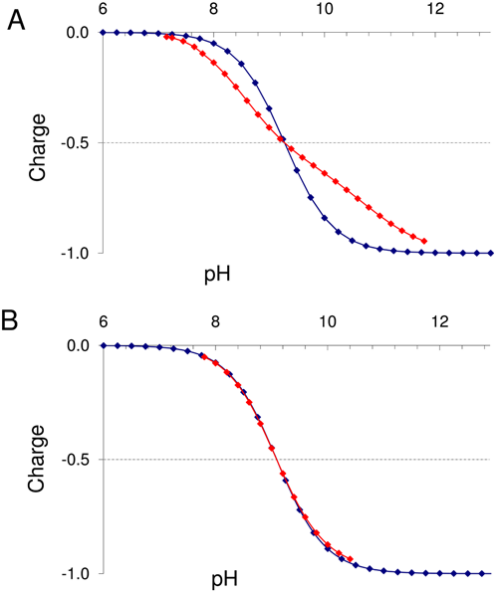
Theoretical titration curves. The calculated curves are shown in red and the corresponding standard Henderson-Hasselbach (HH) titration curves in blue. (A) An example of a highly deviating theoretical titration. (B) An example of no deviation (this is the most common situation since most Cys behave in this manner).

In this work, we employed the web accessible H++ server [19] to calculate Cys titration curves and developed in-house tools for analyzing the output (details are in the Methods section). Briefly, we examined theoretical titration curves of each candidate Cys and compared them with the standard HH behavior. The two curves were superimposed and numerically compared (Figure 5). Greater deviation between the two curves (Figure 5A) implied a higher probability of the Cys being part of the active site and was given a positive contribution (up to 1.0) to the SF, whereas small deviation or no deviation (Figure 5B) was given a zero contribution. We combined the methods discussed above in a single algorithm shown in Figure 1A.

### Tests of the Algorithm

For the initial test of the algorithm, we selected a set of randomly chosen proteins (Test Case) not included in the datasets used to develop the method, which consisted of 22 thiol oxidoreductases (13 thioredoxin-fold proteins, 4 FAD-binding proteins and 5 other non-thioredoxin fold enzymes), 13 proteins with catalytic non-redox Cys and 21 proteins with non-catalytic Cys known to be redox-regulated through nitrosylation or glutathionylation (Table S3). Several Test Case proteins were homology models. We deliberately included them as structural models ultimately represent application of the program to proteins with unknown structures.

**Figure 6:**
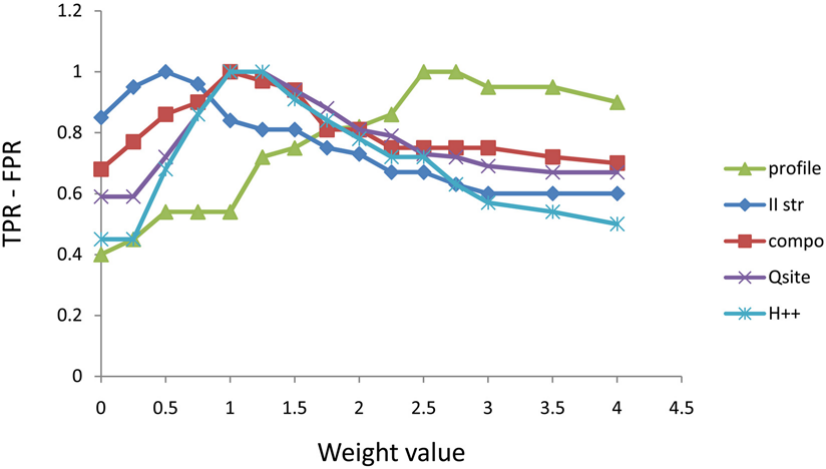
Effect of variation in parameter used on method performance. In the figure, the difference between True Positive Rate (TPR) and False Positive Rate (FPR) for each parameter weight is plotted. True positives included correctly predicted thiol oxidoreductases from the Test Case (Table S3), and false positives control proteins (Table S3) predicted as thiol oxidoreductases (i.e., containing a Cys scoring higher than the cut-off). A Y-axis value of 1 indicates complete separation of the dataset. Starting from the optimized weights described in the text, each parameter was varied separately in the range 0–4 (X-axis values). The graph should be read as follows: each parameter is represented by a curve, and each point represents the TPR-FPR value (the higher the better) for a specific weight. Maxima represent the best scoring values (compare to Figure 1A). Decreased values to the right of the maximum reflect a tendency of the parameter to give False Positive predictions when over-weighted, while when on the left, it corresponds to a tendency to underestimate the number of True Positives when the parameter is under-weighted. This analysis provides insights into variability introduced in the performance when the parameter weights are changed, in particular showing that the optimized parameters cannot vary in a broad range of values. However, for two parameters alternative values were possible: the Profile scoring (values could vary in the range 2.5-÷2.75) and the H++ contribution (1÷1.25). For all other parameters, clear maxima (corresponding to the optimized values described in the text) were observed.

The Test Case was also used to analyze weight distribution for each parameter of the algorithm; this process supported parameter weights shown in Figure 1A (values in brackets). Details of these calculations are shown in Figure S6, available as supporting information. We also assessed method performance upon changes in weights, and this is shown in Figure 6 (details are given in the figure legend).

**Figure 7:**
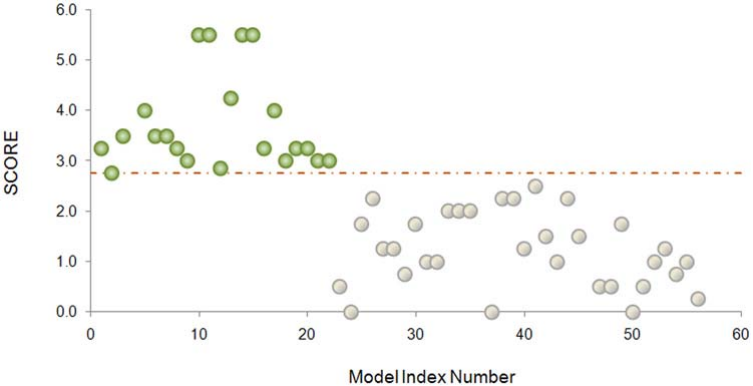
Analysis of the Test Case. Green circles show thiol oxidoreductases and grey circles other proteins of the Test Case. The Test Case is described in the text and in Table S3.

The output of the algorithm with optimized parameter weights (Figure 1A), applied to the Test Case, is shown in Figure 7. Complete separation of thiol oxidoreductases (shown by blue circles) from proteins with other Cys functions (green circles) was achieved with a cutoff value of 2.75. Details of the calculations for each protein in the Test Case are shown in Table S3.

To validate the algorithm on a genome-wide level, without any bias in the selection of proteins, we applied the method to the *Saccharomyces cerevisiae* proteome. Initially, we selected a subset of yeast proteins by including (i) all known thiol oxidoreductases found by literature search and detected by PSI-BLAST searches using known thiol oxidoreductases as queries; and (ii) all other proteins in the yeast proteome containing at least one highly conserved Cys (conserved in ≥90% homologs). From this set, proteins containing metal-binding Cys residues were filtered out using Prosite patterns. The resulting set of 292 proteins was subjected to homology modeling via Swiss Model (http://swissmodel.expasy.org/) or HOMER (http://protein.cribi.unipd.it/Homer/), which generated 149 structural models (Table S4).

**Figure 8:**
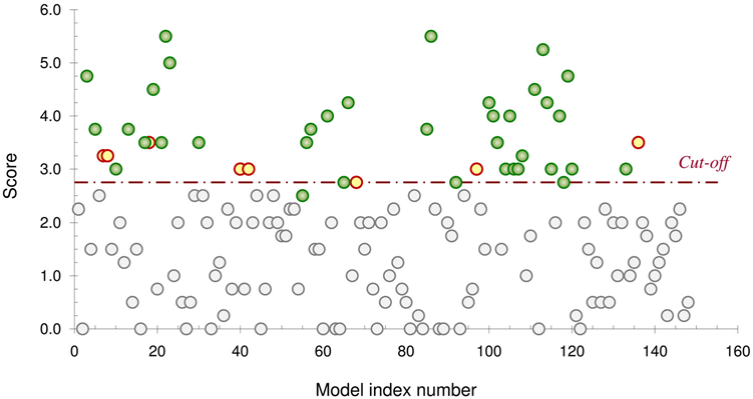
Analysis of the *Saccharomyces cerevisiae* proteome. The score for each protein of the yeast proteome (i.e., the score for the highest scoring Cys) is plotted (model index numbers follow the order in Table S5). Green circles represent thiol oxidoreductases, and yellow circles show proteins scoring with thiol oxidoreductases but not known to be redox catalysts. Gray circles represent other proteins. All but one known thiol oxidoreductase were detected.

Among these proteins, 42 were predicted by our algorithm as thiol oxidoreductases (i.e., scored≥the cutoff value of 2.75) (Figure 8 and Table S5 and Table S6). Interestingly, 33 of the 42 predicted proteins were indeed known thiol oxidoreductases, and the remaining 9 proteins likely included candidate thiol oxidoreductases and false positives. The correctly predicted thiol oxidoreductases were (Table S6) 6 glutaredoxin/glutaredoxin-like proteins (>gi|6320720, >gi|6323396, >gi|6320492, >gi|6319814 >gi|6320193, >gi|6321022), 4 thioredoxins/thioredoxin-like (>gi|6319925, >gi|6321648, >gi|6323072, >gi|6322186), 1 glutathione reductase (>gi|6325166), 2 thioredoxin reductases (>gi|6321898, >gi|6320560), 1 Ero1 (>gi|6323505), 1 Erv1 (>gi|6681846), 1 Erv2 (>gi|6325296), 5 peroxiredoxins/peroxiredoxin-like (>gi|6323613, >gi|6320661, >gi|6320661, >gi|6322180, >gi|6319407), 2 glutathione peroxidases (>gi|6322228, >gi|6322826), 1 alkyl hydroperoxidase (>gi|6323138), 1 methionine-S-sulfoxide reductase (>gi|6320881), 1 methionine-R-sulfoxide reductase (>gi|6319816), 4 protein disulfide isomerases (>gi|6319806, >gi|6324484, >gi|6324862, >gi|6320726), and 1 dihydrolipoamide dehydrogenase (>gi|14318501). The results are further illustrated in Figure 8 where all 149 yeast proteins for which models have been generated are represented (green circles correspond to known thiol oxidoreductases).

One of the candidate thiol oxidoreductases was 6-O-methylguanine-DNA methylase. Interestingly, in addition to this algorithm, this protein was predicted as thiol oxidoreductase by a method based on Cys/Sec pairs in homologous sequences [12]. As the structure of *E. coli* 6-O-methylguanine-DNA methylase is known (PDB code 1sfe), we separately subjected this protein to our algorithm. For Cys135 of this protein, the score was 3.75, a value above the cutoff. The same Cys was predicted by the Cys/Sec method. Overall, the data suggest that yeast 6-O-methylguanine-DNA methylase is a strong candidate for a novel thiol oxidoreductase.

Other predictions included (i) >gi|6325330| homologous to mammalian PTP (LTP1), (ii) >gi|6321631| glyceraldehyde-3-phosphate dehydrogenase 1(GAPDH-1); (iii) >gi|6322409| glyceraldehyde-3-phosphate dehydrogenase 2 (GAPDH-2); (iv) >gi|6324268| similar to tRNA and rRNA cytosine-C5-methylase (NOP2); (v) gi|14318558| ubiquinol-cytochrome c oxidoreductase subunit 6 (QCR6); (vi) >gi|6322155|ref|NP_012230.1| capping - addition of actin subunits (Cap2p); (vii) >gi|6321388|ref|NP_011465.1| hypothetical ORF (Ygl050wp); and (viii) >gi|6322921|ref|NP_012994.1| hydrophilic protein implied in targeting and fusion of ER to Golgi transport vesicles (BET3). While the functions of some of these proteins are not known, the first three are worth a comment. GAPDH proteins are known to have a catalytic nucleophilic Cys in the active site which is highly sensitive to redox regulation by both thiols and reactive oxygen species [20]–[22]. Oxidized GAPDHs were also found to recover full activity in the presence of thioredoxin [23] or DTT [24]. It appears that these proteins share properties with thiol oxidoreductases, and their active site Cys showed common features with catalytic redox Cys in other enzymes.

Low molecular weight protein tyrosine phosphatases (lwPTP) share the phosphotyrosine protein phosphatase I-like fold with arsenate reductase (ArsC) of gram-positive bacteria and archaea [25], which are thiol oxidoreductases. These enzymes (lwPTP and ArsC) belong to the same superfamily (phosphotyrosine protein phosphatases I). In our original dataset, there were two ArsC proteins (PDB coded 1LJL and 1Y1L), and recognition of their nucleophilic catalytic Cys as redox-active residues may reflect such similarity. With regard to other predictions, no strong evidence to support or reject them as thiol oxidoreductases was obtained, so at least some of these proteins could indeed be thiol oxidoreductases.

Finally, one known thiol oxidoreductase among the 149 modeled yeast proteins was not correctly detected by our method. This protein was a monothiol glutaredoxin (>gi|6319488, GRX7), which corresponds to the single green circle in Figure 8 located slightly below the cut off value. However, the only contribution to the score for this protein came from the Active Site Similarity method, whereas the Cys Reactivity contribution was zero: Q-site finder did not predict its catalytic Cys in any one of the 10 ranked sites, and the Cys titration curve strictly followed HH behavior. We also submitted the protein to the THEMATICS server, but its catalytic Cys was not predicted as an active site residue. The fact that these independent structure-based calculations, which proved to be quite effective in other analyses, did not recognize the active site and its catalytic Cys could potentially be explained by poor quality of the homology model.

It can be argued, that the Similarity part of our algorithm should work better than the Cys reactivity part with scarcely refined (but still reasonable) structural models, due to its lesser dependence (especially for secondary structure and compositional analysis) on the accuracy of predicted atomic positions; these, in turn, determine titration curves and all types of docking-like calculations (e.g., Q-site finder predictions). Therefore, poorly refined structural models should affect predictions of the energy-based calculations of the Cys reactivity part of the method to a greater extent. Finally, it should be noted that all other glutaredoxins and glutaredoxin-like proteins could be confidently predicted, which is consistent with the idea that the low score for Cys reactivity in GRX7 may be related to the quality of the structural model rather than inability of the procedure to detect this specific protein. Overall, the method presented in this study showed very good selectivity and specificity. It should find applications in examining protein structures and identifying new thiol oxidoreductases and catalytic redox-active Cys residues in these proteins.

During the review of our study, another paper was published [26] that analyzed performance of active site prediction and employed multiple and independent parameters. The authors observed improved performance when the analyses included theoretical titration curves, residue exposure and sequence alignment-based conservation scores. This study and our work suggest that implementing different chemical (e.g., titration curves), physical (e.g., solvent accessibility), and biological (e.g., sequence alignment) parameters offers a promising approach to develop efficient tools for protein structure-function predictions. Such approaches may allow the user to achieve specific biologically meaningful insights, a feature often missing in predictive bioinformatics tools. Finally, we suggest that the use of similar approaches may address the challenging issue of prediction of Cys-based modification sites in proteins.

## Methods

### Sequence Analysis

A set of known thiol oxidoreductases present in *Saccharomyces cerevisiae* was collected by searching literature, analyzing homology to known thiol oxidoreductases from other organisms, and similarities to Sec-containing proteins [12]. Sequence alignments were prepared with PSI-BLAST against the NCBI nonredundant protein database with the following search parameters: expectation value 1e-4, expectation value for multipass model 1e-3, and maximal number of output sequences 5,000. Cys conservation for yeast *Saccharomyces cerevisiae* proteins was determined using an in-house Perl-script by parsing the PSI-BLAST output.

### Molecular Modeling

Models were built via Swiss Model (http://swissmodel.expasy.org/) and HOMER (http://protein.cribi.unipd.it/Homer/). VegaZZ 2.2.0 molecular modeling package was used to check for missing residues, and for minimization runs (with CHARMM22 force field), fixing planarity problems, editing multiple sidechain conformations, adjustment of incorrect geometries, and residue renumbering. Most of these operations were required for successful submission to a server, such as SARIG and H++. With HOMER analyses, the selection of template for modeling was done using PDB Blast. Calculations of pKa values for dataset proteins were made with H++ server and with PropKa implementation in VegaZZ (only for calculations shown in Figure S5, for consistency with the previously published procedure [11]). Calculations of accessible surface area were performed with a standalone program, Surface 4.0, downloaded from http://www.pharmacy.umich.edu/tsodikovlab/.

### General Overview of the Method

The overall procedure was based on observations of unique properties of active sites and catalytic Cys in thiol oxidoreductases. Each parameter of the method (Figures 1) was optimized for the ability to separate thiol oxidoreductases from other proteins.

Optimization of the parameters was carried out on an empirical basis: separately for each subpart of the method we tested different parameters and calculations were then performed against the dataset. The parameters which permitted better resolution of the dataset (i.e., better separation of thiol oxidoreductases from set 1 against other reference proteins – set S2 and representatives of the Random Cys set) were kept and used in the composite procedure. A representative example is given in Supporting information, Figure S2.

To analyze the relative weight distribution for each parameter of the algorithm and how the algorithm performance depends on them, we carried out calculations of a set of proteins (Test Case, described in the Results section) not belonging to the dataset. This analysis supported the arrangements of parameter weights shown in Figure 1A (values in brackets). Details of these calculations are shown in Figure S6, Figure 6, and Figure 7. The analysis of the Test Case also allowed us to identify a cut-off value for the scoring function (described later on in this section) to efficiently discriminate thiol oxidoreductases form other proteins. The final scoring function, SF, was made up of contributions from each part of the method, as detailed further in this section. The overall method was divided into 2 parts: the first, Active Site Similarity, analyzed structural similarity of test proteins to known thiol oxidoreductases. The second, Cys Reactivity, employed external software for energy-based calculations of Cys properties. Both parts were further subdivided into subparts, as shown in the scheme of the algorithm in Figure 1, and each is further discussed separately here in the Methods section.

### Active Site Similarity: Compositional Analysis

Analysis of amino acid composition around Cys was carried out with in-house tools written in Python (v2.4). Detection of amino acids within a cutoff distance (6 Å or 8 Å) from the catalytic Cys sulfur was made considering all residues with one or more of their atoms at a distance equal or lower than the cutoff. A simple graphical representation is shown in Figure 1B. We employed this procedure for all proteins in the dataset (Table S1 and Table S2), divided into 4 categories: (i) thioredoxin fold thiol oxidoreductases (Trx OxR); (ii) non-thioredoxin fold thiol oxidoreductases (Non Trx OxR); (iii) FAD-binding thiol oxidoreductases (FAD OxR); and (iv) proteins with catalytic non-redox Cys (Non OxR). For each protein category, we computed an average amino acid composition. This is shown in graphical form in Figure 2. Frequency of amino acid occurrence was associated with each amino acid (the Y value in Figure 2). Consequently, four separate sets of amino acid compositions were built for Trx OxR (blue bars in Figure 2), Non Trx OxR (pink bars), FAD OxR (red bars), and Non OxR (green bars). We stored information for each protein category in the form of specific dictionaries (after the Python 2.4 datatype actually employed), wherein each amino acid received a value of its frequency.

In addition, two other sets of non-overlapping randomly selected proteins, one made of 800 PDB structures and the other of 1000 structures, were built. These sets were designated Random Cys set 1 and Random Cys set 2 and represented an average composition of amino acids in the vicinity of Cys in protein structures. Combined together these two sets made up the Random Cys set (bright yellow bars in Figure 2). We required that these sets have no overlap with datasets S1 and S2. Also for these two sets, two specific dictionaries were built to store the set-specific amino acidic composition. The use of the six dictionaries to carry out compositional analysis is illustrated with the following example. Given the following short structural profile, i.e., the amino acid sequence in the active site, Cys-Ala-Val-Glu, and the following dictionaries,

“Set1” {Ala (occurrence): 0.20, Cys: 0.07…Glu: 0.13…Val: 0.29}
“Set2” {Ala: 0.10, Cys: 0.12…Glu: 0.08…Val: 0.15}
“Set3” {Ala: 0.25, Cys: 0.08…Glu: 0.17…Val: 0.30}

When applying each dictionary separately to the profile, three different scores are received, each obtained by appending the average set-specific frequency value corresponding to an amino acid of the profile:

Score (profile, Set1) = 0.07 (Cys occurrence in Set1)+0.20+0.29+0.13 = 0.69
Score (profile, Set2) = 0.12+0.1+0.08+0.15 = 0.45
Score (profile, Set3) = 0.08+0.25+0.30+0.17 = 0.80

In this example, the highest score is obtained with the “Set3” dictionary. If “Set3” corresponds, for instance, to the Trx OxR dictionary, the putative sequence resembles the composition of thiol oxidoreductases. In this case, a value of +0.375 is added to the final scoring function, SF. The same happens if the best scoring dictionary is that of Non Trx OxR. If instead the best scoring dictionary is one of non-thiol oxidoreductases, then a zero contribution is given to the SF. These dictionary-based calculations were done with 6 Å and 8 Å distance profiles, thus contributing a maximum value of 0.75 to the SF (0.375 for the 6 Å profile and 0.375 with the 8 Å profile).

Another evaluation formula, limited in this case to the 6 Å distance (because this distance shows the most significant difference among proteins in the dataset, see Figure 2) was based on the following ratio (W+Y+F+1.5C+0.5P)/(G+H+Q+2M), where letters correspond to the single letter code for amino acids and numbers are coefficients. This empirical ratio was chosen as discussed in the Results session. We sampled different coefficients (0.5, 1, 1.5, 2) for the amino acid composition in this formula: in each case the same datasets (S1 and S2) were used. The coefficients most efficiently separating thiol oxidoreductases from other proteins were kept. When the formula was applied to a profile for a putative active site, the result (x) was analyzed as follows. If x≥1.5, a value of 0.25 was given to the SF. If it was between 1.5 and 1.1, a value of 0.125 was given. Otherwise a zero value was given.

### Active Site Similarity: Structural Profiles

For this step in the procedure, we followed a previously published procedure of functional site profiling [13]. Accordingly, we employed ClustalW (http://www.ebi.ac.uk/Tools/clustalw2/index.html) standalone version 2.0.3 for pairwise alignment calculations between a putative profile and each reference profile extracted from our dataset of known thiol oxidoreductases (Table S1). The evaluation function was carried out with Equation 1
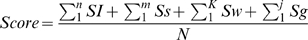
(1)where SI represents identities (n is the total number of identities in the alignment), Ss strongly conserved residues (m is the total number of Ss in the alignment), Sw weakly conserved residues (K is the total number of Sw ), Sg gaps (j is the total number of Sg) and N is the number of paired residues in the alignment.

Modified parameters were used for Equation 1 (in parenthesis are the original values, also derived empirically): SI = 1.0 (1.0), Ss = 0.3 (0.2), Sw = 0.1 (0.1), Sg = 0 (−0.5). Starting from the original parameters, we sampled different values to determine if it was possible to improve the performance of Equation 1 against our datasets (S1, S2 and representatives of the Random Cys sets). An example of the performance with modified parameters is given in Figure S2. We found that an improvement can be reached by underweighting the gaps, and we obtained the best results when the gaps were treated like “non similar” paired residues. Our parameters were more permissive than the original parameters, which were developed and optimized to address a different biological question. It must be stated that the original parameters performed better if the purpose was to detect similarities between more related protein sets (for example, functional families). For the analysis of distantly related proteins, a relaxation of parameters was necessary, and we obtained the best results with our more permissive *ad hoc* optimized parameters (Figure S2).

The flow of our structural profile analysis was as follows: given a putative active site, pairwise alignments were made with ClustalW between the putative profile and each of the profiles extracted from the known thiol oxidoreductases in our dataset (Figure 1B). Each pairwise alignment was evaluated with Equation 1. The highest scoring alignment was selected and its score value (x) was kept for further analysis. If the best result (x) of Equation 1 was lower than 0.35, a null value was given to the SF. If 0.35≤x<0.4, a value of 0.5 was given. If 0.4≤x≤0.5, a value of 1 was given. If 0.5<x≤0.6, a value of 1.5 was given. If 0.6<x≤0.75, a value of 2 was given. Finally, if x>0.75, a value of 2.5 was given to the SF. In the latter case, a x value higher than 0.75 actually meant that this profile was almost identical to that of a known thiol oxidoreductase.

### Active Site Similarity: Secondary Structure Analysis

We analyzed the secondary structure content of active sites of each thiol oxidoreductase in our dataset and then compared them with proteins in the control sets. A three-state secondary structure classification (helix, strand, or coil) was assigned to each amino acid within 6 Å from the Cys sulfur atom. The evaluation was made as following: (i) if the helical content was higher or equal to 35% and the coil content was higher than the strand content, a value of +0.5 was given to the SF. (ii) If helical content was equal to or higher than 10% and both the coil content and the helix content were higher than the strand content, a value of +0.25 was given to the SF. In all other cases, this part of the method received a zero contribution.

Thus, the overall contribution to the SF from the Active Site Similarity part of the method ranged from 0 to 4.0; once again it must be clearly stated that the latter value occurred only when a putative active site was nearly identical to that of a known thiol oxidoreductase.

### Cys Reactivity: Q-Site Finder

This part of the method was based on, but not limited to, calculations from two publicly available external servers, Q-site finder (http://www.modelling.leeds.ac.uk/qsitefinder/) and H++ (http://biophysics.cs.vt.edu/H++/index.php). We first discuss the use of Q-site finder. For an overview of this program, we refer the reader to the original paper [14]. To automate the analysis, the predictions of Q-site finder were parsed in html format with an in-house Python tool. We developed an *ad hoc* scoring of 10 differently ranked sites in the Q-site finder output, derived on an empirical basis (i.e., by testing against all dataset proteins).

A value of 1.0 was given to the SF if a Cys was predicted with its sulfur atom among the first 3 sites, as ranked by Q-site finder. A value of 0.5 was given to the SF if the sulfur atom was predicted in the 4^th^, 5^th^ or 6^th^ site. A value of 0.25 was given if the sulfur atom was predicted in one of the remaining sites. If a residue was predicted in more than one site, only the highest ranked site was considered.

### Cys Reactivity: Theoretical Cys Titration

H++ server calculations were performed by choosing the following parameters: the interior dielectric constant (protein ε) was set to 20 while the solution dielectric constant was set to 75. Salinity (sodium chloride) of the medium was set to 150 mM. Of the H++ server output files, we considered only the *.pkaout files, which contained a list of all titrable residues with their pKa values. In addition, the files contained two-dimensional coordinates of theoretical titration curves for each residue. Parsing the H++ output file with an ad hoc Python tool, the values for the residue (in our case, Cys) were extracted, as well as its calculated pKa. We further considered the Henderson-Hasselbach (HH) equation:
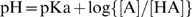
(2)Equation 2 can be rewritten to show the charge on the titrable residue
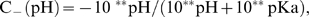
(3)where C_−_ indicates a negative charge on the sulfur atom. Equation 3 is valid for acidic residues, which acquire negative charge upon titration (e.g., Cys). Substituting H++ pKa-calculated value in Equation 3 and varying the pH between 0 and 18, the HH behaving curve for an acidic residue was then obtained. Figure 5 shows two examples of superimposition of theoretical titration curves obtained by the H++ server (red curves) and the corresponding HH behavior curves (blue curves). The HH behavior curves were viewed as standard behavior of the residue if no perturbations due to other nearby titrable residues occurred [15]. Thus, deviation of the red curve from the blue curve in Figure 5 (in the titrable range around the pKa) pinpointed the active site residue [16],[17]. Automatic evaluation of the deviation between the two curve behaviors could be a challenge [27]. In the present work, we were only interested in a simple way to perform a quick quantification of the deviation between the two curves. Thus, point by point subtraction (for each pH value) between the two curves was carried on. These values were integrated over the entire pH range, resulting in the overall difference absolute value (Σ Δ) for the deviation between the two curves.

Σ Δ was next evaluated to give a contribution to the SF. Cutoff values employed were as follows: if |Σ Δ|≥2.0, then a value of 1.0 is given. If 2.0>|Σ Δ|≥1.5, a value of 0.75 is given. If 1.5>|Σ Δ|≥1.0, a value of 0.5 is given. If 1.0>|Σ Δ|≥0.5, value of 0.25 is given. Values below 0.5 correspond to a small or null deviation from the typical HH titration behavior (Figure 5B), and consequently a zero value is given to SF. The overall contribution of the Cys Reactivity method to the SF ranged from 0 to 2.0.

Finally, in the complete algorithm, the resulting value of the SF ranged from 0 to 6.0 (Figure 1A). We found that a value of 2.75 was a minimum cutoff value that positively discriminated catalytic redox-active Cys residues (Figure 7 and Figure 8).

## Supporting Information

Figure S1 Structural profile scoring of the dataset. For each thiol oxidoreductase in the dataset (Table S1), a structural profile of the active site was generated, following a published procedure (Cammer et al., 2003). Dataset S1 is shown by blue circles, Dataset S2 by green squares, and representatives of the Random Cys set 2 by yellow squares. Each protein was scanned against all other thiol oxidoreductases in the dataset (i.e., no protein was scanned against itself). Yellow square proteins were randomly chosen among the Random Cys set, as described in the main text and Figure 2. The Score of the figure refers to the application of Equation 1 (see Methods section) with our ad hoc optimized SI, Sw, Ss, Sg parameters. The dashed line represents the lowest cutoff value (0.4) that avoids false positives in our structural profiling analysis.
(2.18 MB EPS)

Figure S2 Profile scoring analysis for proteins in our dataset. The score refers to the output of Equation 1 (Eq 1), as described in the Methods section. Three results are shown: (a) application of Eq 1 to the dataset with original parameters (shown by red triangles), (b) application of Eq 1 with optimized parameters (shown by filled green circles), and (c) application of Eq 1 with intermediate parameters (SI = 1, Ss = 0.2, Sw = 0.1,Sg = −.025, see Methods section) (shown by blue rhombi). Thiol oxidoreductases are shown on the left side of the graph (proteins with higher scores); on the right side are scored proteins of the controls sets (Dataset S2 and representatives of the Random Cys set). A trend line (solid lines in the graph) is drawn for each of the three different calculations (green for Eq 1 with optimized parameters, red for Eq 1 with original parameters, and blue for Eq 1 with “intermediate parameters”). A better resolution is obtained with the optimized parameters, as the thiol oxidoreductases show on average higher scores, without proportionally affecting the false positive prediction rate (i.e., trend lines on the right side of the graph are closer than those referred to thiol oxidoreductases).
(2.69 MB EPS)

Figure S3 Secondary structure composition. Average secondary structure composition (with calculated standard deviations) for the active sites of our datasets (S1, S2, and representative of the Random Cys set).
(1.24 MB EPS)

Figure S4 Analysis of the dataset with the Active Site Similarity method. The scoring function (SF) as defined in the Methods section includes only the Similarity method (i.e., the Cys Reactivity contribution was set to zero). A good separation of thiol oxidoreductases (shown by blue circles) from other proteins in the dataset was achieved (green squares correspond to non-thiol oxidoreductases of Dataset S2, and yellow squares show a set of randomly chosen proteins). Only one protein of the non-redox catalytic Cys dataset (S2) showed a score comparable to that of thiol oxidoreductases: this protein is a low molecular weight phosphotyrosine phosphatase with PDB code 1D1P. Enzymes of this family are known to contain a nucleophilic catalytic Cys which is subject to redox regulation. The score obtained for 1D1P is due to a good structural profile pairwise alignment (profile score 0.55, see Methods section and the main text) with the wild type S. aureus arsenate reductase with PDB code 1LJL. Similarity between low molecular weight phosphotyrosine phosphatases and some arsenate reductases is discussed in detail in the main text.
(1.95 MB EPS)

Figure S5 Distribution of pKa of catalytic Cys and sulfur atom exposure. The data for proteins in Datasets S1 and S2 are shown. Blue circles correspond to thiol oxidoreductases and green rhombi to proteins containing catalytic non-redox Cys. Catalytic Cys pKa values were calculated with PropKa 2.0, and sulfur atom exposure was calculated with Surface 4.0.
(2.93 MB EPS)

Figure S6 Optimization of parameter weights. Proteins of the Test Case (Table S3) were tested with our procedure, varying the weight of each component in the algorithm shown in Figure 1. (A) Scores for each protein are shown when all parameter weights were set to 1 (i.e., each part of the procedure contributed equally). Thiol oxidoreductases are shown by green circles and control proteins by grey circles. This was the starting point of our analysis, after which we changed each parameter to optimize for separation of thiol oxidoreductases and other proteins. (B) The following parameter weights were used: secondary structure (IIstr) = 1, compositional analysis (Compo) = 1, profile scoring (Profile) = 2, Q-site finder analysis (Qsite) = 1, H++ analysis (H++) = 1. Increasing considerably the weight of the profile scoring proved to be important for the improved performance. (C) Further improvement of the performance is shown obtained by decreasing the secondary structure weight to 0.75 and increasing the profile scoring weight to 2.25. Variation of other parameter weights in the range 0.5–2.0 did not improve performance. (D) We found the best resolution with the relative weights described in the text (which we call optimized parameters), corresponding to the following weight distribution: IIstr = 0.5, Compo = 1, Profile = 2.5, Qsite = 1, H++ = 1.
(2.14 MB EPS)

Table S1 Thiol oxidoreductases.
(0.03 MB PDF)

Table S2 Enzymes with catalytic non-redox Cys.
(0.02 MB PDF)

Table S3 Test Case.
(0.04 MB PDF)

Table S4 Details of homology modeling of yeast proteins.
(0.18 MB PDF)

Table S5 Detailed results for the yeast proteome.
(0.04 MB PDF)

Table S6 Predicted yeast thiol oxidoreductases.
(0.04 MB PDF)
