# Supplementary material for: A Structure-Based Approach for Detection of Thiol Oxidoreductases and Their Catalytic Redox-Active Cysteine Residues
Source: PLoS Comput Biol. 2009 May 8;5(5):e1000383. doi: 10.1371/journal.pcbi.1000383 (PMC2673044; doi:10.1371/journal.pcbi.1000383)
Supplement: Table S1 — Thiol oxidoreductases. (0.03 MB PDF) [file pcbi.1000383.s007.pdf]

Table S1. Thiol oxidoreductases

PDB ID	Protein name (pdb header)	SOURCE	FOLD	Catalytic Cys (pdb numbering)
**1DBY**	Chloroplast Thioredoxin	*Chlamydomonas reinhardtii*	Thioredoxin	31
**1QUW**	Thioredoxin	*Alicyclobacillus acidocaldarius*	Thioredoxin	29
**1TI3**	Thioredoxin	*Populus tremula*	Thioredoxin	38
**1XOA**	Thioredoxin	*Escherichia coli*	Thioredoxin	32
**1XW9**	Thioredoxin	*Drosophila melanogaster*	Thioredoxin	32
**2GZY**	Thioredoxin	*Bacillus subtilis*	Thioredoxin	29
**3TRX**	Thioredoxin	*Homo sapiens*	Thioredoxin	32
**1DE1**	Glutaredoxin	*Bacteriophage T4*	Thioredoxin	14
**1WJK**	Glutaredoxin	*Mus musculus*	Thioredoxin	27
**1EGO**	Glutaredoxin	*Escherichia coli*	Thioredoxin	11
**1Z7R**	Glutaredoxin	*Populus tremula*	Thioredoxin	31
**2FLS**	Glutaredoxin 2	*Homo sapiens*	Thioredoxin	37
**1QQ2**	Peroxiredoxin 2	*Rattus norvegicus*	Thioredoxin	52
**2PN8**	Peroxiredoxin 4	*Homo sapiens*	Thioredoxin	124
**1OC3**	Peroxiredoxin 5	*Homo sapiens*	Thioredoxin	47
**1TP9**	Peroxiredoxin 2	*Populus. tremula*	Thioredoxin	51
**1XXU**	AhpE (1-cys peroxiredoxin)	*Mycobacterium tuberculosis*	Thioredoxin	45
**2C0D**	Peroxiredoxin 2	*Plasmodium falciparum*	Thioredoxin	67
**1X5D**	Protein Disulfide Isomerase (PDI)	*Homo sapiens*	Thioredoxin	37
**2DJJ**	PDI	*Humicola insolens*	Thioredoxin	37
**1MEK**	PDI Beta subunit	*Homo sapiens*	Thioredoxin	36
**1A2J**	DsbA-II	*Escherichia coli*	Thioredoxin	30
**1V57**	DsbG	*Escherichia coli*	Thioredoxin	109
**1JZD**	DsbC-DsbDalpha complex	*Escherichia coli*	Thioredoxin	98
**1BED**	DsbA	*Vibrio cholera*	Thioredoxin	30
**2OBK**	SelW	*Pseudomonas fluorescens*	Thioredoxin	16
**1PSQ**	Thiol peroxidase (atypical 2 cys peroxiredoxin)	*Streptococcus pneumoniae*	Thioredoxin	58
**2Y25**	Thiol peroxidase	*Mycobacterium tuberculosis*	Thioredoxin	60*
**2Z9S**	Peroxiredoxin 1	*Rattus norvegicus*	Thioredoxin	52*
**1Q98**	Thiol peroxidase	*Haemophilus influenzae*	Thioredoxin	59
**1QXH**	Thiol peroxidase	*Escherichia coli*	Thioredoxin	61
**1I9D**	Arsenate Reductase (ArsC)	*Escherichia coli*	Thioredoxin	12
**2P5R**	glutathione peroxidase 5	*Populus tremula*	Thioredoxin	44
**2P31**	glutathione peroxidase 7	*Homo sapiens*	Thioredoxin	57
**2FWE**	C-terminal domain DsbD gamma	*Escherichia coli*	Thioredoxin	461
**2GS3**	glutathione peroxidase 4	*Homo sapiens*	Thioredoxin	73
**2HE3**	glutathione peroxidase 2	*Homo sapiens*	Thioredoxin	40
**2I3Y**	glutathione peroxidase 5	*Homo sapiens*	Thioredoxin	73
**1GER**	Glutathione reductase	*Escherichia coli*	FAD/NAD(P)-binding domain	42
**1GRT**	Glutathione reductase	*Homo sapiens*	FAD/NAD(P)-binding domain	58
**1ONF**	Glutathione reductase	*Plasmodium falciparum*	FAD/NAD(P)-binding domain	39
**1AOG**	Trypanothione reductase	*Trypanosoma cruzi*	FAD/NAD(P)-binding domain	53

**1H6V**	Thioredoxin reductase	*Rattus norvegicus*	FAD/NAD(P)-binding domain	59
**2NVK**	Thioredoxin reductase	*Drosophila melanogaster*	FAD/NAD(P)-binding domain	62
**1VDC**	Thioredoxin reductase	*Arabidopsis thaliana*	FAD/NAD(P)-binding domain	138
**2Q0K**	Thioredoxin reductase	*Helicobacter pylori*	FAD/NAD(P)-binding domain	136
**2A87**	Thioredoxin reductase	*Mycobacterium tuberculosis*	FAD/NAD(P)-binding domain	148
**1TRB**	Thioredoxin reductase	*Escherichia coli*	FAD/NAD(P)-binding domain	105
**1JR8**	Erv2p	*Saccharomyces cerevisiae*	ERO1-like	83
**2HJ3**	Erv1p	*Arabidopsis thaliana*	Four-helical up-and-down bundle	83
**1RP4**	Ero1p	*Saccharomyces cerevisiae*	Four-helical up-and-down bundle	352
**1XM0**	Methionine sulfoxide reductase B (MsrB)	*Bacillus subtilis*	Mss4-like	115
**1L1D**	Pil MsrB	*Neisseria gonorrhoeae*	Mss4-like	495
**1NWA**	MsrA	*Mycobacterium tuberculosis*	Ferredoxin-like	13
**2GT3**	MsrA	*Escherichia coli*	Ferredoxin-like	51
**1FVA**	MsrA	*Bos taurus*	Ferredoxin-like	72
**2J89**	MsrA	*Populus tremula*	Ferredoxin-like	46
**1VHM**	Free Methionine sulfoxide reductase GAF domain	*Escherichia coli*	Profilin-like	118
**1F5M**	Free Methionine sulfoxide reductase GAF domain	*Saccharomyces cerevisiae*	Profilin-like	125
**1LJL**	Arsenate reductase	*Staphylococcus aureus*	Phosphotyrosine protein phosphatases I-like	10
**1Y1L**	Arsenate reductase	*Archaeoglobus fulgidus*	Phosphotyrosine protein phosphatases I-like	6
**1JL3**	Arsenate reductase	*Bacillus subtilis*	Phosphotyrosine protein phosphatases I-like	10
**1QWI**	OsmC	*Escherichia coli*	OsmC-like	59
**1ZB9**	Organic peroxide resistance protein (OhR)	*Xylella fastidiosa*	OsmC-like	61
**2OPL**	OsmC	*Geobacter sulfurreducens*	OsmC-like	56
**2D7V**	OsmC-like Protein VCA03	*Vibrio cholerae*	OsmC-like	76
**1USP**	OhR	*Deinococcus radiodurans*	OsmC-like	57
**1N2F**	OhR	*Pseudomonas aeruginosa*	OsmC-like	60
**2PRR**	alkyl hydroperoxidase (AhpD)	*Ralstonia eutropha*	AhpD-like	89
**2OUW**	AhpD	*Rhodospirillum rubrum*	AhpD-like	86
**2CWQ**	conserved protein TTHA0727 (AhpD-like)	*Thermus thermophilus*	AhpD-like	73
**2GMY**	AhpD	*Agrobacterium tumefaciens*	AhpD-like	51
**1KNC**	AhpD	*Mycobacterium tuberculosis*	AhpD-like	133
**1JPE**	DsbD-alpha (N-terminal domain of DsbD)	*Escherichia coli*	Immunoglobulin-like beta-sandwich	109

* These proteins have the catalytic Cys mutated to serine in the structure. They have been *in silico* mutated to restore the catalytic Cys, with standard modeling techniques (see Methods in the main text)
