# Supplementary material for: A Structure-Based Approach for Detection of Thiol Oxidoreductases and Their Catalytic Redox-Active Cysteine Residues
Source: PLoS Comput Biol. 2009 May 8;5(5):e1000383. doi: 10.1371/journal.pcbi.1000383 (PMC2673044; doi:10.1371/journal.pcbi.1000383)
Supplement: Table S2 — Enzymes with catalytic non-redox Cys. (0.02 MB PDF) [file pcbi.1000383.s008.pdf]

Table S2. Enzymes with catalytic non-redox Cys

PDB ID	Protein name (pdb header)	SOURCE	FOLD	Catalytic Cys (pdb numbering)
**1PE6**	*Papain*	*Carica papaya*	*Cysteine proteinases*	*25*
**1DF0**	*M-calpain*	*Rattus norvegicus*	*Cysteine proteinases*	*105*
**1DKI**	*Zymogen form of Exotoxin B*	*Streptococcal pyrogenic exotoxin*	*Cysteine proteinases*	*47*
**1CMX**	*Ubiquitin YUH1-UBAL*	*Homo sapiens*	*Cysteine proteinases*	*90*
**1QMY**	*FMD Virus leader protease*	*Foot-and-mouth disease virus*	*Cysteine proteinases*	*51*
**1CV8**	*Staphopain*	*Staphylococcus aureus*	*Cysteine proteinases*	*24*
**1ICE**	*Caspase-1*	*Rattus norvegicus*	*Caspase-like*	*285*
**1CVR**	*Gingipain R*	*Porphyromonas gingivalis*	*Caspase-like*	*244*
**1AVP**	*Adenovirus 2 proteinase*	*Adenovirus type 2*	*Cysteine proteinases*	*122*
**1EUV**	*ULP1 endopeptide*	*Saccharomyces cerevisiae*	*Cysteine proteinases*	*580*
**1AUG_**	*Pyroglutamyl peptidase I*	*Bacillus amyloliquefaciens*	*Phosphorylase/hydrolase-like*	*144*
**1AT0**	*hedgehog autoprocessing domain (Hhc)*	*Drosophila melanogaster*	*Hedgehog/intein (Hint) domain*	*122*
**1HAV**	*Picornain 3C*	*Hepatitis A virus*	*Trypsin-like serine proteases*	*172*
**1GFY**	*Protein Tyrosine phosphatase (PTP)1B*	*Homo sapiens*	*(Phosphotyrosine protein) phosphatases II*	*258*
**1D5R**	*PTEN*	*Homo sapiens*	*(Phosphotyrosine protein) phosphatases II*	*124*
**2FEK**	*PTP*	*Escherichia coli*	*Phosphotyrosine protein phosphatases I-like*	*9*
**1YPV**	*Thymidylate synthase*	*Homo sapiens*	*Thymidylate synthase/dCMP hydroxymethylase*	*195*
**1TJS**	*Thymidylate synthase*	*Escherichia coli*	*Thymidylate synthase/dCMP hydroxymethylase*	*146*
**1YTS**	*PTP*	*Yersinia enterocolitica*	*Phosphotyrosine protein phosphatases I-like*	*403*
**2I6P**	*PTP*	*archaeal sulfolobus*	*(Phosphotyrosine protein) phosphatases II*	*96*
**1JLN**	*PTP*	*Mus musculus*	*(Phosphotyrosine protein) phosphatases II*	*480*
**1D1P**	*Low molecular weight PTP*	*Saccharomyces cerevisiae*	*Phosphotyrosine protein phosphatases I-like*	*13*
**2NQA**	*Calpain*	*Homo sapiens*	*Cysteine proteinases*	*105*
**1QXP**	*mu-like calpain*	*Rattus norvegicus*	*Cysteine proteinases*	*105*
**3BCN**	*Papain-like Ervatamin-A*	*Ervatamia coronaria*	*Cysteine proteinases*	*25*
**2E00**	*bleomycin hydrolase*	*Saccharomyces cerevisiae*	*Cysteine proteinases*	*73*
**1M72**	*Caspase-1*	*Spodoptera frugiperda*	*Caspase-like*	*178*
**2HBY**	*Caspase-1*	*Homo sapiens*	*Caspase-like*	*285*
**2FO5**	*Barley cysteine endoprotease B isoform 2*	*Hordeum vulgare*	*Cysteine proteinases*	*28*
**1GCB**	*Dna binding protease*	*Saccharomyces cerevisiae*	*Cysteine proteinases*	*73*
**2Z3E**	*CoV 3C-Like peptidase*	*SARS Coronavirus*	*Trypsin-like serine proteases*	*145*
**2OIV**	*XopD, Ubiquitin-like Protein Protease*	*Xanthomonas campestris*	*Cysteine proteinases*	*470*
**2BDZ**	*Mexicain*	*Jacaratia mexicana*	*Cysteine proteinases*	*25*
**2A5I**	*Human_SARS coronavirus main peptidase*	*SARS Coronavirus*	*Trypsin-like serine proteases*	*145*
**1JQP**	*Cathepsin C*	*Rattus norvegicus*	*Cysteine proteinases*	*233*

**2DJG**	*Dipeptidyl peptidase I*	*Homo sapiens*	*Cysteine proteinases*	*234*
**1YOV**	*Ubiquitin-activating enzyme (E1 enzyme)*	*Homo sapiens*	*Activating enzymes of the ubiquitin-like proteins*	*216*
