# Supplementary material for: A Structure-Based Approach for Detection of Thiol Oxidoreductases and Their Catalytic Redox-Active Cysteine Residues
Source: PLoS Comput Biol. 2009 May 8;5(5):e1000383. doi: 10.1371/journal.pcbi.1000383 (PMC2673044; doi:10.1371/journal.pcbi.1000383)
Supplement: Table S3 — Test Case. (0.04 MB PDF) [file pcbi.1000383.s009.pdf]

Table S3. Test Case

Source	PDB hit	gi	template (%id homology modeling template)	FOLD	Cys position	Active Site Similarity score	Cys Reactivity score	Combined score
Pyrococcus horikoshii	1J08			thioredoxin	149	1.5	1.75	3.25
Mycoplasma pneumoniae	1LQL			OsmC-like	252	1.5	1.25	2.75
Escherichia coli	1SK0			thioredoxin	12	2.5	1	3.5
Bos taurus Mitochondrial	1ZYE			thioredoxin	47	3.5	0.5	4
Neisseria meningitidis	2FY6			thioredoxin	67	2.5	1	3.5
Neisseria gonorrhoeae	2H30			thioredoxin	68	2.5	1	3.5
Ectromelia virus	2HZF			thioredoxin	23	2	1.25	3.25
Saccharomyces cerevisiae	2JAC			thioredoxin	30	1.5	1.5	3
Pseudomonas aeruginosa	2O4D			AhpD-like	51	3.5	2	5.5
Silicibacter sp. TM1040	2PFX			thioredoxin	88	3.5	2	5.5
Aquifex aeolicus	2YWM			thioredoxin	151	1.625	1.25	2.875
Clostridium kluyveri		*gi|153955052*	1XM0(62%)	Mss4-like	119	2.25	2	4.25
Rattus norvegicus		*gi|16758004*	1FVA (85%)	Ferredoxin-like	72	3.5	2	5.5
Xenopus laevis		*gi|148235445*	1FVA (70%)	Ferredoxin-like	51	3.5	2	5.5
Bos taurus	1GP1			thioredoxin	45	2.75	0.5	3.25
Bacillus cereus		*gi|52143310*	2P5Q(48%)	thioredoxin	35	2.25	1.75	4
Drosophila melanogaster		*gi|24656772*	2P5R(51%)	thioredoxin	49	2.25	0.75	3
Escherichia coli	1TRB			FAD/NAD-linked reductases	135	1.25	2	3.25
Mus musculus	1ZKQ			FAD/NAD-linked reductases	86	1.25	2	3.25
Crithidia fasciculata	2TPR			FAD/NAD-linked reductases	51	1	2	3
Oryza Sativa		*gi|4106694*	1FEC(44%)	FAD/NAD-linked reductases	70	1	2	3
Saccharomyces cerevisiae	1A6R			Papain-like	73	0	0.5	0.5
Homo sapiens	1CB5			Papain-like	73	0	0	0
Homo sapiens	1NL6			Cysteine proteinases	25	0.5	1.25	1.75
Homo sapiens	1XD3			Cysteine proteinases	95	0.5	1.75	2.25
Cryptococcus neoformans	2AAZ			Thymidylate synthase	187	0	1.25	1.25
Selenomonas ruminantium	2B4U			(Phosphotyrosine protein)	252	0	1.25	1.25
Bos taurus	2DCC			Cysteine proteinases	29	0	0.75	0.75
Homo sapiens	2ETL			Cysteine proteinases	90	0	1.75	1.75
Campylobacter jejuni	2GI4			(Phosphotyrosine protein)	8	0	1	1
Fasciola hepatica	2O6X			Cysteine proteinases	116	0	1	1
Cryptosporidium hominis	2OIP			Thymidylate synthase	402	0.5	1.5	2
Mycobacterium tuberculosis	2OZ5			(Phosphotyrosine protein)	160	0	2	2
Lactobacillus casei	2TDM			Thymidylate synthase	198	0.5	1.5	2
Homo sapiens	2D2Z			Glutathione S-transferase (GST), C-terminal Domain	234	1.25	1	2.25
Rattus norvegicus	1FLJ			Carbonic anhydrase	188	0	0	0
Homo sapiens	1HNL			Lysozyme-like	95	1.25	1	2.25
Oryctolagus cuniculus	1IJJ			Ribonuclease H-like motif	374	0.5	0.75	1.25
Homo sapiens	1KCG			C-type lectin-like	109	0.5	0.75	1.25
Homo sapiens	1EEM			Glutathione S-transferase (GST), thioredoxin-like domain	32	1.5	1	2.5
Homo sapiens	2O02			alpha-alpha superhelix	25	0.5	0.5	1
Homo sapiens	1W7B			Annexin	294	1.25	1	2.25
Homo sapiens		*gi|1703322*	1ANN(59%)	Annexin	133	0.5	1	1.5

Ovis aries	1UW3			Prion-like	148	1.5	0	1.5
Homo sapiens		*gi|23512328*	2OVR 34%)	Ferredoxin-like	245	0	0.5	0.5
Homo sapiens		*gi|119172*	1NOU(64%)	Reductase/isomerase/	567	0.5	0	0.5
Homo sapiens		*gi|55584035*	1F60(81%)	Reductase/isomerase/elongation factor common domain	234	0.25	1.5	1.75
Homo sapiens		*gi|21264428*	1YUW(55%)	Ribonuclease H-like motif	487	0	0	0
Homo sapiens		*gi|6166599*	2AKA(48%)	P-loop containing nucleoside triphosphate hydrolases	91	0.25	0.25	0.5
Homo sapiens	1CYN			Cyclophilin-like	202	0.25	0.75	1
Homo sapiens	2GF9			P-loop containing nucleoside triphosphate hydrolases	184	0.5	0.75	1.25
Homo sapiens		*gi|730922*	1Q3R(38%)	GroEL-intermediate domain like /Cystatin like domain	406	0.75	0	0.75
Homo sapiens		*gi|74762725*	1XTD(46%)	SH3-like barrel	73	0	1	1
Homo sapiens	1TR2			Four-helical up-and-down	85	0.25	0	0.25
