# Supplementary material for: A Structure-Based Approach for Detection of Thiol Oxidoreductases and Their Catalytic Redox-Active Cysteine Residues
Source: PLoS Comput Biol. 2009 May 8;5(5):e1000383. doi: 10.1371/journal.pcbi.1000383 (PMC2673044; doi:10.1371/journal.pcbi.1000383)
Supplement: Table S4 — Details of homology modeling of yeast proteins. (0.18 MB PDF) [file pcbi.1000383.s010.pdf]

Table S4. Details of homology modeling of yeast proteins

```
>gi|6319522|ref|NP_009604.1| Homology to rat S11, human S11, and E. coli S17; Rps11bp
[Saccharomyces cerevisiae]
MSTELTVQSERAFQKQPHIFNNPKVKTSKRTKRWYKNAGLGFKTPKTAIEGSYIDKKCPFTGLVSIRGKI
LTGTVVSTKMHRTIVIRRAYLHYIPKYNRYEKRHKNVPVHVSPAFRVQVGDIVTVGQCRPISKTVRFNVV
KVSAAAGKANKQFAKF
```

Modeled residue range: 69 to 142 based on template **2j00Q** (2.80 Å) Sequence Identity [%]: 39 Evalue: 6.85e-6

```
>gi|6319726|ref|NP_009808.1| 3-deoxy-D-arabino-heptulosonate 7-phosphate (DAHP) synthase
isoenzyme; Aro4p [Saccharomyces cerevisiae]
MSESPMFAANGMPKVNQGAEEDVRILGYDPLASPALLQVQIPATPTSLETAKRGRREAIDIITGKDDRVL
VIVGPCSIHDLEAAQEYALRLKKLSDELKGDLSIIMRAYLEKPRTTVGWKGLINDPDVNNTFNINKGLQS
ARQLFVNLTNIGLPIGSEMLDTISPQYLADLVSFGAIGARTTESQLHRELASGLSFPVGFKNGTDGTLNV
AVDACQAAAHSHHFMGVTKHGVAAITTTKGNEHCFVILRGGKKGTNYDAKSVAEAKAQLPAGSNGLMIDY
SHGNSNKDFRNQPKVNDVVCEQIANGENAITGVMIESNINEGNQGIPAEGKAGLKYGVSITDACIGWETT
EDVLRKLAAAVRQRREVNKK
```

Modeled residue range: 23 to 369 based on template **1of8B** (1.50 Å) Sequence Identity [%]: 99 Evalue: 0.00e-1

```
>gi|6319966|ref|NP_010046.1| Ypd1p is an intermediate protein between Sln1p and Ssk1p in the
phosphorelay reaction.; Ypd1p [Saccharomyces cerevisiae]
MSTIPSEIINWTILNEIISMDDDDSDFSKGLIIQFIDQAQTTFAQMQRQLDGEKNLTELDNLGHFLKGSS
AALGLQRIAWVCERIQNLGRKMEHFFPNKTELVNTLSDKSIINGINIDEDDEEIKIQVDDKDENSIYLIL
IAKALNQSRLEFKLARIELSKYYNTNL
```

Modeled residue range: 2 to 167 based on template **1c02B** (1.80 Å) Sequence Identity [%]: 99 Evalue: 4.73e-75

```
>gi|6320210|ref|NP_010290.1| Note that the sequence of TRP1 from strain S228C, which is the
sequence stored in SGD, contains an ochre mutation at codon 67.; Trp1p [Saccharomyces cerevisiae]
MSVINFTGSSGPLVKVCGLQSTEAAECALDSDADLLGIICVPNRKRTIDPVIARKISSLVKAYKNSSGTP
KYLVGVFRNQPKEDVLALVNDYGIDIVQLHGDESWQEYQEFLGLPVIKRLVFPKDCNILLSAASQKPHSF
IPLFDSEAGGTGELLDWNSISDWVGRQESPESLHFMLAGGLTPENVGDALRLNGVIGVDVSGGVETNGVK
DSNKIANFVKNAKK
```

Modeled residue range: 14 to 223 based on template **1nsjA** (2.00 Å) Sequence Identity [%]: 33 Evalue: 1.28e-19

```
>gi|6320228|ref|NP_010308.1| Homology to rat S11, human S11, and E. coli S17; Rps11ap
[Saccharomyces cerevisiae]
MSTELTVQSERAFQKQPHIFNNPKVKTSKRTKRWYKNAGLGFKTPKTAIEGSYIDKKCPFTGLVSIRGKI
LTGTVVSTKMHRTIVIRRAYLHYIPKYNRYEKRHKNVPVHVSPAFRVQVGDIVTVGQCRPISKTVRFNVV
KVSAAAGKANKQFAKF
```

Modeled residue range: 49 to 142 based on template **2j00Q** (2.80 Å) Sequence Identity [%]: 39 Evalue: 6.85e-6

```
>gi|6320720|ref|NP_010801.1| Glutaredoxin (thioltransferase) (glutathione reductase); Ttr1p
[Saccharomyces cerevisiae]
METNFSFDSNLIVIIIITLFATRIIAKRFLSTPKMVSQETVAHVKDLIGQKEVFVAAKTYCPYCKATLST
LFQELNVPKSKALVLELDEMSNGSEIQDALEEISGQKTVPNVYINGKHIGGNSDLETLKKNGKLAEILKP
```

VFQ

Modeled residue range: 35 to 126 based on template **2jacA** (2.02 Å) Sequence Identity [%]: 67 Evalue: 1.85e-32

>gi|6320796|ref|NP_010875.1| iso-2-cytochrome c; Cyc7p [Saccharomyces cerevisiae]
MAKESTGFKPGSAKKGATLFKTRCQQCHTIEEGGPNKVGPNLHGIFGRHSGQVKGYSYTDANINKNVKWD
EDSMSEYLTNPKKYIPGTKMAFAGLKKEKDRNDLITYMTKAAK

Modeled residue range: 2 to 113 based on template **1yeaA** (1.90 Å) Sequence Identity [%]: 100 Evalue: 1.72e-54

>gi|6320998|ref|NP_011077.1| Stationary Phase Induced; strongly expressed during stationary phase, and trancription is dependent on MSN2/MSN4.; Spi1p [Saccharomyces cerevisiae]
MLSNAKLLLSLAMASTALGLVSNSSSSVIVVPSSDATIAGNDTATPAPEPSSAAPIFYNSTATATQYEVV
SEFTTYCPEPTTFVTNGATFTVTAPTTLTITNCPCTIEKPTSETSVSSTHDVETNSNAANARAIPGALGL
AGAVMMLL

Modeled with HOMER
Modeled residue range: 18 to 89 based on template **2VC9|A** (2.36 Å)
Sequence Identity [%]: 26 PDB Blast Evalue: 1.0

>gi|6321022|ref|NP_011101.1| Member of a glutaredoxin subfamily in Sc together with GRX3 & GRX5. Significant sequence diff. with the other glutaredoxin subfamily, formed by the previously described GRX1 & GRX2 glutaredoxins (Luikenhuis MBC 9:1081, 1998); Grx4p [Saccharomyces cerevisiae]
MTVVEIKSQDQFTQLTTTNAANKLIVLYFKAQWADPCKTMSQVLEAVSEKVRQEDVRFLSIDADEHPEIS
DLFEIAAVPYFVFIQNGTIVKEISAADPKEFVKSLEILSNASASLANNAKGPKSTSDEESSGSSDDEEDE
TEEEINARLVKLVQAAPVMLFMKGSPSEPKCGFSRQLVGILREHQIRFGFFDILRDENVRQSLKKFSDWP
TFPQLYINGEFQGGLDIIKESIEEDPEYFQHALQ

C-term: Modeled residue range: 152 to 230 based on template **1wikA** (99.9 Å)
Sequence Identity [%]: 58 Evalue: 5.03e-22
N-term: Modeled residue range: 2 to 101 based on template **2j23A** (1.41 Å)
Sequence Identity [%]: 36 Evalue: 1.57e-12

>gi|14318471|ref|NP_116606.1| 47 kDa type I transmembrane protein localized to the Golgi; Emp47p [Saccharomyces cerevisiae]
MMMLITMKSTVLLSVFTVLATWAGLLEAHPLGDTSDASKLSSDYSLPDLINARKVPNNWQTGEQASLEEG
RIVLTSKQNSKGSLWLKQGFDLKDSFTMEWTFRSVGYSGQTDGGISFWFVQDSNVPRDKQLYNGPVNYDG
LQLLVDNNGPLGPTLRGQLNDGQKPVDKTKIYDQSFASCLMGYQDSSVPSTIRVTYDLEDDNLLKVQVDN
KVCFQTRKVRFPSGSYRIGVTAQNGAVNNNAESFEIFKMQFFNGVIEDSLIPNVNAMGQPKLITKYIDQQ
TG KEKLIEKTAFDADKDKITNYELYKKLDRVEGKILANDINALETKLNDVIKVQQELLSFMTTITKQLSS
KPPANNEKGTSTDDAIAEDKENFKDFLSINQKLEKVLVEQEKYREATKRHGQDGPQVDEIARKLMIWLLP
LIFIMLVMAYYTFRIRQEIIKTKLL

Modeled residue range: 28 to 282 based on template **2A6Y|A** (1.42 Å)
Sequence Identity [%]: 98 Evalue: 4.0 e-50

>gi|14318550|ref|NP_116683.1| Establishment of COhesion; Eco1p [Saccharomyces cerevisiae]
MKARKSQRKAGSKPNLIQSKLQVNNGSKSNKIVKCDKCEMSYSSTSIEDRAIHEKYHTLQLHGRKWSPNW
GSIVYTERNHSRTVHLSRSTGTITPLNSSPLKKSSPSITHQEEKIVYVRPDKSNGEVRAMTEIMTLVNNE
LNAPHDENVIWNSTTEEKGKAFVYIRNDRAVGIIIIENLYGGNGKTSSRGRWMVYDSRRLVQNVYPDFKI
GISRIWVCRTARKLGIATKLIDVARENIVYGEVIPRYQVAWSQPTDSGGKLASKYNGIMHKSGKLLLPVY

Modeled with HOMER
Modeled residue range: 23 to 98 based on template **1E58|A** (1.25 Å)
Sequence Identity [%]: 23 PDB Blast Evalue: 2.3

>gi|14318558|ref|NP_116691.1| ubiquinol-cytochrome c oxidoreductase subunit 6 (17 kDa); Qcr6p [Saccharomyces cerevisiae]
MGMLELVGEYWEQLKITVVPVVAAAEDDDNEQHEEKAAEGEEKEEENGDEDEDEDEDEDDDDDDDEDEEE
EEEVTDQLEDLREHFKNTEEGKALVHHYEECAERVKIQQQQPGYADLEHKEDCVEEFFHLQHYLDTATAP
RLFDKLK

Modeled residue range: 74 to 147 based on template **1ezvH** (2.30 Å) Sequence Identity [%]: 100 Evalue: 5.93e-24

>gi|6321631|ref|NP_011708.1| Glyceraldehyde-3-phosphate dehydrogenase 3; Tdh3p [Saccharomyces cerevisiae]
MVRVAINGFGRIGRLVMRIALSRPNVEVVALNDPFITNDYAAYMFKYDSTHGRYAGEVSHDDKHIIVDGK
KIATYQERDPANLPWGSSNVDIAIDSTGVFKELDTAQKHIDAGAKKVVITAPSSTAPMFVMGVNEEKYTS
DLKIVSNASCTTNCLAPLAKVINDAFGIEEGLMTTVHSLTATQKTVDGPSHKDWRGGRTASGNIIPSSTG
AAKAVGKVLPELQGKLTGMAFRVPTVDVSVVDLTVKLNKETTYDEIKKVVKAAAEGKLKGVLGYTEDAVV
SSDFLGDSHSSIFDASAGIQLSPKFVKLVSWYDNEYGYSTRVVDLVEHVAKA

Modeled residue range: 1 to 330 based on template **2i5pP** (2.30 Å) Sequence Identity [%]: 78 Evalue: 3.53e-139
{25}

>gi|6321706|ref|NP_011783.1| First enzyme in biosynthetic pathway for folic acid and tetrahydrobioptern; Fol2p [Saccharomyces cerevisiae]
MHNIQLVQEIERHETPLNIRPTSPYTLNPPVERDGFSWPSVGTRQRAEETEEEEKERIQRISGAIKTILT
ELGEDVNREGLLDTPQRYAKAMLYFTKGYQTNIMDDVIKNAVFEEDHDEMVIVRDIEIYSLCEHHLVPFF
GKVHIGYIPNKKVIGLSKLARLAEMYARRLQVQERLTKQIAMALSDILKPLGVAVVMEASHMCMVSRGIQ
KTGSSTVTSCMLGGFRAHKTREEFLTLLGRRSI

Modeled residue range: 42 to 238 based on template **1fb1A** (3.10 Å) Sequence Identity [%]: 63 Evalue: 5.73e-65

>gi|6322180|ref|NP_012255.1| Derepression Of Telomeric silencing; Dot5p [Saccharomyces cerevisiae]
MGEALRRSTRIAISKRMLEEEESKLAPISTPEVPKKKIKTGPKHNANQAVVQEANRSSDVNELEIGDPIP
DLSLLNEDNDSISLKKITENNRVVVFFVYPRASTPGCTRQACGFRDNYQELKKYAAVFGLSADSVTSQKK
FQSKQNLPYHLLSDPKREFIGLLGAKKTPLSGSIRSHFIFVDGKLKFKRVKISPEVSVNDAKKEVLEVAE
KFKEE

Modeled residue range: 59 to 214 based on template **2a4vA** (1.80 Å) Sequence Identity [%]: 98 Evalue: 1.04e-76

>gi|6322409|ref|NP_012483.1| Glyceraldehyde-3-phosphate dehydrogenase 1; Tdh1p [Saccharomyces cerevisiae]
MIRIAINGFGRIGRLVLRLALQRKDIEVVAVNDPFISNDYAAYMVKYDSTHGRYKGTVSHDDKHIIIDGV
KIATYQERDPANLPWGSLKIDVAVDSTGVFKELDTAQKHIDAGAKKVVITAPSSSAPMFVVGVNHTKYTP
DKKIVSNASCTTNCLAPLAKVINDAFGIEEGLMTTVHSMTATQKTVDGPSHKDWRGGRTASGNIIPSSTG
AAKAVGKVLPELQGKLTGMAFRVPTVDVSVVDLTVKLEKEATYDQIKKAVKAAAEGPMKGVLGYTEDAVV
SSDFLGDTHASIFDASAGIQLSPKFVKLISWYDNEYGYSARVVDLIEYVAKA

Modeled residue range: 5 to 325 based on template **2i5pP** (2.30 Å) Sequence Identity [%]: 77 Evalue: 1.23e-139

>gi|6322559|ref|NP_012633.1| ubiquitin hydrolase; Yuh1p [Saccharomyces cerevisiae]
MSGENRAVVPIESNPEVFTNFAHKLGLKNEWAYFDIYSLTEPELLAFLPRPVKAIVLLFPINEDRKSSTS
QQITSSYDVIWFKQSVKNACGLYAILHSLSNNQSLLEPGSDLDNFLKSQSDTSSSKNRFDDVTTDQFVLN
VIKENVQTFSTGQSEAPEATADTNLHYITYVEENGGIFELDGRNLSGPLYLGKSDPTATDLIEQELVRVR
VASYMENANEEDVLNFAMLGLGPNWE

Modeled residue range: 6 to 234 based on template **1cmxA** (2.25 Å) Sequence Identity [%]: 93 Evalue: 5.16e-114

>gi|7839183|ref|NP_058168.1| Translocase of the inner membrane; mitochondrial intermembrane space protein mediating import and insertion of polytopic inner membrane proteins; Tim8p [Saccharomyces cerevisiae]
MSSLSTSDLASLDDTSKKEIATFLEGENSKQKVQMSIHQFTNICFKKCVESVNDSNLSSQEEQCLSNCVN
RFLDTNIRIVNGLQNTR

Modeled residue range: 28 to 86 based on template **3cjhB** (2.60 Å) Sequence Identity [%]: 100 Evalue: 4.14e-19

>gi|6322468|ref|NP_012542.1| glyceraldehyde 3-phosphate dehydrogenase; Tdh2p [Saccharomyces cerevisiae]
MVRVAINGFGRIGRLVMRIALQRKNVEVVALNDPFISNDYSAYMFKYDSTHGRYAGEVSHDDKHIIVDGH
KIATFQERDPANLPWASLNIDIAIDSTGVFKELDTAQKHIDAGAKKVVITAPSSTAPMFVMGVNEEKYTS
DLKIVSNASCTTNCLAPLAKVINDAFGIEEGLMTTVHSMTATQKTVDGPSHKDWRGGRTASGNIIPSSTG
AAKAVGKVLPELQGKLTGMAFRVPTVDVSVVDLTVKLNKETTYDEIKKVVKAAAEGKLKGVLGYTEDAVV
SSDFLGDSNSSIFDAAAGIQLSPKFVKLVSWYDNEYGYSTRVVDLVEHVAKA

Modeled residue range: 1 to 330 based on template **2i5pP** (2.30 Å) Sequence Identity [%]: 78 Evalue: 1.23e-139

>gi|6323103|ref|NP_013175.1| Bud20p [Saccharomyces cerevisiae]
MGRYSVKRYKTKRRTRDLDLIYNDLSTKESVQKLLNQPLDETKPGLGQHYCIHCAKYMETAIALKTHLKG
KVHKRRVKELRGVPYTQEVSDAAAGYNLNKFLNRVQEITQSVGPEKESNEALLKEHLDSTLANVKTTEPT
LPWAAADAEANTAAVTEAESTASAST

Modeled residue range: 36 to 95 based on template **1zr9A** (99.9 Å) Sequence Identity [%]: 45 Evalue: 6.24e-9

>gi|6323613|ref|NP_013684.1| antioxidant enzyme that provides protection against oxidation systems capable of generating reactive oxygen and sulfur species; Tsa1p [Saccharomyces cerevisiae]
MVAQVQKQAPTFKKTAVVDGVFDEVSLDKYKGKYVVLAFIPLAFTFVCPTEIIAFSEAAKKFEEQGAQVL
FASTDSEYSLLAWTNIPRKEGGLGPINIPLLADTNHSLSRDYGVLIEEEGVALRGLFIIDPKGVIRHITI
NDLPVGRNVDEALRLVEAFQWTDKNGTVLPCNWTPGAATIKPTVEDSKEYFEAANK

Modeled residue range: 3 to 195 based on template **1qmvH** (1.70 Å) Sequence Identity [%]: 66 Evalue: 1.07e-69

>gi|6324070|ref|NP_014140.1| antioxidant protein and metal homeostasis factor, protects against oxygen toxicity; Atx1p [Saccharomyces cerevisiae]
MAEIKHYQFNVVMTCSGCSGAVNKVLTKLEPDVSKIDISLEKQLVDVYTTLPYDFILEKIKKTGKEVRSG
KQL

Modeled residue range: 2 to 73 based on template **1cc7A** (1.20 Å) Sequence Identity [%]: 100 Evalue: 4.30e-32

>gi|6324090|ref|NP_014160.1| aminopeptidase of cysteine protease family; bleomycin hydrolase; Lap3p [Saccharomyces cerevisiae]
MLPTSVSRSLYLKTFRSHLLRAPQIVLKRMSSSIDISKINSWNKEFQSDLTHQLATTVLKNYNADDALLN
KTRLQKQDNRVFNTVVSTDSTPVTNQKSSGRCWLFAATNQLRLNVLSELNLKEFELSQAYLFFYDKLEKA
NYFLDQIVSSADQDIDSRLVQYLLAAPTEDGGQYSMFLNLVKKYGLIPKDLYGDLPYSTTASRKWNSLLT
TKLREFAETLRTALKERSADDSIIVTLREQMQREIFRLMSLFMDIPPVQPNEQFTWEYVDKDKKIHTIKS
TPLEFASKYAKLDPSTPVSLINDPRHPYGKLIKIDRLGNVLGGDAVIYLNVDNETLSKLVVKRLQNNKAV
FFGSHTPKFMDKKTGVMDIELWNYPAIGYNLPQQKASRIRYHESLMTHAMLITGCHVDETSKLPLRYRVE
NSWGKDSGKDGLYVMTQKYFEEYCFQIVVDINELPKELASKFTSGKEEPIVLPIWDPMGALAK

Modeled residue range: 30 to 482 based on template **2e01A** (1.73 Å) Sequence Identity [%]: 97 Evalue: 0.00e-1

>gi|6681849|ref|NP_014237.2| Homology to rat L36a and human L36a; Rpl42ap [Saccharomyces cerevisiae]
MVNVPKTRKTYCKGKTCRKHTQHKVTQYKAGKASLFAQGKRRYDRKQSGFGGQTKPVFHKKAKTTKKVVL
RLECVKCKTRAQLTLKRCKHFELGGEKKQKGQALQF

Modeled residue range: 2 to 93 based on template **2zkr4** (8.70 Å) Sequence Identity [%]: 73 Evalue: 1.84e-20

>gi|6324639|ref|NP_014708.1| Cytochrome c1; Cyt1p [Saccharomyces cerevisiae]
MFSNLSKRWAQRTLSKSFYSTATGAASKSGKLTQKLVTAGVAAAGITASTLLYADSLTAEAMTAAEHGLH
APAYAWSHNGPFETFDHASIRRGYQVYREVCAACHSLDRVAWRTLVGVSHTNEEVRNMAEEFEYDDEPDE
QGNPKKRPGKLSDYIPGPYPNEQAARAANQGALPPDLSLIVKARHGGCDYIFSLLTGYPDEPPAGVALPP
GSNYNPYFPGGSIAMARVLFDDMVEYEDGTPATTSQMAKDVTTFLNWCAEPEHDERKRLGLKTVIILSSL
YLLSIWVKKFKWAGIKTRKFVFNPPKPRK

Modeled residue range: 62 to 307 based on template **1kb9D** (2.30 Å) Sequence Identity [%]: 100 Evalue: 1.76e-145

>gi|6325168|ref|NP_015236.1| serum response factor-like protein that may function downstream of MPK1 (SLT2) MAP-kinase pathway; Rlm1p [Saccharomyces cerevisiae]
MGRRKIEIQRISDDRNRAVTFIKRKAGLFKKAHELSVLCQVDIAVIILGSNNTFYEFSSVDTNDLIYHYQ
NDKNLLHEVKDPSDYGDFHKSASVNINQDLLRSSMSNKPSKSNVKGMNQSENDDDENNDEDDDDHGNFER
NSNMHSNKKASDKNIPSAHMKLLSPTALISKMDGSEQNKRHPENALPPLQHLKRLKPDPLQISRTPQQQQ
QQNISRPYHSSMYNLNQPSSSSSSPSTMDFPKLPSFQNSSFNGRPPPISISPNKFSKPFTNASSRTPKQE
HKINNSGSNNNDNSNYTQSPSNSLEDSIQQTVKARRKLSARPVLRVRIPNNNFSSNSAIPSEPSSASSTS
ANGNSMGSSQIMKENKTSRSSKISPLSASASGPLTLQKGNNGRMVIKLPNANAPNGSNNGNGSNNNNHPY
PFGSGSSPLFSATQPYIATPLQPSNIPGGPFQQNTSFLAQRQTQQYQQMSFKKQSQTVPLTTTLTGRPPS
TFSGPETSNGPPTGSLPSKFVHDLMSNSPNVSSISMFPDWSMGPNSAKPGNTNNPGTFPPVQTAVNNGNS
SNISSTNNTNNNNNNNNNNSSNNNSNNGNDNNSNNSNNSYYSNNEDAPVNGAAISEHTTDGDSNNQSNSS
TYDAAATAYNGNTGLTPYINTAQTPLGTKFFNFSTDISGEKNSSKI

Modeled residue range: 2 to 69 based on template **1egwA** (1.50 Å) Sequence Identity [%]: 54 Evalue: 3.48e-16

>gi|6325198|ref|NP_015266.1| Member of a glutaredoxin subfamily in Sc together with GRX3 & GRX4. Significant sequence diff. with the other glutaredoxin subfamily, formed by the previously described GRX1 & GRX2 glutaredoxins (Luikenhuis MBC 9:1081, 1998); Grx5p [Saccharomyces cerevisiae]
MFLPKFNPIRSFSPILRAKTLLRYQNRMYLSTEIRKAIEDAIESAPVVLFMKGTPEFPKCGFSRATIGLL
GNQGVDPAKFAAYNVLEDPELREGIKEFSEWPTIPQLYVNKEFIGGCDVITSMARSGELADLLEEAQALV
PEEEEETKDR

Modeled residue range: 30 to 148 based on template **1ykaA** (99.9 Å) Sequence Identity [%]: 38 Evalue: 4.23e-20

```
>gi|6325296|ref|NP_015362.1| homologous to ERV1; Erv2p [Saccharomyces cerevisiae]
MKQIVKRSHAIRIVAALGIIGLWMFFSSNELSIATPGLIKAKSGIDEVQGAAAEKNDARLKEIEKQTIMP
LMGDDKVKKEVGRASWKYFHTLLARFPDEPTPEEREKLHTFIGLYAELYPCGECSYHFVKLIEKYPVQTS
SRTAAAMWGCHIHNKVNEYLKKDIYDCATILEDYDCGCSDSDGKRVSLEKEAKQHG
```

Modeled residue range: 74 to 178 based on template **1jr8A** (1.50 Å) Sequence Identity [%]: 100 Evalue: 1.01e-60

```
>gi|6325330|ref|NP_015398.1| Homologous to mammalian phosphotyrosine phosphatase; Ltp1p
[Saccharomyces cerevisiae]
MTIEKPKISVAFICLGNFCRSPMAEAIFKHEVEKANLENRFNKIDSFGTSNYHVGESPDHRTVSICKQHG
VKINHKGKQIKTKHFDEYDYIIGMDESNINNLKKIQPEGSKAKVCLFGDWNTNDGTVQTIIEDPWYGDIQ
DFEYNFKQITYFSKQFLKKEL
```

Modeled residue range: 3 to 161 based on template **1d1qA** (1.70 Å) Sequence Identity [%]: 99 Evalue: 1.04e-85

```
>gi|6319925|ref|NP_010006.1| mitochondrial thioredoxin; Trx3p [Saccharomyces cerevisiae]
MLFYKPVMRMAVRPLKSIRFQSSYTSITKLTNLTEFRNLIKQNDKLVIDFYATWCGPCKMMQPHLTKLIQ
AYPDVRFVKCDVDESPDIAKECEVTAMPTFVLGKDGQLIGKIIGANPTALEKGIKDL
```

Modeled residue range: 27 to 124 based on template **2fa4B** (2.38 Å) Sequence Identity [%]: 43 Evalue: 6.45e-23

```
>gi|6320259|ref|NP_010339.1| ubiquitin-conjugating enzyme, E2; Cdc34p [Saccharomyces cerevisiae]
MSSRKSTASSLLLRQYRELTDPKKAIPSFHIELEDDSNIFTWNIGVMVLNEDSIYHGGFFKAQMRFPEDF
PFSPPQFRFTPAIYHPNVYRDGRLCISILHQSGDPMTDEPDAETWSPVQTVESVLISIVSLLEDPNINSP
ANVDAAVDYRKNPEQYKQRVKMEVERSKQDIPKGFIMPTSESAYISQSKLDEPESNKDMADNFWYDSDLD
DDENGSVILQDDDYDDGNNHIPFEDDDVYNYNDNDDDDERIEFEDDDDDDDDSIDNDSVMDRKQPHKAED
ESEDVEDVERVSKKI
```

Modeled with HOMER.
Modeled residue range: 9 to 170 based on template 1PZV|A (2.52 Å) Sequence identity[%]: 43 PDB Blast Evalue: 2e-33

```
>gi|6320304|ref|NP_010384.1| Brain Modulosignalin Homolog; Bmh2p [Saccharomyces cerevisiae]
MSQTREDSVYLAKLAEQAERYEEMVENMKAVASSGQELSVEERNLLSVAYKNVIGARRASWRIVSSIEQK
EESKEKSEHQVELIRSYRSKIETELTKISDDILSVLDSHLIPSATTGESKVFYYKMKGDYHRYLAEFSSG
DAREKATNSSLEAYKTASEIATTELPPTHPIRLGLALNFSVFYYEIQNSPDKACHLAKQAFDDAIAELDT
LSEESYKDSTLIMQLLRDNLTLWTSDISESGQEDQQQQQQQQQQQQQQQQQAPAEQTQGEPTK
```

Modeled residue range: 5 to 234 based on template **2br9A** (1.75 Å) Sequence Identity [%]: 79 Evalue: 3.51e-90

```
>gi|6681846|ref|NP_011543.2| Protein essential for mitochondrial biogenesis and cell viability;
Erv1p [Saccharomyces cerevisiae]
MKAIDKMTDNPPQEGLSGRKIIYDEDGKPCRSCNTLLDFQYVTGKISNGLKNLSSNGKLAGTGALTGEAS
ELMPGSRTYRKVDPPDVEQLGRSSWTLLHSVAASYPAQPTDQQKGEMKQFLNIFSHIYPCNWCAKDFEKY
IRENAPQVESREELGRWMCEAHNKVNKKLRKPKFDCNFWEKRWKDGWDE
```

Modeled residue range: 84 to 189 based on template **1oqcB** (1.80 Å) Sequence Identity [%]: 47 Evalue: 5.00e-27

>gi|6321648|ref|NP_011725.1| thioredoxin; Trx2p [Saccharomyces cerevisiae]
MVTQLKSASEYDSALASGDKLVVVDFFATWCGPCKMIAPMIEKFAEQYSDAAFYKLDVDEVSDVAQKAEV
SSMPTLIFYKGGKEVTRVVGANPAAIKQAIASNV

Modeled residue range: 1 to 104 based on template **2fa4B** (2.38 Å) Sequence Identity [%]: 100 Evalue: 3.32e-48

>gi|6323072|ref|NP_013144.1| thioredoxin; Trx1p [Saccharomyces cerevisiae]
MVTQFKTASEFDSAIAQDKLVVVDFYATWCGPCKMIAPMIEKFSEQYPQADFYKLDVDELGDVAQKNEVS
AMPTLLLFKNGKEVAKVVGANPAAIKQAIAANA

Modeled residue range: 1 to 103 based on template **2i9hA** (99.9 Å) Sequence Identity [%]: 100 Evalue: 1.63e-48

>gi|6324082|ref|NP_014152.1| Protein required for cell viability; Ynl247wp [Saccharomyces cerevisiae]
MNIFIKALRRYTIMSTPKIVQPKWKVPTPQAKETVLKLYNSLTRSKVEFIPQSGNRGVTWYSCGPTVYDA
SHMGHARNYVSIDINRRIIQDYFGYDVQFVQNVTDIDDKIILRARQNYLFDNFVKENDTKFNATVVDKVK
TALFQYINKNFTIQGSEIKTIEEFETWLSNADTETLKLENPKFPMHVTAVQNAIESITKGDSMDAEVAFE
KVKDVTVPLLDKELGSTISNPEIFRQLPAYWEQKFNDDMLSLNVLPPTVTTRVSEYVPEIIDFVQKIIDN
GYAYATSDGSVYFDTLKFDKSPNHDYAKCQPWNKGQLDLINDGEGSLSNFADNGKKSNNDFALWKASKAG
EPEWESPWGKGRPGWHIECSVMASDILGSNIDIHSGGIDLAFPHHDNELAQSEARFDNQQWINYFLHTGH
LHIEGQKMSKSLKNFITIQEALKKFSPRQLRLAFASVQWNNQLDFKESLIHEVKSFENSMNNFFKTIRAL
KNDAASAGHISKKFSPLEKELLADFVESESKVHSAFCDNLSTPVALKTLSELVTKSNTYITTAGAALKIE
PLIAICSYITKILRIIGFPSRPDNLGWAAQAGSNDGSLGSLEDTVMPYVKCLSTFRDDVRSLAIKKAEPK
EFLQLTDKIRNEDLLNLNVALDDRNGQSALIKFLTNDEKLEIVKLNEEKHANELAKKQKKLEQQKLREQK
ENERKQKAQIKPQDMFKDVTLYSAWDEQGLPTKDKDGNDITKSMTKKLKKQWEQQKKLHEEYFGEDK

N-term: Modeled residue range: 35 to 133 based on template **1li5A** (2.30 Å)
Sequence Identity [%]: 43 Evalue: 1.00e-16
C-term : Modeled residue range: 221 to 602 based on template **1u0bB** (2.30 Å)
Sequence Identity [%]: 30 Evalue: 7.04e-49

>gi|6324296|ref|NP_014366.1| Synthetic interaction with Whi2; Siw14p [Saccharomyces cerevisiae]
MGLYQAKNDEGSDPKSSSKIDDLIENEAEIIRLIKEDGKLLIDNGDGRDIHNIIQEDKLLSVEFNEVLKR
FHGEEKSDIPRKEFDEDEDDGYDSNEHHQKTIEVMNTLNHVINKEVIPPENFSHVVGEIYRSSFPRQENF
SFLHERLKLKSILVLIPEEYPQENLNFLKLTGIKLYQVGMSGNKEPFVNIPSHLLTKALEIVLNPANQPI
LIHCNRGKHRTGCLIGCIRKLQNWSLTMIFDEYRRFAFPKARALDQQFIEMYDDDEIKRIASKNNWLPLQ
W

Modeled residue range: 116 to 263 based on template **1xriA** (3.30 Å) Sequence Identity [%]: 61 Evalue: 1.81e-50

>gi|6324360|ref|NP_014430.1| Similar to ubiquitin-like protein 8 of Arabidopsis thaliana and C. elegans; Hub1p [Saccharomyces cerevisiae]
MIEVVVNDRLGKKVRVKCLAEDSVGDFKKVLSLQIGTQPNKIVLQKGGSVLKDHISLEDYEVHDQTNLEL
YYL

Modeled residue range: 1 to 73 based on template **1m94A** (99.9 Å) Sequence Identity [%]: 100 Evalue: 2.44e-31

>gi|6325229|ref|NP_015297.1| acetoacetyl CoA thiolase; Erg10p [Saccharomyces cerevisiae]
MSQNVYIVSTARTPIGSFQGSLSSKTAVELGAVALKGALAKVPELDASKDFDEIIFGNVLSANLGQAPAR
QVALAAGLSNHIVASTVNKVCASAMKAIILGAQSIKCGNADVVVAGGCESMTNAPYYMPAARAGAKFGQT
VLVDGVERDGLNDAYDGLAMGVHAEKCARDWDITREQQDNFAIESYQKSQKSQKEGKFDNEIVPVTIKGF
RGKPDTQVTKDEEPARLHVEKLRSARTVFQKENGTVTAANASPINDGAAAVILVSEKVLKEKNLKPLAII
KGWGEAAHQPADFTWAPSLAVPKALKHAGIEDINSVDYFEFNEAFSVVGLVNTKILKLDPSKVNVYGGAV
ALGHPLGCSGARVVVTLLSILQQEGGKIGVAAICNGGGGASSIVIEKI

Modeled residue range: 3 to 398 based on template **2ib8B** (1.85 Å) Sequence Identity [%]: 48 Evalue: 1.97e-92

>gi|6325255|ref|NP_015323.1| appears to be functionally related to SNF7; Snf8p [Saccharomyces cerevisiae]
MKQFGLAAFDELKDGKYNDVNKTILEKQSVELRDQLMVFQERLVEFAKKHNSELQASPEFRSKFMHMCSS
IGIDPLSLFDRDKHLFTVNDFYYEVCLKVIEICRQTKDMNGGVISFQELEKVHFRKLNVGLDDLEKSIDM
LKSLECFEIFQIRGKKFLRSVPNELTSDQTKILEICSILGYSSISLLKANLGWEAVRSKSALDEMVANGL
LWIDYQGGAEALYWDPSWITRQL

Modeled residue range: 20 to 232 based on template **1w7pA** (3.60 Å) Sequence Identity [%]: 100 Evalue: 2.05e-119

>gi|6325339|ref|NP_015407.1| S. pombe dim1+ in budding yeast; Dib1p [Saccharomyces cerevisiae]
MASVLLPQLRTGWHVDQAIVTETKRLVVIRFGRKNDRQCMIMDELLSSIAERVRNFAVIYLCDIDEVSDF
DEMYELTDPMTVMFFYHNKHMMCDFGTGNNNKLNFIVDDKQEMIDILETIFRGARKNKGLVVSPYDYNHK
RVS

Modeled residue range: 5 to 138 based on template **1syxA** (2.35 Å) Sequence Identity [%]: 66 Evalue: 9.31e-49

>gi|6319498|ref|NP_009580.1| Originally identified as a multicopy suppressor of a respiratory defective mutant; homolog of Sco1p; Sco2p [Saccharomyces cerevisiae]
MLNSSRKYACRSLFRQANVSIKGLFYNGGAYRRGFSTGCCLRSDNKESPSARQPLDRLQLGDEINEPEPI
RTRFFQFSRWKATIALLLLSGGTYAYLSRKRRLLETEKEADANRAYGSVALGGPFNLTDFNGKPFTEENL
KGKFSILYFGFSHCPDICPEELDRLTYWISELDDKDHIKIQPLFISCDPARDTPDVLKEYLSDFHPAIIG
LTGTYDQVKSVCKKYKVYFSTPRDVKPNQDYLVDHSIFFYLIDPEGQFIDALGRNYDEQSGLEKIREQIQ
AYVPKEERERRSKKWYSFIFN

Modeled residue range: 120 to 299 based on template **2b7jB** (2.30 Å) Sequence Identity [%]: 68 Evalue: 1.05e-75

>gi|6319733|ref|NP_009815.1| Riboflavin biosynthesis; Rib5p [Saccharomyces cerevisiae]
MFTGIVECMGTVLENNPYDDSESGGQGVSITIGNAGSILTDCHVGDSIAVNGVCLTVTEFNNDSFKVGIS
PETIKRSNVASWIQGTQVNLERAVSQDVRFGGHYVQGHVDTVANIVSRRPEGNSIIFGFQLRDQEYFKYI
VEKGFICIDGTSLTIIKVDPLSQGGAFYISMIKHTQDNVIMPLKKIGDEVNIEVDLTGKIIEKQILLTLE
NQISKKDSTLNTMISNIIEEKVRNYLNK

Modeled residue range: 1 to 205 based on template **1kzlA** (2.10 Å) Sequence Identity [%]: 51 Evalue: 3.24e-46

>gi|6319741|ref|NP_009823.1| similar to Rab proteins and other small GTP-binding proteins; Ypt10p [Saccharomyces cerevisiae]
MIVKLLQEIQVAQNTEEYDMEATIKVVLLGDSSVGKTSIVTRLKSGKFLAKHAATIGAAFITKTIEVPSN
DSSTEKRIHMEIWDTAGQERYKSLVPMYYRDANIALIVFELGDVSSLQCAKTWFQDLQDRAQGTQVIIVG
NKYDLVCEEHSGEVTIPAELQGLPYVAVSAKTGYNFDTLNKIIISLVPESQFKTLSKNNEQGNILEINKK
KSGSGCIC

Modeled residue range: 23 to 179 based on template **1z08D** (1.80 Å) Sequence Identity [%]: 42 Evalue: 1.77e-25

>gi|6319971|ref|NP_010051.1| phosphotyrosine-specific protein phosphatase; Ptp1p [Saccharomyces cerevisiae]
MAAAPWYIRQRDTDLLGKFKFIQNQEDGRLREATNGTVNSRWSLGVSIEPRNDARNRYVNIMPYERNRVH
LKTLSGNDYINASYVKVNVPGQSIEPGYYIATQGPTRKTWDQFWQMCYHNCPLDNIVIVMVTPLVEYNRE
KCYQYWPRGGVDDTVRIASKWESPGGANDMTQFPSDLKIEFVNVHKVKDYYTVTDIKLTPTDPLVGPVKT
VHHFYFDLWKDMNKPEEVVPIMELCAHSHSLNSRGNPIIVHCSAGVGRTGTFIALDHLMHDTLDFKNITE
RSRHSDRATEEYTRDLIEQIVLQLRSQRMKMVQTKDQFLFIYHAAKYLNSLSVNQ

Modeled residue range: 47 to 324 based on template **2h4vA** (1.55 Å) Sequence Identity [%]: 31 Evalue: 4.46e-32

>gi|6320303|ref|NP_010383.1| Member of a glutaredoxin subfamily in Sc together with GRX4 & GRX5. Sign. sequence diff. with the other glutaredoxin subfamily, formed by the previously described GRX1 & GRX2 glutaredoxins (Luikenhuis MBC 9:1081, 1998); Grx3p [Saccharomyces cerevisiae]
MCSFQVPSAFSFNYTSYCYKRHQARYYTAAKLFQEMPVIEINDQEQFTYLTTTAAGDKLIVLYFHTSWAE
PCKALKQVFEAISNEPSNSNVSFLSIDADENSEISELFEISAVPYFIIIHKGTILKELSGADPKEYVSLL
EDCKNSVNSGSSQTHTMENANVNEGSHNDEDDDDEEEEEETEEQINARLTKLVNAAPVMLFMKGSPSEPK
CGFSRQLVGILREHQVRFGFFDILRDESVRQNLKKFSEWPTFPQLYINGEFQGGLDIIKESLEEDPDFLQ
HALQS

N-terminal: Modeled residue range: 36 to 135 based on template **2j23B** (1.41 Å)
Sequence Identity [%]: 37 Evalue: 1.40e-14
C-terminal: Modeled residue range: 189 to 270 based on template **1wikA** (99.9 Å) Sequence Identity [%]: 60 Evalue: 5.69e-23

>gi|6320389|ref|NP_010469.1| Phosducin-Like Protein; Plp1p [Saccharomyces cerevisiae]
MEDKLDRYYTNVLSNAEKDKHTTVDSDDKSSGEENLDELLNELDRELDEDHEFLSAYRSERLQQISDHLK
QVKKNVEDDGYGRLQCIDNEADAIQICTKTTMVVIHFELETFGKCQYMNEKLENLAKRYLTTRFIKVNVQ
TCPFLVNKLNIKVLPFVVGYKNGLEKVRYVGFSKLGNDPNGFDIRRLEQSLAHSGVIEDTFEIRKHSSVN
TERFASTNHDRSESDSDLDI

Modeled residue range: 99 to 202 based on template **2dbcA** (99.9 Å) Sequence Identity [%]: 28 Evalue: 4.97e-5

>gi|6320610|ref|NP_010690.1| Disp. for spores & spore viab. - required for dityrosine biosynth. & dityrosine accumul. in outer spore wall (s.w.); s.w. matur. & resist. to ether & lytic enz. mRNA trans. mid/late during s.w. formation; Dit2p [Saccharomyces cerevisiae]
MELLKLLCLILFLTLSYVAFAIIVPPLNFPKNIPTIPFYVVFLPVIFPIDQTELYDLYIRESMEKYGAVK
FFFGSRWNILVSRSEYLAQIFKDEDTFAKSGNQKKIPYSALAAYTGDNVISAYGAVWRNYRNAVTNGLQH
FDDAPIFKNAKILCTLIKNRLLEGQTSIPMGPLSQRMALDNISQVALGFDFGALTHEKNAFHEHLIRIKK
QIFHPFFLTFPFLDVLPIPSRKKAFKDVVSFRELLVKRVQDELVNNYKFEQTTFAASDLIRAHNNEIIDY
KQLTDNIVIILVAGHENPQLLFNSSLYLLAKYSNEWQEKLRKEVNGITDPKGLADLPLLNAFLFEVVRMY
PPLSTIINRCTTKTCKLGAEIVIPKGVYVGYNNFGTSHDPKTWGTTADDFKPERWGSDIETIRKNWRMAK
NRCAVTGFHGGRRACLGEKLALTEMRISLAEMLKQFRWSLDPEWEEKLTPAGPLCPLNLKLKFNENIME

Modeled residue range: 156 to 481 based on template **1w0gA** (2.73 Å) Sequence Identity [%]: 26 Evalue: 2.94e-15

>gi|6320695|ref|NP_010775.1| Riboflavin biosynthesis; Rib3p [Saccharomyces cerevisiae]
MFTPIDQAIEHFKQNKFVIVMDDAGRENEGDLICAAENVSTEQMAFLVRHSSGYVCAPMTNAIADKLDLP
LLRTGMKFESNDDDRHGTAYTITVDVAQGTTTGISAHDRSMTCRALADSSSTPKSFLKPGHICPLRAADG
GVLQRRGHTEAGVDLCKLSGLSPVAVIGELVNDDEQGTMMRLNDCQAFGKKHGIPLISIEELAQYLKK

Modeled residue range: 1 to 208 based on template **1tksB** (1.60 Å) Sequence Identity [%]: 57 Evalue: 5.48e-63

```
>gi|6320726|ref|NP_010806.1| ER protein functionally likely involved in interacting with nascent polypeptides in the ER; Eug1p [Saccharomyces cerevisiae]
MQVTTRFISAIVSFCLFASFTLAENSARATPGSDLLVLTEKKFKSFIESHPLVLVEFFAPWCLHSQILRP
HLEEAASILKEHNVPVVQIDCEANSMVCLQQTINTYPTLKIFKNGRIFDGQVYRGVKITDEITQYMIQLY
EASVIYLNSEDEIQPYLENATLPVVINRGLTGLNETYQEVALDLAEDYVFLSLLDSEDKSLSIHLPNTTE
PILFDGNVDSLVGNSVALTQWLKVVILPYFTDIEPDLFPKYISSNLPLAYFFYTSEEELEDYTDLFTQLG
KENRGQINFIALNSTMFPHHVRFLNMREQFPLFAIHNMINNLKYGLPQLPEEEYAKLEKPQPLDRDMIVQ
LVKDYREGTAKPIVKSEEIPKEQKSNVYKIVGKTHDDIVHDDDKDVLVKYYATWCIHSKRFAPIYEEIAN
VLASDESVRDKILIAEVDSGANDILSFPVTGYPTIALYPAGNNSKPIIFNKIRNLEDVFEFIKESGTHHI
DGQAIYDKLHQAKDSEVSTEDTVHDEL
```

Modeled residue range: 24 to 496 based on template **2b5eA** (2.40 Å) Sequence Identity [%]: 42 Evalue: 6.09e-105

```
>gi|6320808|ref|NP_010887.1| vacuolar ATPase V0 domain subunit c (17 kDa); Cup5p [Saccharomyces cerevisiae]
MTELCPVYAPFFGAIGCASAIIFTSLGAAYGTAKSGVGICATCVLRPDLLFKNIVPVIMAGIIAIYGLVV
SVLVCYSLGQKQALYTGFIQLGAGLSVGLSGLAAGFAIGIVGDAGVRGSSQQPRLFVGMILILIFAEVLG
LYGLIVALLLNSRATQDVVC
```

Modeled residue range: 12 to 150 based on template **2bl2B** (2.10 Å) Sequence Identity [%]: 27 Evalue: 9.13e-9

```
>gi|6320869|ref|NP_010948.1| probably involved in intra-Golgi transport or in the formation of transport vesicles at the most distal Golgi compartment; Ypt31p [Saccharomyces cerevisiae]
MSSEDYGYDYDLLFKIVLIGDSGVGKSNLLSRFTKNEFNMDSKSTIGVEFATRTLEIDGKRIKAQIWDTA
GQERYRAITSAYYRGAVGALIVYDISKSSSYENCNHWLSELRENADDNVAVGLIGNKSDLAHLRAVPTEE
SKTFAQENQLLFTETSALNSENVDKAFEELINTIYQKVSKHQMDLGDSSANGNANGASAPNGPTISLTPT
PNENKKANGNNCC
```

Modeled residue range: 10 to 183 based on template **2f9mA** (1.95 Å) Sequence Identity [%]: 70 Evalue: 5.45e-67

```
>gi|6320881|ref|NP_010960.1| peptide Methionine sulfoXide Reductase 1; Mxr1p [Saccharomyces cerevisiae]
MSSLISKTIKYDPAKDKLITLACGCFWGTEHMYRKYLNDRIVDCKVGYANGEESKKDSPSSVSYKRVCGG
DTDFAEVLQVSYNPKVITLRELTDFFFRIHDPTTSNSQGPDKGTQYRSGLFAHSDADLKELAKIKEEWQP
KWGNKIATVIEPIKNFYDAEEYHQLYLDKNPQGYACPTHYLREM
```

Modeled residue range: 6 to 174 based on template **1fvaB** (1.70 Å) Sequence Identity [%]: 36 Evalue: 4.50e-23

```
>gi|6320917|ref|NP_010996.1| Utilizes NADP+ as the preferred coenzyme. Activated by K+.; Ald5p [Saccharomyces cerevisiae]
MLSRTRAAAPNSRIFTRSLLRLYSQAPLRVPITLPNGFTYEQPTGLFINGEFVASKQKKTFDVINPSNEE
KITTVYKAMEDDVDEAVAAAKKAFETKWSIVEPEVRAKALFNLADLVEKHQETLAAIESMDNGKSLFCAR
GDVALVSKYLRSCGGWADKIYGNVIDTGKNHFTYSIKEPLGVCGQIIPWNFPLLMWSWKIGPALATGNTV
VLKPAETTPLSALFASQLCQEAGIPAGVVNILPGSGRVVGERLSAHPDVKKIAFTGSTATGRHIMKVAAD
TVKKVTLELGGKSPNIVFADADLDKAVKNIAFGIFYNSGEVCCAGSRIYIQDTVYEEVLQKLKDYTESLK
VGDPFDEEVFQGAQTSDKQLHKILDYVDVAKSEGARLVTGGARHGSKGYFVKPTVFADVKGDMRIVKEEV
FGPIVTVSKFSTVDEVIAMANDSQYGLAAGIHTNDINKAVDVSKRVKAGTVWINTYNNFHQNVPFGGFGQ
SGIGREMGEAALSNYTQTKSVRIAIDKPIR
```

Modeled residue range: 46 to 515 based on template **1o04G** (1.42 Å) Sequence Identity [%]: 48 Evalue: 1.73e-131

```
>gi|6320936|ref|NP_011015.1| vitamin B12-(cobalamin)-independent isozyme of methionine synthase (also called N5-methyltetrahydrofolate homocysteine methyltransferase or 5-methyltetrahydropteroyl triglutamate homocysteine methyltransferase); Met6p [Saccharomyces cerevisiae]
MVQSAVLGFPRIGPNRELKKATEGYWNGKITVDELFKVGKDLRTQNWKLQKEAGVDIIPSNDFSFYDQVL
DLSLLFNVIPDRYTKYDLSPIDTLFAMGRGLQRKATETEKAVDVTALEMVKWFDSNYHYVRPTFSKTTQF
KLNGQKPVDEFLEAKELGIHTRPVLLGPVSYLFLGKADKDSLDLEPLSLLEQLLPLYTEILSKLASAGAT
EVQIDEPVLVLDLPANAQAAIKKAYTYFGEQSNLPKITLATYFGTVVPNLDAIKGLPVAALHVDFVRAPE
QFDEVVAAIGNKQTLSVGIVDGRNIWKNDFKKSSAIVNKAIEKLGADRVVVATSSSLLHTPVDLNNETKL
DAEIKGFFSFATQKLDEVVVITKNVSGQDVAAALEANAKSVESRGKSKFIHDAAVKARVASIDEKMSTRA
APFEQRLPEQQKVFNLPLFPTTTIGSFPQTKDIRINRNKFNKGTISAEEYEKFINSEIEKVIRFQEEIGL
DVLVHGEPERNDMVQYFGEQINGYAFTVNGWVQSYGSRYVRPPIIVGDLSRPKAMSVKESVYAQSITSKP
VKGMLTGPITCLRWSFPRDDVDQKTQAMQLALALRDEVNDLEAAGIKVIQVDEPALREGLPLREGTERSA
YYTWAAEAFRVATSGVANKTQIHSHFCYSDLDPNHIKALDADVVSIEFSKKDDANYIAEFKNYPNHIGLG
LFDIHSPRIPSKDEFIAKISTILKSYPAEKFWVNPDCGLKTRGWEETRLSLTHMVEAAKYFREQYKN
```

Modeled residue range: 4 to 764 based on template **1u1jA** (2.40 Å) Sequence Identity [%]: 47 Evalue: 0.00e-1

```
>gi|6320972|ref|NP_011051.1| involved in ubiquitin-mediated protein degradation; Rsp5p [Saccharomyces cerevisiae]
MPSSISVKLVAAESLYKRDVFRSPDPFAVLTIDGYQTKSTSAAKKTLNPYWNETFKFDDINENSILTIQV
FDQKKFKKKDQGFLGVVNVRVGDVLGHLDEDTATSSGRPREETITRDLKKSNDGMAVSGRLIVVLSKLPS
SSPHSQAPSGHTASSSTNTSSTTRTNGHSTSSTRNHSTSHPSRGTAQAVESTLQSGTTAATNTATTSHRS
TNSTSSATRQYSSFEDQYGRLPPGWERRTDNFGRTYYVDHNTRTTTWKRPTLDQTEAERGNQLNANTELE
RRQHRGRTLPGGSSDNSSVTVQVGGGSNIPPVNGAAAAAFAATGGTTSGLGELPSGWEQRFTPEGRAYFV
DHNTRTTTWVDPRRQQYIRTYGPTNTTIQQQPVSQLGPLPSGWEMRLTNTARVYFVDHNTKTTTWDDPRL
PSSLDQNVPQYKRDFRRKVIYFRSQPALRILPGQCHIKVRRKNIFEDAYQEIMRQTPEDLKKRLMIKFDG
EEGLDYGGVSREFFFLLSHEMFNPFYCLFEYSAYDNYTIQINPNSGINPEHLNYFKFIGRVVGLGVFHRR
FLDAFFVGALYKMMLRKKVVLQDMEGVDAEVYNSLNWMLENSIDGVLDLTFSADDERFGEVVTVDLKPDG
RNIEVTDGNKKEYVELYTQWRIVDRVQEQFKAFMDGFNELIPEDLVTVFDERELELLIGGIAEIDIEDWK
KHTDYRGYQESDEVIQWFWKCVSEWDNEQRARLLQFTTGTSRIPVNGFKDLQGSDGPRRFTIEKAGEVQQ
LPKSHTCFNRVDLPQYVDYDSMKQKLTLAVEETIGFGQE
```

Modeled residue range: 430 to 801 based on template **2oniA** (2.20 Å) Sequence Identity [%]: 56 Evalue: 5.39e-123

```
>gi|6321025|ref|NP_011104.1| Brain Modulosignalin Homolog; Bmh1p [Saccharomyces cerevisiae]
MSTSREDSVYLAKLAEQAERYEEMVENMKTVASSGQELSVEERNLLSVAYKNVIGARRASWRIVSSIEQK
EESKEKSEHQVELICSYRSKIETELTKISDDILSVLDSHLIPSATTGESKVFYYKMKGDYHRYLAEFSSG
DAREKATNASLEAYKTASEIATTELPPTHPIRLGLALNFSVFYYEIQNSPDKACHLAKQAFDDAIAELDT
LSEESYKDSTLIMQLLRDNLTLWTSDMSESGQAEDQQQQQQHQQQQPPAAAEGEAPK
```

Modeled residue range: 5 to 234 based on template **2br9A** (1.75 Å) Sequence Identity [%]: 78 Evalue: 1.59e-89

```
>gi|14318459|ref|NP_116595.1| SNZ3 proximal ORF, stationary phase induced gene family; Sno3p [Saccharomyces cerevisiae]
MTVVIGVLALQGAFIEHVRHVEKCIVENRDFYEKKLSVMTVKDKNQLAQCDALIIPGGESTAMSLIAERT
GFYDDLYAFVHNPSKVTWGTCAGLIYISQQLSNEAKLVKTLNLLKVKVKRNAFGRQAQSSTRICDFSNFI
PHCNDFPATFIRAPVIEEVLDPEHVQVLYKLDGKDNGGQELIVAAKQKNNILATSFHPELAENDIRFHDW
FIREFVLKNYSK
```

Modeled residue range: 3 to 220 based on template **2abwB** (1.62 Å) Sequence Identity [%]: 35 Evalue: 1.01e-23

>gi|14318463|ref|NP_116599.1| high degree of similarity with the AAD of P. chrysosporium; Aad6p [Saccharomyces cerevisiae]
MADLFAPAPEPSTELGRLRVLSKSAGIRVSPLILGGMSIGDAWSEILGSMSKERAFELLDAFYEAGGNFI
DTANNYQNEQSEAWIGEWMVSRKLRDQIVIATKFTTDYKKYDVGGGKSANYCGNHKRSLHVSVRDSLRKL
QTDWIDILYVHWWDYMSSIEEVMDSLHILVQQARSSIWVCLIRLPGLFLRQITTLNLMVKPLLASIKVNG
TC

Modeled residue range: 26 to 166 based on template **1pz1A** (2.20 Å) Sequence Identity [%]: 32 Evalue: 1.32e-11

>gi|14318479|ref|NP_116614.1| Involved in cell polarization, endocytosis and other cytoskeletal functions; Act1p [Saccharomyces cerevisiae]
MDSEVAALVIDNGSGMCKAGFAGDDAPRAVFPSIVGRPRHQGIMVGMGQKDSYVGDEAQSKRGILTLRYP
IEHGIVTNWDDMEKIWHHTFYNELRVAPEEHPVLLTEAPMNPKSNREKMTQIMFETFNVPAFYVSIQAVL
SLYSSGRTTGIVLDSGDGVTHVVPIYAGFSLPHAILRIDLAGRDLTDYLMKILSERGYSFSTTAEREIVR
DIKEKLCYVALDFEQEMQTAAQSSSIEKSYELPDGQVITIGNERFRAPEALFHPSVLGLESAGIDQTTYN
SIMKCDVDVRKELYGNIVMSGGTTMFPGIAERMQKEITALAPSSMKVKIIAPPERKYSVWIGGSILASLT
TFQQMWISKQEYDESGPSIVHHKCF

Modeled residue range: 4 to 371 based on template **2fxuA** (1.35 Å) Sequence Identity [%]: 85 Evalue: 0.00e-1

>gi|14318481|ref|NP_116616.1| beta subunit of tubulin monomer; involved in chromosome segregation and nuclear migration; Tub2p [Saccharomyces cerevisiae]
MREIIHISTGQCGNQIGAAFWETICGEHGLDFNGTYHGHDDIQKERLNVYFNEASSGKWVPRSINVDLEP
GTIDAVRNSAIGNLFRPDNYIFGQSSAGNVWAKGHYTEGAELVDSVMDVIRREAEGCDSLQGFQITHSLG
GGTGSGMGTLLISKIREEFPDRMMATFSVLPSPKTSDTVVEPYNATLSVHQLVEHSDETFCIDNEALYDI
CQRTLKLNQPSYGDLNNLVSSVMSGVTTSLRYPGQLNSDLRKLAVNLVPFPRLHFFMVGYAPLTAIGSQS
FRSLTVPELTQQMFDAKNMMAAADPRNGRYLTVAAFFRGKVSVKEVEDEMHKVQSKNSDYFVEWIPNNVQ
TAVCSVAPQGLDMAATFIANSTSIQELFKRVGDQFSAMFKRKAFLHWYTSEGMDELEFSEAESNMNDLVS
EYQQYQEATVEDDEEVDENGDFGAPQNQDEPITENFE

Modeled residue range: 2 to 427 based on template **1tvkB** (2.89 Å) Sequence Identity [%]: 77 Evalue: 0.00e-1

>gi|14318551|ref|NP_116684.1| Required for mitosis and sporulation; Cdc14p [Saccharomyces cerevisiae]
MRRSVYLDNTIEFLRGRVYLGAYDYTPEDTDELVFFTVEDAIFYNSFHLDFGPMNIGHLYRFAVIFHEIL
NDPENANKAVVFYSSASTRQRANAACMLCCYMILVQAWTPHQVLQPLAQVDPPFMPFRDAGYSNADFEIT
IQDVVYGVWRAKEKGLIDLHSFNLESYEKYEHVEFGDFNVLTPDFIAFASPQEDHPKGYLATKSSHLNQP
FKSVLNFFANNNVQLVVRLNSHLYNKKHFEDIGIQHLDLIFEDGTCPDLSIVKNFVGAAETIIKRGGKIA
VHCKAGLGRTGCLIGAHLIYTYGFTANECIGFLRFIRPGMVVGPQQHWLYLHQNDFREWKYTTRISLKPS
EAIGGLYPLISLEEYRLQKKKLKDDKRVAQNNIEGELRDLTMTPPSNGHGALSARNSSQPSTANNGSNSF
KSSAVPQTSPGQPRKGQNGSNTIEDINNNRNPTSHANRKVVIESNNSDDESMQDTNGTSNHYPKVSRKKN
DISSASSSRMEDNEPSATNINNAADDTILRQLLPKNRRVTSGRRTTSAAGGIRKISGSIKK

Modeled residue range: 5 to 336 based on template **1ohcA** (2.50 Å) Sequence Identity [%]: 37 Evalue: 1.46e-61

>gi|14318480|ref|NP_116615.1| involved in the secretion pathway at the ER-to-Golgi step; required for sporulation; Ypt1p [Saccharomyces cerevisiae]
MNSEYDYLFKLLLIGNSGVGKSCLLLRFSDDTYTNDYISTIGVDFKIKTVELDGKTVKLQIWDTAGQERF
RTITSSYYRGSHGIIIVYDVTDQESFNGVKMWLQEIDRYATSTVLKLLVGNKCDLKDKRVVEYDVAKEFA
DANKMPFLETSALDSTNVEDAFLTMARQIKESMSQQNLNETTQKKEDKGNVNLKGQSLTNTGGGCC

Modeled residue range: 3 to 205 based on template **1ukvY** (1.50 Å) Sequence Identity [%]: 100 Evalue: 1.12e-102

>gi|6321228|ref|NP_011305.1| probably involved in intra-Golgi transport or in the formation of transport vesicles at the most distal Golgi compartment; Ypt32p [Saccharomyces cerevisiae]
MSNEDYGYDYDYLFKIVLIGDSGVGKSNLLSRFTTDEFNIESKSTIGVEFATRTIEVENKKIKAQIWDTA
GQERYRAITSAYYRGAVGALIVYDISKSSSYENCNHWLTELRENADDNVAVGLIGNKSDLAHLRAVPTDE
AKNFAMENQMLFTETSALNSDNVDKAFRELIVAIFQMVSKHQVDLSGSGTNNMGSNGAPKGPTISLTPAP
KEDKKKKSSNCC

Modeled residue range: 10 to 183 based on template **2f9mA** (1.95 Å) Sequence Identity [%]: 70 Evalue: 3.68e-67

>gi|6321643|ref|NP_011720.1| Required for the biosynthesis of purines, thymidylate, methionine, histidine, pantothenic acid and formylmethionyl-tRNA; Ade3p [Saccharomyces cerevisiae]
MAGQVLDGKACAQQFRSNIANEIKSIQGHVPGFAPNLAIIQVGNRPDSATYVRMKRKAAEEAGIVANFIH
LDESATEFEVLRYVDQLNEDPHTHGIIVQLPLPAHLDEDRITSRVLAEKDVDGFGPTNIGELNKKNGHPF
FLPCTPKGIIELLHKANVTIEGSRSVVIGRSDIVGSPVAELLKSLNSTVTITHSKTRDIASYLHDADIVV
VAIGQPEFVKGEWFKPRDGTSSDKKTVVIDVGTNYVADPSKKSGFKCVGDVEFNEAIKYVHLITPVPGGV
GPMTVAMLMQNTLIAAKRQMEESSKPLQIPPLPLKLLTPVPSDIDISRAQQPKLINQLAQELGIYSHELE
LYGHYKAKISPKVIERLQTRQNGKYILVSGITPTPLGEGKSTTTMGLVQALTAHLGKPAIANVRQPSLGP
TLGVKGGAAGGGYSQVIPMDEFNLHLTGDIHAIGAANNLLAAAIDTRMFHETTQKNDATFYNRLVPRKNG
KRKFTPSMQRRLNRLGIQKTNPDDLTPEEINKFARLNIDPDTITIKRVVDINDRMLRQITIGQAPTEKNH
TRVTGFDITVASELMAILALSKDLRDMKERIGRVVVAADVNRSPVTVEDVGCTGALTALLRDAIKPNLMQ
TLEGTPVLVHAGPFANISIGASSVIADRVALKLVGTEPEAKTEAGYVVTEAGFDFTMGGERFFNIKCRSS
GLTPNAVVLVATVRALKSHGGAPDVKPGQPLPSAYTEENIEFVEKGAANMCKQIANIKQFGVPVVVAINK
FETDTEGEIAAIRKAALEAGAFEAVTSNHWAEGGKGAIDLAKAVIEASNQPVDFHFLYDVNSSVEDKLTT
IVQKMYGGAAIDILPEAQRKIDMYKEQGFGNLPICIAKTQYSLSHDATLKGVPTGFTFPIRDVRLSNGAG
YLYALAAEIQTIPGLATYAGYMAVEVDDDGEIDGLF

Modeled residue range: 323 to 944 based on template **1fpmA** (3.00 Å) Sequence Identity [%]: 45 Evalue: 1.32e-137

>gi|6321691|ref|NP_011768.1| functions in the Ada and SAGA (Spt/Ada) complexes to acetylate nucleosomal histones; Gcn5p [Saccharomyces cerevisiae]
MVTKHQIEEDHLDGATTDPEVKRVKLENNVEEIQPEQAETNKQEGTDKENKGKFEKETERIGGSEVVTDV
EKGIVKFEFDGVEYTFKERPSVVEENEGKIEFRVVNNDNTKENMMVLTGLKNIFQKQLPKMPKEYIARLV
YDRSHLSMAVIRKPLTVVGGITYRPFDKREFAEIVFCAISSTEQVRGYGAHLMNHLKDYVRNTSNIKYFL
TYADNYAIGYFKKQGFTKEITLDKSIWMGYIKDYEGGTLMQCSMLPRIRYLDAGKILLLQEAALRRKIRT
ISKSHIVRPGLEQFKDLNNIKPIDPMTIPGLKEAGWTPEMDALAQRPKRGPHDAAIQNILTELQNHAAAW
PFLQPVNKEEVPDYYDFIKEPMDLSTMEIKLESNKYQKMEDFIYDARLVFNNCRMYNGENTSYYKYANRL
EKFFNNKVKEIPEYSHLID

Modeled residue range: 99 to 262 based on template **1yghA** (1.90 Å) Sequence Identity [%]: 100 Evalue: 6.64e-93

>gi|6321832|ref|NP_011908.1| NADP-cytochrome P450 reductase; Ncp1p [Saccharomyces cerevisiae]
MPFGIDNTDFTVLAGLVLAVLLYVKRNSIKELLMSDDGDITAVSSGNRDIAQVVTENNKNYLVLYASQTG
TAEDYAKKFSKELVAKFNLNVMCADVENYDFESLNDVPVIVSIFISTYGEGDFPDGAVNFEDFICNAEAG
ALSNLRYNMFGLGNSTYEFFNGAAKKAEKHLSAAGAIRLGKLGEADDGAGTTDEDYMAWKDSILEVLKDE
LHLDEQEAKFTSQFQYTVLNEITDSMSLGEPSAHYLPSHQLNRNADGIQLGPFDLSQPYIAPIVKSRELF
SSNDRNCIHSEFDLSGSNIKYSTGDHLAVWPSNPLEKVEQFLSIFNLDPETIFDLKPLDPTVKVPFPTPT
TIGAAIKHYLEITGPVSRQLFSSLIQFAPNADVKEKLTLLSKDKDQFAVEITSKYFNIADALKYLSDGAK
WDTVPMQFLVESVPQMTPRYYSISSSSLSEKQTVHVTSIVENFPNPELPDAPPVVGVTTNLLRNIQLAQN
NVNIAETNLPVHYDLNGPRKLFANYKLPVHVRRSNFRLPSNPSTPVIMIGPGTGVAPFRGFIRERVAFLE
SQKKGGNNVSLGKHILFYGSRNTDDFLYQDEWPEYAKKLDGSFEMVVAHSRLPNTKKVYVQDKLKDYEDQ

VFEMINNGAFIYVCGDAKGMAKGVSTALVGILSRGKSITTDEATELIKMLKTSGRYQEDVW

Modeled residue range: 47 to 691 based on template **2bpoA** (2.90 Å) Sequence Identity [%]: 99 Evalue: 0.00e-1

>gi|6322027|ref|NP_012102.1| nitrilase; Nit1p [Saccharomyces cerevisiae]
MAKHIVAALQIGSCPGSTKDTLKKILSYEKEIKESGAKLVVIPEATLGGYPKGSNFGVYLGYRLQEGREE
YAKYLAEAIEIGNGEKYPEISQLCALSKATDASLCVGCIERDGTTLYCTMVYIDPKDGYVGKHRKLMPTA
GERLIWGQGDGSTLPVVDTAAGKIGGAICWENMMPLLRYAMYKKGVEIWCAPTVDARPI

Modeled residue range: 30 to 192 based on template **1j31C** (1.60 Å) Sequence Identity [%]: 32 Evalue: 3.29e-9

>gi|6323822|ref|NP_013893.1| Expression induced in response to high osmotic stress. NAD+ is preferred coenzyme.; Ald2p [Saccharomyces cerevisiae]
MPTLYTDIEIPQLKISLKQPLGLFINNEFCPSSDGKTIETVNPATGEPITSFQAANEKDVDKAVKAARAA
FDNVWSKTSSEQRGIYLSNLLKLIEEEQDTLAALETLDAGKPYHSNAKGDLAQILQLTRYFAGSADKFDK
GATIPLTFNKFAYTLKVPFGVVAQIVPWNYPLAMACWKLQGALAAGNTVIIKPAENTSLSLLYFATLIKK
AGFPPGVVNIVPGYGSLVGQALASHMDIDKISFTGSTKVGGFVLEASGQSNLKDVTLECGGKSPALVFED
ADLDKAIDWIAAGIFYNSGQNCTANSRVYVQSSIYDKFVEKFKETAKKEWDVAGKFDPFDEKCIVGPVIS
STQYDRIKSYIERGKREEKLDMFQTSEFPIGGAKGYFIPPTIFTDVPQTSKLLQDEIFGPVVVVSKFTNY
DDALKLANDTCYGLASAVFTKDVKKAHMFARDIKAGTVWINSSNDEDVTVPFGGFKMSGIGRELGQSGVD
TYLQTKAVHINLSLDN

Modeled residue range: 23 to 506 based on template **1o04G** (1.42 Å) Sequence Identity [%]: 48 Evalue: 1.79e-122

>gi|6681848|ref|NP_012160.2| Mps One Binder; Mob1p [Saccharomyces cerevisiae]
MSFLQNFHISPGQTIRSTRGFKWNTANAANNAGSVSPTKATPHNNTINGNNNNANTINNRADFTNNPVNG
YNESDHGRMSPVLTTPKRHAPPPEQLQNVTDFNYTPSHQKPFLQPQAGTTVTTHQDIKQIVEMTLGSEGV
LNQAVKLPRGEDENEWLAVHCVDFYNQINMLYGSITEFCSPQTCPRMIATNEYEYLWAFQKGQPPVSVSA
PKYVECLMRWCQDQFDDESLFPSKVTGTFPEGFIQRVIQPILRRLFRVYAHIYCHHFNEILELNLQTVLN
TSFRHFCLFAQEFELLRPADFGPLLELVMELRDR

Modeled residue range: 81 to 313 based on template **2hjnA** (2.00 Å) Sequence Identity [%]: 88 Evalue: 7.94e-116

>gi|6322155|ref|NP_012230.1| capping - addition of actin subunits; Cap2p [Saccharomyces cerevisiae]
MSDAQFDAALDLLRRLNPTTLQENLNNLIELQPNLAQDLLSSVDVPLSTQKDSADSNREYLCCDYNRDID
SFRSPWSNTYYPELSPKDLQDSPFPSAPLRKLEILANDSFDVYRDLYYEGGISSVYLWDLNEEDFNGHDF
AGVVLFKKNQSDHSNWDSIHVFEVTTSPSSPDSFNYRVTTTIILHLDKTKTDQNSHMMLSGNLTRQTEKD
IAIDMSRPLDVIFTSHVANLGSLIEDIESQMRNLLETVYFEKTRDIFHQTKNAAIASSAEEANKDAQAEV
IRGLQSL

Modeled residue range: 2 to 256 based on template **1iznD** (2.10 Å) Sequence Identity [%]: 50 Evalue: 8.43e-60

>gi|6322811|ref|NP_012884.1| Nifu-like protein; Nfu1p [Saccharomyces cerevisiae]
MFKSVAKLGKSPIFYLNSQRLIHIKTLTTPNENALKFLSTDGEMLQTRGSKSIVIKNTDENLINHSKLAQ
QIFLQCPGVESLMIGDDFLTINKDRMVHWNSIKPEIIDLLTKQLAYGEDVISKEFHAVQEEEGEGGYKIN
MPKFELTEEDEEVSELIEELIDTRIRPAILEDGGDIDYRGWDPKTGTVYLRLQGACTSCSSSEVTLKYGI
ESMLKHYVDEVKEVIQIMDPEQEIALKEFDKLEKKLESSKNTSHEK

Modeled residue range: 153 to 230 based on template **1vehA** (99.9 Å) Sequence Identity [%]: 55 Evalue: 7.96e-18

>gi|6323244|ref|NP_013316.1| Product of gene unknown; Cdc123p [Saccharomyces cerevisiae]

MSSQEYTTFIDIPVTRAQVEHCSYSFWSSLYPKYVPKSIVLKSLPKKFIQYLEQDGIKLPQEENSRSVYT
EEIIRNEDNDYSDWEDDEDTATEFVQEVEPLIDFPELHQKLKDALNELGAVAPKLNWSAPRDATWILPNN
TMKCNEVNELYLLLNASNYIMHDLQRAFKGCVDGDDIKGLKFDLVLRQWCDMNPALEFRVFVKNAHIVGA
TQRDLNYYDYLDELSDTFKDLIDEIVHDVVLPKFPDKSFVLDVYIPRPFNKIFIVDINPFARKTDSLLFS
WNEIAAIAPPKNDVEDYELRLVTRHNTGRFASKEHSENHVPQDLVEASLNPEAIRELTQKWKELLSQQAK
EESSDSENET

Modeled with Homer
Modeled residue range: 79 to 229 based on template **2potA** (1.80 Å) Sequence Identity [%]: 25 PDB Blast Evalue: 2.2

>gi|6322908|ref|NP_012981.1| ras homolog--GTP binding protein; Rho4p [Saccharomyces cerevisiae]
MNTLLFKRKGGNCGNESNIVSQGSPSSSNLPESPGTLDEKNLPRLPTPFARSLSTIPSYEQMKRTNKLPD
YHLKIVVVGDGAVGKTCLLISYVQGTFPTDYIPTIFENYVTNIEGPNGQIIELALWDTAGQEEYSRLRPL
SYTNADVLMVCYSVGSKTSLKNVEDLWFPEVKHFCPSTPIMLVGLKSDLYEADNLSDLVEPSSAESLAKR
LGAFAHIQCSARLKENIDEVFETAIHTLLSDSLYAPREPTHTIKNPFKRNTTRSDIDSSTGDTSVSISGT
KRLRKNKCIIM

Modeled residue range: 74 to 239 based on template **1s1cB** (2.60 Å) Sequence Identity [%]: 51 Evalue: 3.10e-48

>gi|6322921|ref|NP_012994.1| Hydrophilic protein that acts in conjunction with SNARE proteins in targeting and fusion of ER to Golgi transport vesicles; Bet3p [Saccharomyces cerevisiae]
MVSTTQSRSLKAMGEEIWKNKTEKINTELFTLTYGSIVAQLCQDYERDFNKVNDHLYSMGYNIGCRLIED
FLARTALPRCENLVKTSEVLSKCAFKIFLNITPNITNWSHNKDTFSLILDENPLADFVELPMDAMKSLWY
SNILCGVLKGSLEMVQLDCDVWFVSDILRGDSQTEIKVKLNRILKDEIPIGED

Modeled residue range: 23 to 182 based on template **1wc9A** (1.60 Å) Sequence Identity [%]: 58 Evalue: 9.36e-49

>gi|6323138|ref|NP_013210.1| alkyl hydroperoxide reductase; Ahp1p [Saccharomyces cerevisiae]
MSDLVNKKFPAGDYKFQYIAISQSDADSESCKMPQTVEWSKLISENKKVIITGAPAAFSPTCTVSHIPGY
INYLDELVKEKEVDQVIVVTVDNPFANQAWAKSLGVKDTTHIKFASDPGCAFTKSIGFELAVGDGVYWSG
RWAMVVENGIVTYAAKETNPGTDVTVSSVESVLAHL

Modeled residue range: 47 to 176 based on template **1tp9A** (1.62 Å) Sequence Identity [%]: 40 Evalue: 3.45e-18

>gi|6323224|ref|NP_013296.1| N-myristoyl transferase; Nmt1p [Saccharomyces cerevisiae]
MSEEDKAKKLENLLKLLQLNNDDTSKFTQEQKKAMKDHKFWRTQPVKDFDEKVVEEGPIDKPKTPEDISD
KPLPLLSSFEWCSIDVDNKKQLEDVFVLLNENYVEDRDAGFRFNYTKEFFNWALKSPGWKKDWHIGVRVK
ETQKLVAFISAIPVTLGVRGKQVPSVEINFLCVHKQLRSKRLTPVLIKEITRRVNKCDIWHALYTAGIVL
PAPVSTCRYTHRPLNWKKLYEVDFTGLPDGHTEEDMIAENALPAKTKTAGLRKLKKEDIDQVFELFKRYQ
SRFELIQIFTKEEFEHNFIGEESLPLDKQVIFSYVVEQPDGKITDFFSFYSLPFTILNNTKYKDLGIGYL
YYYATDADFQFKDRFDPKATKALKTRLCELIYDACILAKNANMDVFNALTSQDNTLFLDDLKFGPGDGFL
NFYLFNYRAKPITGGLNPDNSNDIKRRSNVGVVML

Modeled residue range: 34 to 455 based on template **1iicB** (2.20 Å) Sequence Identity [%]: 100 Evalue: 0.00e-1

>gi|6323383|ref|NP_013455.1| Nit protein, nitrilase superfamily member; Nit3p [Saccharomyces cerevisiae]
MSASKILSQKIKVALVQLSGSSPDKMANLQRAATFIERAMKEQPDTKLVVLPECFNSPYSTDQFRKYSEV
INPKEPSTSVQFLSNLANKFKIILVGGTIPELDPKTDKIYNTSIIFNEDGKLIDKHRKVHLFDVDIPNGI
SFHESETLSPGEKSTTIDTKYGKFGVGICYDMRFPELAMLSARKGAFAMIYPSAFNTVTGPLHWHLLARS

RAVDNQVYVMLCSPARNLQSSYHAYGHSIVVDPRGKIVAEAGEGEEIIYAELDPEVIESFRQAVPLTKQR
RFDVYSDVNAH

Modeled residue range: 2 to 282 based on template **1f89A** (2.40 Å) Sequence Identity [%]: 96 Evalue: 6.86e-154

>gi|6323609|ref|NP_013680.1| Required for X-ray damage repair & various types of intra- and interchr. mitotic recombination, including HO switching & plasmid exchange. Disp. for premeiotic DNA synthesis, double strand breaks & heteroduplex formation.; Rad52p [Saccharomyces cerevisiae]
MAFLSYFATENQQMQTRRLPRTAEGSGGFGVLLMNEIMDMDEKKPVFGNHSEDIQTKLDKKLGPEYISKR
VGFGTSRIAYIEGWRVINLANQIFGYNGWSTEVKSVVIDFLDERQGKFSIGCTAIVRVTLTSGTYREDIG
YGTVENERRKPAAFERAKKSAVTDALKRSLRGFGNALGNCLYDKDFLAKIDKVKFDPPDFDENNLFRPTD
EISESSRTNTLHENQEQQQYPNKRRQLTKVTNTNPDSTKNLVKIENTVSRGTPMMAAPAEANSKNSSNKD
TDLKSLDASKQDQDDLLDDSLMFSDDFQDDDLINMGNTNSNVLTTEKDPVVAKQSPTASSNPEAEQITFV
TAKAATSVQNERYIGEESIFDPKYQAQSIRHTVDQTTSKHIPASVLKDKTMTTARDSVYEKFAPKGKQLS
MKNNDKELGPHMLEGAGNQVPRETTPIKTNATAFPPAAAPRFAPPSKVVHPNGNGAVPAVPQQRSTRREV
GRPKINPLHARKPT

Modeled residue range: 52 to 198 based on template **1h2iP** (2.70 Å) Sequence Identity [%]: 52 Evalue: 2.17e-41

>gi|6323703|ref|NP_013774.1| FET3 encodes a ferro-O2-oxidoreductase that is part of the high-affinity iron transport system; Fet3p [Saccharomyces cerevisiae]
MTNALLSIAVLLFSMLSLAQAETHTFNWTTGWDYRNVDGLKSRPVITCNGQFPWPDITVNKGDRVQIYLT
NGMNNTNTSMHFHGLFQNGTASMDGVPFLTQCPIAPGSTMLYNFTVDYNVGTYWYHSHTDGQYEDGMKGL
FIIKDDSFPYDYDEELSLSLSEWYHDLVTDLTKSFMSVYNPTGAEPIPQNLIVNNTMNLTWEVQPDTTYL
LRIVNVGGFVSQYFWIEDHEMTVVEIDGITTEKNVTDMLYITVAQRYTVLVHTKNDTDKNFAIMQKFDDT
MLDVIPSDLQLNATSYMVYNKTAALPTQNYVDSIDNFLDDFYLQPYEKEAIYGEPDHVITVDVVMDNLKN
GVNYAFFNNITYTAPKVPTLMTVLSSGDQANNSEIYGSNTHTFILEKDEIVEIVLNNQDTGTHPFHLHGH
AFQTIQRDRTYDDALGEVPHSFDPDNHPAFPEYPMRRDTLYVRPQSNFVIRFKADNPGVWFFHCHIEWHL
LQGLGLVLVEDPFGIQDAHSQQLSENHLEVCQSCSVATEGNAAANTLDLTDLTGENVQHAFIPTGFTKKG
IIAMTFSCFAGILGIITIAIYGMMDMEDATEKVIRDLHVDPEVLLNEVDENEERQVNEDRHSTEKHQFLT
KAKRFF

Modeled residue range: 22 to 550 based on template **1zpuC** (2.80 Å) Sequence Identity [%]: 100 Evalue: 0.00e-1

>gi|6323742|ref|NP_013813.1| SNZ1 proximal ORF, stationary phase induced gene; Sno1p [Saccharomyces cerevisiae]
MHKTHSTMSGKSMKVIGVLALQGAFLEHTNHLKRCLAENDYGIKIEIKTVKTPEDLAQCDALIIPGGEST
SMSLIAQRTGLYPCLYEFVHNPEKVVWGTCAGLIFLSAQLENESALVKTLGVLKVDVRRNAFGRQAQSFT
QKCDFSNFIPGCDNFPATFIRAPVIERILDPIAVKSLYELPVNGKDVVVAATQNHNILVTSFHPELADSD
TRFHDWFIRQFVSN

Modeled residue range: 15 to 218 based on template **2ywdA** (1.90 Å) Sequence Identity [%]: 38 Evalue: 1.22e-27

>gi|6323821|ref|NP_013892.1| Expression induced in response to heat shock, oxidative and osmotic stress. NAD+ is preferred coenzyme; Ald3p [Saccharomyces cerevisiae]
MPTLYTDIEIPQLKISLKQPLGLFINNEFCPSSDGKTIETVNPATGEPITSFQAANEKDVDKAVKAARAA
FDNVWSKTSSEQRGIYLSNLLKLIEEEQDTLAALETLDAGKPFHSNAKQDLAQIIELTRYYAGAVDKFNM
GETIPLTFNKFAYTLKVPFGVVAQIVPWNYPLAMACRKMQGALAAGNTVIIKPAENTSLSLLYFATLIKK
AGFPPGVVNVIPGYGSVVGKALGTHMDIDKISFTGSTKVGGSVLEASGQSNLKDITLECGGKSPALVFED
ADLDKAIEWVANGIFFNSGQICTANSRVYVQSSIYDKFVEKFKETAKKEWDVAGKFDPFDEKCIVGPVIS
STQYDRIKSYIERGKKEEKLDMFQTSEFPIGGAKGYFIPPTIFTDVPETSKLLRDEIFGPVVVVSKFTNY

DDALKLANDTCYGLASAVFTKDVKKAHMFARDIKAGTVWINQTNQEEAKVPFGGFKMSGIGRESGDTGVD
NYLQIKSVHVDLSLDK

Modeled residue range: 23 to 502 based on template **1o04G** (1.42 Å) Sequence Identity [%]: 47 Evalue: 1.28e-120

>gi|6323873|ref|NP_013944.1| GMP synthase; Gua1p [Saccharomyces cerevisiae]
MAAGEQVSNMFDTILVLDFGSQYSHLITRRLREFNIYAEMLPCTQKISELGWTPKGVILSGGPYSVYAED
APHVDHAIFDLNVPILGICYGMQELAWINGKQVGRGDKREYGPATLKVIDDSNSLFKGMNDSTVWMSHGD
KLHGLPTGYKTIATSDNSPYCGIVHETKPIYGIQFHPEVTHSTQGKTLLKNFAVDLCHAKQNWTMENFID
TEINRIRKLVGPTAEVIGAVSGGVDSTVASKLMTEAIGDRFHAILVDNGVLRLNEAANVKKTLVEGLGIN
LMVVDASEEFLSKLKGVTDPEKKRKIIGNTFIHVFEREAEKIKPKDGKEIQFLLQGTLYPDVIESISFKG
PSQTIKTHHNVGGLLENMKLKLIEPLRELFKDEVRHLGELLGIPHDLVWRHPFPGPGIAIRVLGEVTKEQ
VEIARKADNIYIEEIKKAGLYNQISQAFACLLPVKSVGVMGDQRTYDQVIALRAIETTDFMTADWFPFEH
SFLKKVASRIVNEVDGVARVTYDITSKPPATVEWE

Modeled residue range: 14 to 525 based on template **1gpmA** (2.20 Å) Sequence Identity [%]: 46 Evalue: 6.04e-142

>gi|6323995|ref|NP_014065.1| SNZ2 proximal ORF, stationary phase induced gene; Sno2p [Saccharomyces cerevisiae]
MTVVIGVLALQGAFIEHVRHVEKCIVENRDFYEKKLSVMTVKDKNQLAQCDALIIPGGESTAMSLIAERT
GFYDDLYAFVHNPSKVTWGTCAGMIYISQQLSNEEKLVKTLNLLKVKVKRNAFGRQAQSSTRICDFSNFI
PHCNDFPATFIRAPVIEEVLDPEHVQVLYKLDGKDNGGQELIVAAKQKNNILATSFHPELAENDIRFHDW
FIREFVLKNYSK

Modeled residue range: 4 to 212 based on template **2ywdA** (1.90 Å) Sequence Identity [%]: 35 Evalue: 8.95e-24

>gi|6324175|ref|NP_014245.1| membrane-bound casein kinase I homolog; Yck2p [Saccharomyces cerevisiae]
MSQVQSPLTATNSGLAVNNNTMNSQMPNRSNVRLVNGTLPPSLHVSSNLNHNTGNSSASYSGSQSRDDST
IVGLHYKIGKKIGEGSFGVLFEGTNMINGLPVAIKFEPRKTEAPQLKDEYRTYKILAGTPGIPQEYYFGQ
EGLHNILVIDLLGPSLEDLFDWCGRRFSVKTVVQVAVQMITLIEDLHAHDLIYRDIKPDNFLIGRPGQPD
ANKVHLIDFGMAKQYRDPKTKQHIPYREKKSLSGTARYMSINTHLGREQSRRDDMEAMGHVFFYFLRGQL
PWQGLKAPNNKQKYEKIGEKKRLTNVYDLAQGLPIQFGRYLEIVRNLSFEETPDYEGYRMLLLSVLDDLG
ETADGQYDWMKLNGGRGWDLSINKKPNLHGYGHPNPPNEKSKRHRSKNHQYSSPDHHHHYNQQQQQQAQ
AQAQAQAQAKVQQQQLQQAQAQQQANRYQLQPDDSHYDEEREASKLDPTSYEAYQQQTQQKYAQQQQKQM
QQKSKQFANTGANGQTNKYPYNAQPTANDEQNAKNAAQDRNSNKSSKGFFSKLGCC

Modeled residue range: 71 to 359 based on template **1csnA** (2.00 Å) Sequence Identity [%]: 67 Evalue: 5.13e-117

>gi|6324191|ref|NP_014261.1| 70-kDa adenylyl cyclase-associated protein; Srv2p [Saccharomyces cerevisiae]
MPDSKYTMQGYNLVKLLKRLEEATARLEDVTIYQEGYIQNKLEASKNNKPSDSGADANTTNEPSAENAPE
VEQDPKCITAFQSYIGENIDPLVELSGKIDTVVLDALQLLKGGFQSQLTFLRAAVRSRKPDYSSQTFADS
LRPINENIIKLGQLKESNRQSKYFAYLSALSEGAPLFSWVAVDTPVSMVTDFKDAAQFWTNRILKEYRES
DPNAVEWVKKFLASFDNLKAYIKEYHTTGVSWKKDGMDFADAMAQSTKNTGATSSPSPASATAAPAPPPP
PPAPPASVFEISNDTPATSSDANKGGIGAVFAELNQGENITKGLKKVDKSQQTHKNPELRQSSTVSSTGS
KSGPPPRPKKPSTLKTKRPPRKELVGNKWFIENYENETESLVIDANKDESIFIGKCSQVLVQIKGKVNAI
SLSETESCSVVLDSSISGMDVIKSNKFGIQVNHSLPQISIDKSDGGNIYLSKESLNTEIYTSCSTAINVN
LPIGEDDDYVEFPIPEQMKHSFADGKFKSAVFEHAG

Modeled residue range: 369 to 524 based on template **1k4zB** (2.30 Å) Sequence Identity [%]: 100 Evalue: 2.46e-82

>gi|6324268|ref|NP_014338.1| May participate in nucleolar function during the transition from stationary phase to rapid growth; Nop2p [Saccharomyces cerevisiae]
MGSRRHKNKQAAPPTLEEFQARKEKKANRKLEKGKRPSTTQGDEVSDRKKKKSKPFKKSRKEEEEVVEED
KDLPEVDLEELSKARKSLFDDEEDDDEAGLVDEELKDEFDLEQEYDYDEDEDNDAHPIFSDDDDEADLEE
LNAQNMEALSKKLDEEEAEEAEEAEMELVEAENMQPRADILPTEEQEEMMAQETPNLTSTRTRMIEIVKV
LENFKTLGAEGRSRGEYVDRLLKDICEYFGYTPFLAEKLFNLFSPAEAMEFFEANEIARPITIRTNTLKT
RRRDLAQTLVNRGVNLQPIGSWTKVGLQIFDSQVPIGATPEYLAGHYILQAASSFLPVIALDPHENERIL
DMAAAPGGKTTYISAMMKNTGCVFANDANKSRTKSLIANIHRLGCTNTIVCNYDAREFPKVIGGFDRILL
DAPCSGTGVIGKDQSVKVSRTEKDFIQIPHLQKQLLLSAIDSVDCNSKHGGVIVYSTCSVAVEEDEAVID
YALRKRPNVKLVDTGLAIGKEAFTSYRGKKFHPSVKLARRYYPHTYNVDGFFVAKFQKIGPSSFDDNQAS
AKEKETAARKEALEEGIIHSDFATFEDEEDDKYIEKSVKNNLLKKGVNPKAKRPSNEK

Modeled residue range: 228 to 549 based on template **1ixkA** (1.90 Å) Sequence Identity [%]: 36 Evalue: 2.87e-47

>gi|6324275|ref|NP_014345.1| Tyrosine protein phosphatase involved in adaptation response to pheromone; Msg5p [Saccharomyces cerevisiae]
MQFHSDKQHLDSKTDIDFKPNSPRSLQNRNTKNLSLDIAALHPLMEFSSPSQDVPGSVKFPSPTPLNLFM
KPKPIVLEKCPPKVSPRPTPPSLSMRRSEASIYTLPTSLKNRTVSPSVYTKSSTVSSISKLSSSSPLSSF
SEKPHLNRVHSLSVKTKDLKLKGIRGRSQTISGLETSTPISSTREGTLDSTDVNRFSNQKNMQTTLIFPE
EDSDLNIDMVHAEIYQRTVYLDGPLLVLPPNLYLYSEPKLEDILSFDLVINVAKEIPNLEFLIPPEMAHK
IKYYHIEWTHTSKIVKDLSRLTRIIHTAHSQGKKILVHCQCGVSRSASLIVAYIMRYYGLSLNDAYNKLK
GVAKDISPNMGLIFQLMEWGTMLSKNSPGEEGETVHMPEEDDIGNNEVSSTTKSYSSASFRSFPMVTNLS
SSPNDSSVNSSEVTPRTPATLTGARTALATERGEDDEHCKSLSQPADSLEASVDNESISTAPEQMMFLP

Modeled residue range: 229 to 374 based on template **2j16B** (2.70 Å) Sequence Identity [%]: 53 Evalue: 8.35e-39

>gi|6324374|ref|NP_014444.1| Protein required for cell viability; Ynr046wp [Saccharomyces cerevisiae]
MKFLTTNFLKCSVKACDTSNDNFPLQYDGSKCQLVQDESIEFNPEFLLNIVDRVDWPAVLTVAAELGNNA
LPPTKPSFPSSIQELTDDDMAILNDLHTLLLQTSIAEGEMKCRNCGHIYYIKNGIPNLLLPPHLV

Modeled residue range: 1 to 135 based on template **2j6aA** (1.70 Å) Sequence Identity [%]: 100 Evalue: 9.97e-73

>gi|6324648|ref|NP_014717.1| cell division cycle blocked at 36 degree C; Cdc21p [Saccharomyces cerevisiae]
MTMDGKNKEEEQYLDLCKRIIDEGEFRPDRTGTDANLLSEQGVKIWDGNGSREYLDKMGFKDRKVGDLGP
VYGFQWRHFGAKYKTCDDDYTGQGIDQLKQVIHKLKTNPYDRRIIMSAWNPADFDKMALPPCHIFSQFYV
SFPKEGEGSGKPRLSCLLYQRSCDMGLGVPFNIASYALLTRMIAKVVDMEPGEFIHTLGDAHVYKDHIDA
LKEQITRNPRPFPKLKIKRDVKDIDDFKLTDFEIEDYNPHPRIQMKMSV

Modeled residue range: 7 to 259 based on template **1f28A** (1.90 Å) Sequence Identity [%]: 54 Evalue: 1.01e-95

>gi|6324764|ref|NP_014833.1| Sporulation regulated genes; Spr1p [Saccharomyces cerevisiae]
MVSFRGLTTLTLLFTKLVNCNPVSTKNRDSIQFIYKEKDSIYSAINNQAINEKIHGVNLGGWLVLEPYIT
PSLFETFRTNPYNDDGIPVDEYHFCEKLGYEKAKERLYSHWSTFYKEEDFAKIASQGFNLVRIPIGYWAF
TTLSHDPYVTAEQEYFLDRAIDWARKYGLKVWIDLHGAAGSQNGFDNSGLRDSYKFLEDENLSATMKALT
YILSKYSTDVYLDTVIGIELLNEPLGPVIDMERLKNLLLKPAYDYLRNKINSNQIIVIHDAFQPYHYWDG
FLNDEKNEYGVIIDHHHYQVFSQVELTRKMNERIKIACQWGKDAVSEKHWSVAGEFSAALTDCTKWLNGV
GLGARYDGSWTKDNEKSHYINTCANNENIALWPEERKQNTRKFIEAQLDAFEMTGGWIMWCYKTENSIEW
DVEKLIQLNIFPQPINDRKYPNQCH

Modeled residue range: 42 to 444 based on template **1h4pA** (1.75 Å) Sequence Identity [%]: 67 Evalue: 1.57e-168

>gi|6324818|ref|NP_014887.1| contains amino-terminal chromodomains; Essential SAS family Acetyltransferase sharing homology with acetyltransferases from many different organisms; Esa1p [Saccharomyces cerevisiae]
MSHDGKEEPGIAKKINSVDDIIIKCQCWVQKNDEERLAEILSINTRKAPPKFYVHYVNYNKRLDEWITTD
RINLDKEVLYPKLKATDEDNKKQKKKKATNTSETPQDSLQDGVDGFSRENTDVMDLDNLNVQGIKDENIS
HEDEIKKLRTSGSMTQNPHEVARVRNLNRIIMGKYEIEPWYFSPYPIELTDEDFIYIDDFTLQYFGSKKQ
YERYRKKCTLRHPPGNEIYRDDYVSFFEIDGRKQRTWCRNLCLLSKLFLDHKTLYYDVDPFLFYCMTRRD
ELGHHLVGYFSKEKESADGYNVACILTLPQYQRMGYGKLLIEFSYELSKKENKVGSPEKPLSDLGLLSYR
AYWSDTLITLLVEHQKEITIDEISSMTSMTTTDILHTAKTLNILRYYKGQHIIFLNEDILDRYNRLKAKK
RRTIDPNRLIWKPPVFTASQLRFAW

Modeled residue range: 162 to 434 based on template **1fy7A** (2.00 Å) Sequence Identity [%]: 100 Evalue: 3.29e-158

>gi|6324899|ref|NP_014968.1| gamma-glutamyl phosphate reductase; Pro2p [Saccharomyces cerevisiae]
MSSSQQIAKNARKAGNILKTISNEGRSDILYKIHDALKANAHAIEEANKIDLAVAKETGLADSLLKRLDL
FKGDKFEVMLQGIKDVAELEDPVGKVKMARELDDGLTLYQVTAPVGVLLVIFESRPEVIANITALSIKSG
NAAILKGGKESVNTFREMAKIVNDTIAQFQSETGVPVGSVQLIETRQDVSDLLDQDEYIDLVVPRGSNAL
VRKIKDTTKIPVLGHADGICSIYLDEDADLIKAKRISLDAKTNYPAGCNAMETLLINPKFSKWWEVLENL
TLEGGVTIHATKDLKTAYFDKLNELGKLTEAIQCKTVDADEEQDFDKEFLSLDLAAKFVTSTESAIQHIN
THSSRHTDAIVTENKANAEKFMKGVDSSGVYWNASTRFADGFRYGFGAEVGISTSKIHARGPVGLDGLVS
YQYQIRGDGQVASDYLGAGGNKAFVHKDLDIKTVTL

Modeled residue range: 1 to 435 based on template **1vluA** (2.29 Å) Sequence Identity [%]: 91 Evalue: 0.00e-1

>gi|6324950|ref|NP_015019.1| Glucose repressed. Utilizes NADP+ or NAD+ as a coenzyme equally well.Ald4p [Saccharomyces cerevisiae]
MFSRSTLCLKTSASSIGRLQLRYFSHLPMTVPIKLPNGLEYEQPTGLFINNKFVPSKQNKTFEVINPSTE
EEICHIYEGREDDVEEAVQAADRAFSNGSWNGIDPIDRGKALYRLAELIEQDKDVIASIETLDNGKAISS
SRGDVDLVINYLKSSAGFADKIDGRMIDTGRTHFSYTKRQPLGVCGQIIPWNFPLLMWAWKIAPALVTGN
TVVLKTAESTPLSALYVSKYIPQAGIPPGVINIVSGFGKIVGEAITNHPKIKKVAFTGSTATGRHIYQSA
AAGLKKVTLELGGKSPNIVFADAELKKAVQNIILGIYYNSGEVCCAGSRVYVEESIYDKFIEEFKAASES
IKVGDPFDESTFQGAQTSQMQLNKILKYVDIGKNEGATLITGGERLGSKGYFIKPTVFGDVKEDMRIVKE
EIFGPVVTVTKFKSADEVINMANDSEYGLAAGIHTSNINTALKVADRVNAGTVWINTYNDFHHAVPFGGF
NASGLGREMSVDALQNYLQVKAVRAKLDE

Modeled residue range: 47 to 519 based on template **1o04G** (1.42 Å) Sequence Identity [%]: 51 Evalue: 2.21e-138

>gi|6325196|ref|NP_015264.1| Utilizes NADP+ as the preferred coenzyme. Activated by Mg2+; Ald6p [Saccharomyces cerevisiae]
MTKLHFDTAEPVKITLPNGLTYEQPTGLFINNKFMKAQDGKTYPVEDPSTENTVCEVSSATTEDVEYAIE
CADRAFHDTEWATQDPRERGRLLSKLADELESQIDLVSSIEALDNGKTLALARGDVTIAINCLRDAAAYA
DKVNGRTINTGDGYMNFTTLEPIGVCGQIIPWNFPIMMLAWKIAPALAMGNVCILKPAAVTPLNALYFAS
LCKKVGIPAGVVNIVPGPGRTVGAALTNDPRIRKLAFTGSTEVGKSVAVDSSESNLKKITLELGGKSAHL
VFDDANIKKTLPNLVNGIFKNAGQICSSGSRIYVQEGIYDELLAAFKAYLETEIKVGNPFDKANFQGAIT
NRQQFDTIMNYIDIGKKEGAKILTGGEKVGDKGYFIRPTVFYDVNEDMRIVKEEIFGPVVTVAKFKTLEE
GVEMANSSEFGLGSGIETESLSTGLKVAKMLKAGTVWINTYNDFDSRVPFGGVKQSGYGREMGEEVYHAY
TEVKAVRIKL

Modeled residue range: 28 to 500 based on template **1o04G** (1.42 Å) Sequence Identity [%]: 47 Evalue: 7.03e-125

>gi|6325237|ref|NP_015305.1| Ubl (ubiquitin-like protein) - specific protease 1; Ulp1p [Saccharomyces cerevisiae]
MSVEVDKHRNTLQYHKKNPYSPLFSPISTYRCYPRVLNNPSESRRSASFSGIYKKRTNTSRFNYLNDRRV
LSMEESMKDGSDRASKAGFIGGIRETLWNSGKYLWHTFVKNEPRNFDGSEVEASGNSDVESRSSGSRSSD
VPYGLRENYSSDTRKHKFDTSTWALPNKRRRIESEGVGTPSTSPISSLASQKSNCDSDNSITFSRDPFGW
NKWKTSAIGSNSENNTSDQKNSYDRRQYGTAFIRKKKVAKQNINNTKLVSRAQSEEVTYLRQIFNGEYKV
PKILKEERERQLKLMDMDKEKDTGLKKSIIDLTEKIKTILIENNKNRLQTRNENDDDLVFVKEKKISSLE
RKHKDYLNQKLKFDRSILEFEKDFKRYNEILNERKKIQEDLKKKKEQLAKKKLVPELNEKDDDQVQKALA
SRENTQLMNRDNIEITVRDFKTLAPRRWLNDTIIEFFMKYIEKSTPNTVAFNSFFYTNLSERGYQGVRRW
MKRKKTQIDKLDKIFTPINLNQSHWALGIIDLKKKTIGYVDSLSNGPNAMSFAILTDLQKYVMEESKHTI
GEDFDLIHLDCPQQPNGYDCGIYVCMNTLYGSADAPLDFDYKDAIRMRRFIAHLILTDALK

Modeled residue range: 403 to 621 based on template **1euvA** (1.60 Å) Sequence Identity [%]: 100 Evalue: 1.68e-124

>gi|6226540|ref|NP_009326.1| subunit II of cytochrome c oxidase; Cox2p [Saccharomyces cerevisiae]
MLDLLRLQLTTFIMNDVPTPYACYFQDSATPNQEGILELHDNIMFYLLVILGLVSWMLYTIVMTYSKNPI
AYKYIKHGQTIEVIWTIFPAVILLIIAFPSFILLYLCDEVISPAMTIKAIGYQWYWKYEYSDFINDSGET
VEFESYVIPDELLEEGQLRLLDTDTSMVVPVDTHIRFVVTAADVIHDFAIPSLGIKVDATPGRLNQVSAL
IQREGVFYGACSELCGTGHANMPIKIEAVSLPKFLEWLNEQ

Modeled residue range: 20 to 247 based on template **1v54O** (1.80 Å) Sequence Identity [%]: 42 Evalue: 5.21e-45

>gi|6319488|ref|NP_009570.1| Hypothetical ORF; Ybr014cp [Saccharomyces cerevisiae]
MAIVINKRNVRVLVITNLLLIVVFFVLRNSNASVNESITTHHPDSLVTFDNSGNAPGTHQSVHDTVNTQD
KEAEEVDKNSGDAEFDAAAEYNKIMEQSPMIVFSKTGCPYSKKLKALLTNSYTFSPSYYVVELDRHEHTK
ELQDQIEKVTGRRTVPNVIIGGTSRGGYTEIAELHKNDELLDSFKKWSDGAFTVKANSQSESA

Modeled residue range: 84 to 199 based on template **2e7pC** (2.10 Å) Sequence Identity [%]: 43 Evalue: 5.31e-15

>gi|6324975|ref|NP_015043.1| Hypothetical ORF; Ypl280wp [Saccharomyces cerevisiae]
MTPKRALISLTSYHGPFYKDGAKTGVFVVEILRSFDTFEKHGFEVDFVSETGGFGWDEHYLPKSFIGGED
KMNFETKNSAFNKALARIKTANEVNASDYKIFFASAGHGALFDYPKAKNLQDIASKIYANGGVIAAICHG
PLLFDGLIDIKTTRPLIEGKAITGFPLEGEIALGVDDILRSRKLTTVERVANKNGAKYLAPIHPWDDYSI
TDGKLVTGVNANSSYSTTIRAINALYS

Modeled residue range: 3 to 235 based on template **1rw7A** (1.80 Å) Sequence Identity [%]: 70 Evalue: 3.36e-90

>gi|6320193|ref|NP_010274.1| Hypothetical ORF; Ydl010wp [Saccharomyces cerevisiae]
MIPSNKRNARILSITTLLLLLVFFVAQNANFLTVEIKEETSKAFSTNMDNMAGGSSREYAAMPTSTTNKG
SSEVDEEINEIKQKVGLQQPIASVDDSLSAIKNDKGSRITKAFNVQKEYSLILDLSPIIIFSKSTCSYSK
GMKELLENEYQFIPNYYIIELDKHGHGEELQEYIKLVTGRGTVPNLLVNGVSRGGNEEIKKLHTQGKLLE
SLQVWSDGKFSVEQREKPSNN

Modeled residue range: 126 to 213 based on template **2e7pD** (2.10 Å) Sequence Identity [%]: 38 Evalue: 2.28e-11

>gi|6320661|ref|NP_010741.1| Hypothetical ORF; Ydr453cp [Saccharomyces cerevisiae]
MVAEVQKQAPPFKKTAVVDGIFEEISLEKYKGKYVVLAFVPLAFSFVCPTEIVAFSDAAKKFEDQGAQVL
FASTDSEYSLLAWTNLPRKDGGLGPVKVPLLADKNHSLSRDYGVLIEKEGIALRGLFIIDPKGIIRHITI
NDLSVGRNVNEALRLVEGFQWTDKNGTVLPCNWTPGAATIKPDVKDSKEYFKNANN

Modeled residue range: 3 to 195 based on template **1qmvH** (1.70 Å) Sequence Identity [%]: 62 Evalue: 1.33e-64

>gi|6320742|ref|NP_010822.1| Hypothetical ORF; Ydr533cp [Saccharomyces cerevisiae]
MAPKKVLLALTSYNDVFYSDGAKTGVFVVEALHPFNTFRKEGFEVDFVSETGKFGWDEHSLAKDFLNGQD
ETDFKNKDSDFNKTLAKIKTPKEVNADDYQIFFASAGHGTLFDYPKAKDLQDIASEIYANGGVVAAVCHG
PAIFDGLTDKKTGRPLIEGKSITGFTDVGETILGVDSILKAKNLATVEDVAKKYGAKYLAPVGPWDDYSI
TDGRLVTGVNPASAHSTAVRSIDALKN

Modeled residue range: 2 to 236 based on template **1rw7A** (1.80 Å) Sequence Identity [%]: 100 Evalue: 5.92e-126

>gi|14318475|ref|NP_116610.1| Hypothetical ORF; Yfl044cp [Saccharomyces cerevisiae]
MKLKVTGAGINQVVTLKQDATLNDLIEHINVDVKTMRFGYPPQRINLQGEDASLGQTQLDELGINSGEKI
TIESSDSNESFSLPPPQPKPKRVLKSTEMSIGGSGENVLSVHPVLDDNSCLFHAIAYGIFKQDSVRDLRE
MVSKEVLNNPVKFNDAILDKPNKDYAQWILKMESWGGAIEIGIISDALAVAIYVVDIDAVKIEKFNEDKF
DNYILILFNGIHYDSLTMNEFKTVFNKNQPESDDVLTAALQLASNLKQTGYSFNTHKAQIKCNTCQMTFV
GEREVARHAESTGHVDFGQNR

Modeled residue range: 91 to 301 based on template **3by4 (1.55** Å) Sequence Identity [%]: 100 Evalue: 1 e-124

>gi|14318532|ref|NP_116665.1| deubiquitinating enzyme (putative); Ubp6p [Saccharomyces cerevisiae]
MSGETFEFNIRHSGKVYPITLSTDATSADLKSKAEELTQVPSARQKYMVKGGLSGEESIKIYPLIKPGST
VMLLGTPDANLISKPAKKNNFIEDLAPEQQVQQFAQLPVGFKNMGNTCYLNATLQALYRVNDLRDMILNY
NPSQGVSNSGAQDEEIHKQIVIEMKRCFENLQNKSFKSVLPIVLLNTLRKCYPQFAERDSQGGFYKQQDA
EELFTQLFHSMSIVFGDKFSEDFRIQFKTTIKDTANDNDITVKENESDSKLQCHISGTTNFMRNGLLEGL
NEKIEKRSDLTGANSIYSVEKKISRLPKFLTVQYVRFFWKRSTNKKSKILRKVVFPFQLDVADMLTPEYA
AEKVKVRDELRKVEKEKNEKEREIKRRKFDPSSSENVMTPREQYETQVALNESEKDQWLEEYKKHFPPNL
EKGENPSCVYNLIGVITHQGANSESGHYQAFIRDELDENKWYKFNDDKVSVVEKEKIESLAGGGESDSAL
ILMYKGFGL

Modeled residue range: 103 to 499 based on template **1vjvA** (1.74 Å) Sequence Identity [%]: 92 Evalue: 0.00e-1

>gi|6323984|ref|NP_014055.1| Hypothetical ORF; Ymr322cp [Saccharomyces cerevisiae]
MTPKRALISLTSYHGPFYKDGAKTGVFVVEILRSFDTFEKHGFEVDFVSETGGFGWDEHYLPKSFIGGED
KMNFETKNSAFNKALARIKTANEVNASDYKIFFASAGHGALFDYPKAKNLQDIASKIYANGGVIAAICHG
PLLFDGLIDIKTTRPLIEGKAITGFPLEGEIALGVDDILRSRKLTTVERVANKNRAKYLAPIHPWDDYSI
TDGKLVTGVNANSSYSTTIRAINALYS

Modeled residue range: 3 to 235 based on template **1rw7A** (1.80 Å) Sequence Identity [%]: 70 Evalue: 6.61e-90

>gi|6323396|ref|NP_013468.1| Hypothetical ORF; Ylr364wp [Saccharomyces cerevisiae]
MSAFVTKAEEMIKSHPYFQLSASWCPDCVYANSIWNKLNVQDKVFVFDIGSLPRNEQEKWRIAFQKVVGS
RNLPTIVVNGKFWGTESQLHRFEAKGTLEEELTKIGLLP

modelled with HOMER.
residue range: 103 to 499 based on template **2ht9** (1.9 Å) Sequence Identity [%]: 30 pdb Blast Evalue: 2.0e-7

>gi|6323730|ref|NP_013801.1| Hypothetical ORF; Ymr084wp [Saccharomyces cerevisiae]
MCGIFGYCNFLIEKTRGEIIDTLIEGLQALEYKEYDSSGISIQGDELESLNIYKQTGKISSLKEEIDLYN
LNKNLPFISHCGIAHTRRATHGGLRRANCHPHNSDPSNEFVVVHNGVITNFANLKALLMAKGYVFKSDTD
TECIPKLYKHIYDTSIELGYNLDFHVLTNLVLKELEGSYGLLCTSSHFPDEVVAARKGSPLVIGVKGKTD
MDVNFVEVEYLDQEEDYLKLNTQTKSSGNVLAAAPVKYNTCLRKSPPFVHNT

Modeled residue range: 1 to 221 based on template **1xffA** (1.80 Å) Sequence Identity [%]: 37 Evalue: 1.39e-28

```
>gi|6323761|ref|NP_013832.1| Hypothetical ORF; Ymr114cp [Saccharomyces cerevisiae]
MCGRFALAYDSGDLPQLLRDWNLPVNTPKDASSNSQHPHDEEDTKDQPTVSKDIFKASYNISPTNYSAVY
RPDTKAIQFMRWGLVPFWTKDVSQFKTYRTFNARLENLQESKMWMRPCEKKRCAVLMSGYFEWKTVGKKK
TPYFISRRDGRLMFVAGMYDYVEKDDLYTFTIITAQGPRELEWLHERMPCVLEPGTESWDAWMDVDKTTW
STEELVKLLKPDYDESKLQFYQVTDDVGKTTNTGERLIKPLLKEDSDMFSVKREKEEALLENDNEQGIDN
RGVKGDKSLKGEDVFNQKKSLKRNSYDGLKKNEEQEETTLPEEGSIGDRVKREEANLSPKREGNREKRNI
VNMLGNQKDSRGKKKIKK
```

Modeled residue range: 77 to 253 based on template **2f20A** (2.10 Å) Sequence Identity [%]: 32 Evalue: 1.85e-17

```
>gi|6324967|ref|NP_015036.1| Hypothetical ORF; Yor391cp [Saccharomyces cerevisiae]
MTPKRALISLTSYHGPFYKDGAKTGVFVVEILRSFDTFEKHGFEVDFVSETGGFGWDEHYLPKSFIGGED
KMNFETKNSAFNKALARIKTANEVNASDYKVFFASAGHGALFDYPKAKNLQDIASKIYANGGVIAAICHG
PLLFDGLIDIKTTRPLIEGKAITGFPLEGEIALGVDDILRSRKLTTVERVANKNGAKYLAPIHPWDDYSI
TDGKLVTGVNANSSYSTTIRAINALYS
```

Modeled residue range: 3 to 235 based on template **1rw7A** (1.80 Å) Sequence Identity [%]: 70 Evalue: 3.36e-90

```
>gi|6319407|ref|NP_009489.1| Hypothetical ORF; Ybl064cp [Saccharomyces cerevisiae]
MFSRICSAQLKRTAWTLPKQAHLQSQTIKTFATAPILCKQFKQSDQPRLRINSDAPNFDADTTVGKINFY
DYLGDSWGVLFSHPADFTPVCTTEVSAFAKLKPEFDKRNVKLIGLSVEDVESHEKWIQDIKEIAKVKNVG
FPIIGDTFRNVAFLYDMVDAEGFKNINDGSLKTVRSVFVIDPKKKIRLIFTYPSTVGRNTSEVLRVIDAL
QLTDKEGVVTPINWQPADDVIIPPSVSNDEAKAKFGQFNEIKPYLRFTKSK
```

Modeled residue range: 55 to 258 based on template **1prxA** (2.00 Å) Sequence Identity [%]: 49 Evalue: 4.25e-47

```
>gi|6320492|ref|NP_010572.1| Hypothetical ORF; Ydr286cp [Saccharomyces cerevisiae]
MLRAFRCSIHTSRVLLHDAGVKLTFFSKPNCGLCDQAKEVIDDVFERKEFHNKAVSLEIVNITDRRNAKW
WKEYCFDIPVLHIEKVGDPKSCTKILHFLEEDDISDKIRRMQSR
```

Modeled residue range: 23 to 83 based on template **1wjkA** (99.9 Å) Sequence Identity [%]: 43 Evalue: 2.53e-7

```
>gi|6320714|ref|NP_010794.1| Hypothetical ORF; Ydr506cp [Saccharomyces cerevisiae]
MKVILLKPVQLPMLLLILLKFIMAAKEGKIHVLEFNASSEYTLDKRRVISINGYSATFGPEIRVKSGDTL
NLKLTNWICSEEEASKDSDVWKDYCSTALHFHGVVPLANEFDGIPGLTQPTIGYGESYWYNFTIDQSTCG
TFWYHSHSSVQYGDGMRGVLIVECDDYDNHVANTINSVRDIETLDDGVVTMKKDKHTKELTDYEVQERII
TLSDWYTNWNLDILNDKVLSSTGGTDPKFDGSLINGKSSDGETIKIGFNTEYLLLRIVNSGMSGTQVFHL
DGFQLIVLEADGIMIKPFIVQTINLAVGQRYTILVKLKSDTSFIRMINGCNKMMGYITKQWWFYKEGAHL
DLPKNPNDVSIEHLPGFTKAELYRDIEPTQEENKKLRTKADPVAVFEFDYAYYKDESTKQKYGTGMYKVN
ERTFSEYVKDPVRFGFNETYDIVINSLDHMRHPWHMHGHHFQIISLGNKGDGPFHKDVQEGKAWSRYQND
LRHLARTGKAPMVRDSINIAGNSYAVLRINTEMPGKWLLHCHVEWHMMKGLGIVFEVPTTTEDSTKQATT
AVLSYPTKEPDPNTVVHTAALEQNKSKVIAVYILIMCAVDAIFYWLLM

Modeled with HOMER
```

Modeled residue range: 25 to 546 based on template **1v10|A** (1.70 Å) Sequence Identity [%]: 24 Pdb Blast Evalue: 9.0e-30

```
>gi|6321388|ref|NP_011465.1| Hypothetical ORF; Ygl050wp [Saccharomyces cerevisiae]
MAAQNAFEQKKRAILNEIDSTQPDLSPKGTIDELCLPIIDLINASADMVTTSSCSGRVSVFLEGTKSYNG
```

EVKIGGKGQGGKWLYVTHDREKVIGWLDELKSKSEFSFELSGKEIPTEKVTGSIRYILYKYEPFILHVKC
RDFQAASKLYNTAMSCGFRESGIGSNNLVAIRINIKLDVPLGYLDETSGTLKFFVTPEYVSVLDSLSLSK
FDENTRKMQALYDRIEKELINCAPDVNSKVNITPIETKEERRERKKREGMERQRQLKSPQNVL

Modeled residue range: 28 to 220 based on template **2it2A** (1.50 Å) Sequence Identity [%]: 28 Evalue: 2.22e-9

>gi|6322078|ref|NP_012153.1| Hypothetical ORF; Yil113wp [Saccharomyces cerevisiae]
MNIYTSPTRTPNIAPKSGQRPSLPMLATDERSTDKESPNEDREFVPCSSLDVRRIYPKGPLLVLPEKIYL
YSEPTVKELLPFDVVINVAEEANDLRMQVPAVEYHHYRWEHDSQIALDLPSLTSIIHAATTKREKILIHC
QCGLSRSATLIIAYIMKYHNLSLRHSYDLLKSRADKINPSIGLIFQLMEWEVALNAKTNVQANSYRKVP

Modeled residue range: 54 to 196 based on template **2j16B** (2.70 Å) Sequence Identity [%]: 100 Evalue: 1.44e-76

>gi|6322467|ref|NP_012541.1| Hypothetical ORF; Yjr008wp [Saccharomyces cerevisiae]
MSIRPATHAGSWYSNRAQELSQQLHTYLIKSTLKGPIHNARIIICPHAGYRYCGPTMAYSYASLDLNRNV
KRIFILGPSHHIYFKNQILVSAFSELETPLGNLKVDTDLCKTLIQKEYPENGKKLFKPMDHDTDMAEHSL
EMQLPMLVETLKWREISLDTVKVFPMMVSHNSVDVDRCIGNILSEYIKDPNNLFIVSSDFCHWGRRFQYT
GYVGSKEELNDAIQEETEVEMLTARSKLSHHQVPIWQSIEIMDRYAMKTLSDTPNGERYDAWKQYLEITG
NTICGEKPISVILSALSKIRDAGPSGIKFQWPNYSQSSHVTSIDDSSVSYASGYVTIG

Modeled residue range: 4 to 337 based on template **3bczA** (2.10 Å) Sequence Identity [%]: 37 Evalue: 1.43e-49

>gi|6322557|ref|NP_012631.1| Hypothetical ORF; Yjr097wp [Saccharomyces cerevisiae]
MSLVNSLTHYEILRIPSDATQDEIKKAYRNRLLNTHPDKLSKSIHDTVSNVTINKIQDAYKILSNIKTRR
EYDRLILENYKRQGFHNCGDGLDEFSLDDFSFDEDKLEFMMNCPRCQFVGGFHFSESLLDECIDNVDAME
RSHSGYQLLTQCSACSLWLKVNFDIEEEQEGQ

Modeled with HOMER
Modeled residue range: 93 to 172 based on template 2ej7|A (99.9 Å) Sequence Identity [%]: 38
PDB Blast Evalue: 0.036

>gi|6322764|ref|NP_012837.1| Hypothetical ORF; Ykl086wp [Saccharomyces cerevisiae]
MSLQSNSVKPTEIPLSEIRRPLAPVLDPQKIDAMVATMKGIPTASKTCSLEQAEAAASAGELPPVDVLGV
RVKGQTLYYAFGGCHRLQAYDRRARETQNAAFPVRCRVLPATPRQIRMYLGSSLDIE

Modeled residue range: 1 to 123 based on template **1xw3A** (1.65 Å) Sequence Identity [%]: 33 Evalue: 3.16e-11

>gi|6323109|ref|NP_013181.1| Hypothetical ORF; Ylr080wp [Saccharomyces cerevisiae]
MTTRKTASSLQLLGKITGTKAGTKQKKMNFINGLIWLYMCVWMVHGKVTQKDELKWNKGYSLPNLLEVTD
QQKELSQWTLGDKVKLEEGRFVLTPGKNTKGSLWLKPEYSIKDAMTIEWTFRSFGFRGSTKGGLAFWLKQ
GNEGDSTELFGGSSKKFNGLMILLRLDDKLGESVTAYLNDGTKDLDIESSPYFASCLFQYQDSMVPSTLR
LTYNPLDNHLLKLQMDNRVCFQTRKVKFMGSSPFRIGTSAINDASKESFEILKMKLYDGVIEDSLIPNVN
PMGQPRVVTKVINSQTGEESFREKMPFSDKEESITSNELFEKMNKLEGKIMANDIDPLLRKMNKIVENER
ELIQRLRPLLDLKKTAISDDSFQDFLSMNANLDRLIKEQEKIRQDAKLYGKQTKGHDEIFSKISVWLALL
IFIMITLAYYMFRINQDIKKVKLL

Modeled residue range: 55 to 272 based on template **2a6vA** (1.52 Å) Sequence Identity [%]: 100 Evalue: 5.20e-122 (this is a crystal!!)

>gi|6323757|ref|NP_013828.1| Hypothetical ORF; Ymr110cp [Saccharomyces cerevisiae]
MSNDGSKILNYTPVSKIDEIVEISRNFFFEKQLKLSHENNPRKKDLEFRQLQLKKLYYAVKDHEEELIDA
MYKDFHRNKIESVLNETTKLMNDILHLIEILPKLIKPRRVSDSSPPFMFGKTIVEKISRGSVLIIAPFNF

PLLLAFAPLAAALAAGNTIVLKPSELTPHTAVVMENLLTTAGFPDGLIQVVQGAIDETTRLLDCGKFDLI
FYTGSPRVGSIVAEKAAKSLTPCVLELGGKSPTFITENFKASNIKIALKRIFFGAFGNSGQICVSPDYLL
VHKSIYPKVIKECESVLNEFYPSFDEQTDFTRMIHEPAYKKAVASINSTNGSKIVPSKISINSDTEDLCL
VPPTIVYNIGWDDPLMKQENFAPVLPIIEYEDLDETINKIIEEHDTPLVQYIFSDSQTEINRILTRLRSG
DCVVGDTVIHVGITDAPFGGIGTSGYGNYGGYYGFNTFSHERTIFKQPYWNDFTLFMRYPPNSAQKEKLV
RFAMERKPWFDRNGNNKWGLRQYFSLSAAVILISTIYAHCSS

Modeled residue range: 39 to 484 based on template **1ad3B** (2.60 Å) Sequence Identity [%]: 33 Evalue: 5.48e-66

>gi|6323962|ref|NP_014033.1| putative deubiquitinating enzyme; Ubp15p [Saccharomyces cerevisiae]
MSSEDELGSIGTVFFPGSPIDKSIGSILPQFDEEVETLLEDSFTWNIPDWNELTNPKYNSPRFRIGDFEWD
ILLFPQGNHNKGVAVYLEPHPEEKLDETTGEMVPVDPDWYCCAQFAIGISRPGNGDTINLINKSHHRFNA
LDTDWGFANLIDLNNLKHPSKGRPLSFLNEGTLNITAYVRILKDPTGVLWHNFLNYDSKKVTGYVGFRNQ
GATCYLNSLLQSYFFTKYFRKLVYEIPTEHESPNNSVPLALQRAFYQLQVSDIPLDTLELTRSFGWDTAE
SFTQHDVQELNRILMDRLENNMKGTPVEGKLNEIFVGKMKSYIKCINVDYESARVEDFWDLQLNVKNFKN
LQESFDNYIEMELMNGENQYAAQDYGLQDAQKGVIFESFPPVLHLQLKRFEYDFNYDQMVKVNDKYEFPE
TIDLSPFVDKDVLKKTLDSENKDKNPYVYNLHGVLVHSGDISTGHYYTLIKPGVEDQWYRFDDERVWRVT
KKQVFQENFGCDRLPDEKVRTMTRGEYQNYIIQRHTSAYMLVYIRQEQEEDLLRPVLESDVPKHVITRVR
EEIKERETKEKEIREAHLYVTLRLHSIKEFIHYEGFDYFAHDGFRLFAEELNDSGLQQINLKVLRTTKLS
DIFASIKETMNIPQERDVKYWKMDYRRNSTLRLTQPINFESVNITLQEALKKEKKRTMQTQYGEEGVAST
EEDDKALLETVSFLDLFIEEPYLELQFLNKLKEASLISKAQLDDELISTIRTNLPELTKGGIEPVFATDN
KSNLLFVKSYDPHTQKLLGFGHFAVNQLQQLSDISAIIEDSISSNEKLTFYEEVQPGTINEIYMKETIYD
ADIDTGDIVSFEVPGAVLPDTFPVYATIKDFYSYLRYRVKLKFSKFDGSSEEYGVSNEIPESFEFWISAY
APYDDLARMVSKYAHVKPEYLKIIALYSNGRFVLKSTSLLNDYLLKDFNCDQIPPFAFEVLSVPLKELER
LRPIKLYWLKNSYIHYQCFEFEVANDYTESQFLEKVQHKIGFTDEEKENILLWTNTNFQFQGLLSDQNTF
KDVSKHSLLFGRILPEESKLFKELNRLENVQTSSLEDFMDDENATDRPMDDEQDLGMAIEHSEDMKGRIV
VVQQYFKDLENRHGISFLFNLIPDETFPKTKDRLHAKFGLGQKEFSKIKLSIGYSTEEGTVFRSLQGFSD
EELDKVILYDIMSNLDYIYMDHPDRLRSHSSYDRPMIIKN

Modeled with HOMER
Modeled residue range: 39 to 575 based on template 2f1z (3.20 Å) Sequence Identity [%]: 40% PDBBlast Evalue: 2e-111

>gi|6324230|ref|NP_014300.1| Hypothetical ORF; Ynl099cp [Saccharomyces cerevisiae]
MTSKVGEYEDVPEDESRLTEENVSVPEEEVEDEDEEEDDDDDHIYINEETESGREKVLVSHAPQERIVPP
LNFCPVERYLYRSGQPSPVNFPFLLNLKLKTIIWLSNEEPQDTLLEFCDTHRINLQFAAINPDAGEDDNP
WDGLTEHSIINVLQTIVTQENYPLLVCCGMGRHRTGTVIGCLRRIMGWNLASVSEEYRRFTGSRGGRILV
ELLIEAFDTNLVKIDKNKAPSWLLTALE

Modeled residue range: 67 to 220 based on template **1xriA** (3.30 Å) Sequence Identity [%]: 35 Evalue: 2.92e-24

>gi|6324356|ref|NP_014426.1| Hypothetical ORF; Ynr029cp [Saccharomyces cerevisiae]
MSALRNIKFNEEEDGELPCLVTGEENNLQEILENVSYDGGNIVSDAKVERVNKQVENTSAGATDVHEKKR
IPVSIITGYLGSGKSTLLEKIALKGADKKIAVILNEFGDSSEIEKAMTIKNGSNSYQEWLDLGNGCLCCS
LKNIGVKAIEDMVERSPGKIDYILLETSGIADPAPIAKMFWQDEGLNSSVYIDGIITVLDCEHILKCLDD
ISIDAHWHGDKVGLEGNLTIAHFQLAMADRIIMNKYDTIEHSPEMVKQLKERVREINSIAPMFFTKYSDT
PIQNLLDIHAYDSVRISDILDSGSGNGTIHDDRMGTIMLTFRPLKNEEEYNNKFIKQFLQPLLWKNFGAM
TVLGGRRRDDGRDWEVQRTKGLILIEGENPIARVIQGVRDTYDVFPGKYDGSNKECKIVLIGKYLEKESI
EELLRKTLE

Modeled residue range: 71 to 291 based on template **1nijA** (2.00 Å) Sequence Identity [%]: 29 Evalue: 4.23e-20

>gi|6324401|ref|NP_014471.1| Hypothetical ORF; Ynr073cp [Saccharomyces cerevisiae]

MTKSDETTATSLNAKTLKSFESTLPIPTYPREGVKQGIVHLGVGAFHRSHLAVFMHRLMQEHHLKDWSIC
GVGLMKADALMRDAMKAQDCLYTLVERGIKDTNAYIVGSITAYMYAPDDPRAVIEKMANPDTHIVSLTVT
ENGYYHSEATNSLMTDAPEIINDLNHPEKPDTLYGYLYEALLLRYKRGLTPFTIMSCDNMPQNGVTVKTM
LVAFAKLKKDEKFAAWIEDKVTSPNSMVDRVTPRCTDKERKYVADTWGIKDQCPVVAEPFIQWVLEDNFS
DGRPPWELVGVQVVKDVDSYELMKLRLLNGGHSAMGYLGYLAGYTYIHEVVNDPTINKYIRVLMREEVIP
LLPKVPGVDFEEYTASVLERFSNPAIQDTVARICLMGSGKMPKYVLPSIYEQLRKPDGKYKLLAVCVAGW
FRYLTGVDMNGKPFEIEDPMAPTLKAAAVKGGKDPHELLNIEVLFSPEIRDNKEFVAQLTHSLETVYDKG
PIAAIKEILDQV

Modeled residue range: 12 to 501 based on template **1lj8A** (1.70 Å) Sequence Identity [%]: 42 Evalue: 1.72e-109

>gi|6324825|ref|NP_014894.1| Hypothetical ORF; Yor251cp [Saccharomyces cerevisiae]
MPLFDLISPKAFVKLVASEKVHRIVPVDATWYLPSWKLDNKVDFLTKPRIPNSIFFDIDAISDKKSPYPH
MFPTKKVFDDAMSNLGVQKDDILVVYDRVGNFSSPRCAWTLGVMGHPKVYLLNNFNQYREFKYPLDSSKV
AAFSPYPKSHYESSESFQDKEIVDYEEMFQLVKSGELAKKFNAFDARSLGRFEGTEPEPRSDIPSGHIPG
TQPLPYGSLLDPETKTYPEAGEAIHATLEKALKDFHCTLDPSKPTICSCGTGVSGVIIKTALELAGVPNV
RLYDGSWTEWVLKSGPEWIAENRD

Modeled with HOMER
Modeled residue range: 6 to 303 based on template **1ORB|A** (2.0 Å) Sequence Identity [%]: 31 Evalue: 4.00 e-34

>gi|6325169|ref|NP_015237.1| Hypothetical ORF; Ypl088wp [Saccharomyces cerevisiae]
MVLVKQVRLGNSGLKISPIVIGCMSYGSKKWADWVIEDKTQIFKIMKHCYDKGLRTFDTADFYSNGLSER
IIKEFLEYYSIKRETVVIMTKIYFPVDETLDLHHNFTLNEFEELDLSNQRGLSRKHIIAGVENSVKRLGT
YIDLLQIHRLDHETPMKEIMKALNDVVEAGHVRYIGASSMLATEFAELQFTADKYGWFQFISSQSYYNLL
YREDERELIPFAKRHNIGLLPWSPNARGMLTRPLNQSTDRIKSDPTFKSLHLDNLEEEQKEIINRVEKVS
KDKKVSMAMLSIAWVLHKGCHPIVGLNTTARVDEAIAALQVTLTEEEIKYLEEPYKPQRQRC

Modeled with Homer
Modeled residue range: 6 to 303 based on template **1PFY|A** (1.93 Å) Sequence Identity [%]: 29 PDB Blast Evalue: 3.0 e-27

>gi|6319721|ref|NP_009803.1| Glutathione peroxidase paralogue; Gpx2p [Saccharomyces cerevisiae]
MTTSFYDLECKDKKGESFKFDQLKGKVVLIVNVASKCGFTPQYKELEELYKKYQDKGFVILGFPCNQFGK
QEPGSDEQITEFCQLNYGVTFPIMKKIDVNGSNADSVYNYLKSQKAGLLGFKGIKWNFEKFLVDSNGKVV
QRFSSLTKPSSLDQEIQSLLSK

Modeled residue range: 4 to 160 based on template **2p5qD** (2.00 Å) Sequence Identity [%]: 51 Evalue: 6.10e-38

>gi|6319814|ref|NP_009895.1| Glutaredoxin; Grx1p [Saccharomyces cerevisiae]
MVSQETIKHVKDLIAENEIFVASKTYCPYCHAALNTLFEKLKVPRSKVLVLQLNDMKEGADIQAALYEIN
GQRTVPNIYINGKHIGGNDDLQELRETGELEELLEPILAN

Modeled residue range: 1 to 92 based on template **2jacA** (2.02 Å) Sequence Identity [%]: 99 Evalue: 4.15e-46

>gi|6320001|ref|NP_010081.1| 6-O-methylguanine-DNA methylase; Mgt1p [Saccharomyces cerevisiae]
MHKKKIENGRIFDLNGPTMKELLYYTFIETEVTGAFLVFREKTQNLVFASLGNDKLFLLGKVEGFLKKHE
KQDTMYDLQELKEAETYKKSIENYTICLENKMPLPSGAIPFEFLFGTDFQRKVWNELLNVEHGHVVTYGD
IAKRIGKPTAARSVGRACGSNNLALLVPCHRIVGSNRKLTGYKWSCKLKEQLLNNEKENSLSLSRL

Modeled residue range: 116 to 199 based on template **1sfeA** (2.10 Å) Sequence Identity [%]: 46 Evalue: 2.35e-19

>gi|6324862|ref|NP_014931.1| Disulfide isomerase related protein; Mpd1p [Saccharomyces cerevisiae]
MLFLNIIKLLLGLFIMNEVKAQNFYDSDPHISELTPKSFDKAIHNTNYTSLVEFYAPWCGHCKKLSSTFR
KAAKRLDGVVQVAAVNCDLNKNKALCAKYDVNGFPTLMVFRPPKIDLSKPIDNAKKSFSAHANEVYSGAR
TLAPIVDFSLSRIRSYVKKFVRIDTLGSLLRKSPKLSVVLFSKQDKISPVYKSIALDWLGKFDFYSISNK
KLKQLTDMNPTYEKTPEIFKYLQKVIPEQRQSDKSKLVVFDADKDKFWEYEGNSINKNDISKFLRDTFSI
TPNEGPFSRRSEYIAYLKTGKKPIKKNHSSSGNKHDEL

Modeled residue range: 24 to 155 based on template **2dmlA** (99.9 Å) Sequence Identity [%]: 42 Evalue: 1.81e-21

>gi|6319806|ref|NP_009887.1| Catalyzes the formation and isomerization of disulfide bonds durin
MKFSAGAVLSWSSLLLASSVFAQQEAVAPEDSAVVKLATDSFNEYIQSHDLVLAEFFAPWCGHCKNMAPEYVKAAETLVEKNITLAQIDCTENQDLC
MEHNIPGFPSLKIFKNSDVNNSIDYEGPRTAEAIVQFMIKQSQPAVAVVADLPAYLANETFVTPVIVQSGKIDADFNATFYSMANKHFNDYDFVSAE
NADDDFKLSIYLPSAMDEPVVYNGKKADIADADVFEKWLQVEALPYFGEIDGSVFAQYVESGLPLGYLFYNDEEELEEYKPLFTELAKKNRGLMNFV
SIDARKFGRHAGNLNMKEQFPLFAIHDMTEDLKYGLPQLSEEAFDELSDKIVLESKAIESLVKDFLKGDASPIVKSQEIFENQDSSVFQLVGKNHDE
IVNDPKKDVLVLYYAPWCGHCKRLAPTYQELADTYANATSDVLIAKLDHTENDVRGVVIEGYPTIVLYPGGKKSESVVYQGSRSLDSLFDFIKENGH
FDVDGKALYEEAQEKAAEEADADAELADEEDAIHDEL

Modeled residue range: 23 to 494 based on template 2b5eA (2.40 Å) Sequence Identity [%]: 100 Evalue: 0.00e-1)

>gi|6319816|ref|NP_009897.1| Hypothetical ORF; Ycl033cp [Saccharomyces cerevisiae] (Putative protein-methionine-R-oxide reductase)
MNKWSRLYVITVRRTFPGRRNIVLTQYWNKSKKMSDESNDVKWNDALTPLQLMVLRDKATERPNTGAYLHTNESGVYHCANCDRPLYSSKAKFDARC
GWPAFYEEVSPGAITYHRDNSLMPARVEICCARCGGHLGHVFEGEGWKQLLNLPKDTRHCVNSASLNLKKD

Modeled residue range: 39 to 166 based on template 3cezA (2.10 Å) Sequence Identity [%]: 39 Evalue: 1.49e-22

>gi|6320560|ref|NP_010640.1| Thioredoxin reductase; Trr1p [Saccharomyces cerevisiae]
MVHNKVTIIGSGPAAHTAAIYLARAEIKPILYEGMMANGIAAGGQLTTTTEIENFPGFPDGLTGSELMDRMREQSTKFGTEIITETVSKVDLSSKPF
KLWTEFNEDAEPVTTDAIILATGASAKRMHLPGEETYWQKGISACAVCDGAVPIFRNKPLAVIGGGDSACEEAQFLTKYGSKVFMLVRKDHLRASTI
MQKRAEKNEKIEILYNTVALEAKGDGKLLNALRIKNTKKNEETDLPVSGLFYAIGHTPATKIVAGQVDTDEAGYIKTVPGSSLTSVPGFFAAGDVQD
SKYRQAITSAGSGCMAALDAEKYLTSLE

Modeled with HOMER
Modeled residue range: 3 to 318 based on template 1VDC|A (2.50 Å) Sequence Identity [%]:62 PDB Blast Evalue: 6.0 e-111

>gi|6320598|ref|NP_010678.1| Protein with homology to mammalian ubiquitin activating (E1) enzym
MPRETSLVTIIGEDSYKKLRSSRCLLVGAGGIGSELLKDIILMEFGEIHIVDLDTIDLSNLNRQFLFRQKDIKQPKSTTAVKAVQHFNNSKLVPYQG
NVMDISTFPLHWFEQFDIIFNALDNLAARRYVNKISQFLSLPLIESGTAGFDGYMQPIIPGKTECFECTKKETPKTFPVCTIRSTPSQPIHCIVWAK
NFLFNQLFASETSGNEDDNNQDWGTDDAEEIKRIKQETNELYELQKIIISRDASRIPEILNKLFIQDINKLLAIENLWKTRTKPVPLSDSQINTPTK
TAQSASNSVGTIQEQISNFINITQKLMDRYPKEQNHIEFDKDDADTLEFVATAANIRSHIFNIPMKSVFDIKQIAGNIIPAIATTNAIVAGASSLIS
LRVLNLLKYAPTTKYTDLNMAFTAKASNLSQNRYLSNPKLAPPNKNCPVCSKVCRGVIKLSSDCLNKMKLSDFVVLIREKYSYPQDISLLDASNQRL
LFDYDFEDLNDRTLSEINLGNGSIILFSDEEGDTMIRKAIELFLDVDDELPCNTCSLPDVEVPLIKANNSPSKNEEEEKNEKGADVVATTNSHGKDG
IVILDDDEGEITIDAEPINGSKKRPVDTEISEAPSNKRTKLVNEPTNSDIVELD

Modeled residue range: 23 to 508 based on template 1y8qD (2.25 Å) Sequence Identity [%]: 38 Evalue: 4.02e-80

>gi|6320661|ref|NP_010741.1| thioredoxin peroxidase; Tsa2p [Saccharomyces cerevisiae]
MVAEVQKQAPPFKKTAVVDGIFEEISLEKYKGKYVVLAFVPLAFSFVCPTEIVAFSDAAKKFEDQGAQVLFASTDSEYSLLAWTNLPRKDGGLGPVK

VPLLADKNHSLSRDYGVLIEKEGIALRGLFIIDPKGIIRHITINDLSVGRNVNEALRLVEGFQWTDKNGTVLPCNWTPGAATIKPDVKDSKEYFKNA
NN

Modeled residue range: 3 to 195 based on template 1qmvH (1.70 Å) Sequence Identity [%]: 62 Evalue: 1.40e-64

>gi|6322186|ref|NP_012261.1| Eps1p [Saccharomyces cerevisiae]
R protein with chaperone and co-chaperone activity, involved in retention of resident ER proteins; has a role in recognizing proteins targeted for ER-associated degradation (ERAD), member of the protein disulfide isomerase family
MKMNLKRLVVTFFSCITFLLKFTIAAAEPPEGFPEPLNPTNFKEELSKGLHIIDFYSPYCPHCKHLAPVWMETWEEFKEESKTLNITFSQVNCIESA
DLCGDENIEYFPEIRLYNPSGYIKSFTETPRTKESLIAFARRESMDPNNLDTDLDSAKSESQYLEGFDFLELIAGKATRPHLVSFWPTKDMKNSDDS
LEFKNCDKCHEFQRTWKIISRQLAVDDINTGHVNCESNPTICEELGFGDLVKITNHRADREPKVALVLPNKTSNNLFDYPNGYSAKSDGYVDFARRT
FTNSKFPNITEGELEKKANRDIDFLQERGRVTNNDIHLVFSYDPETVVIEDFDILEYLIEPLSKIPNIYLHQIDKNLINLSRNLFGRMYEKINYDAS
QTQKVFNKEYFTMNTVTQLPTFFMFKDGDPISYVFPGYSTTEMRNIDAIMDWVKKYSNPLVTEVDSSNLKKLISFQTKSYSDLAIQLISSTDHKHIK
GSNKLIKNLLLASWEYEHIRMENNFEEINERRARKADGIKKIKEKKAPANKIVDKMREEIPHMDQKKLLLGYLDISKEKNFFRKYGITGEYKIGDVI
IIDKSNNYYYNKDNFGNSLTSNNPQLLREAFVSLNIPSKALYSSKLKGRLINSPFHNVLSFLDIIHGNGMPGYLIVIVLFIAILKGPSIYRRYKVRK
HYRAKRNAVGILGNMEKKKNQD

Modeled residue range: 26 to 151 based on template 2b5eA (2.40 Å) Sequence Identity [%]: 32 Evalue: 2.59e-12

>gi|6322228|ref|NP_012303.1| Hydroperoxide resistance conferring gene; Hyr1p [Saccharomyces cer
MSEFYKLAPVDKKGQPFPFDQLKGKVVLIVNVASKCGFTPQYKELEALYKRYKDEGFTIIGFPCNQFGHQEPGSDEEIAQFCQLNYGVTFPIMKKID
VNGGNEDPVYKFLKSQKSGMLGLRGIKWNFEKFLVDKKGKVYERYSSLTKPSSLSETIEELLKEVE

Modeled residue range: 11 to 160 based on template 2p5qA (2.00 Å) Sequence Identity [%]: 50 Evalue: 4.45e-36

>gi|14318501|ref|NP_116635.1| an FAD flavoprotein which contains a pair of redox-active cysteines
MLRIRSLLNNKRAFSSTVRTLTINKSHDVVIIGGGPAGYVAAIKAAQLGFNTACVEKRGKLGGTCLNVGCIPSKALLNNSHLFHQMHTEAQKRGIDV
NGDIKINVANFQKAKDDAVKQLTGGIELLFKKNKVTYYKGNGSFEDETKIRVTPVDGLEGTVKEDHILDVKNIIVATGSEVTPFPGIEIDEEKIVSS
TGALSLKEIPKRLTIIGGGIIGLEMGSVYSRLGSKVTVVEFQPQIGASMDGEVAKATQKFLKKQGLDFKLSTKVISAKRNDDKNVVEIVVEDTKTNK
QENLEAEVLLVAVGRRPYIAGLGAEKIGLEVDKRGRLVIDDQFNSKFPHIKVVGDVTFGPMLAHKAEEEGIAAVEMLKTGHGHVNYNNIPSVMYSHP
EVAWVGKTEEQLKEAGIDYKIGKFPFAANSRAKTNQDTEGFVKILIDSKTERILGAHIIGPNAGEMIAEAGLALEYGASAEDVARVCHAHPTLSEAF
KEANMAAYDKAIHC

Modeled residue range: 22 to 499 based on template 1v59B (2.20 Å) Sequence Identity [%]: 100 Evalue: 0.00e-1

>gi|6321898|ref|NP_011974.1| mitochondrial thioredoxin reductase; Trr2p [Saccharomyces cerevisiae]
MIKHIVSPFRTNFVGISKSVLSRMIHHKVTIIGSGPAAHTAAIYLARAEMKPTLYEGMMANGIAAGGQLTTTTDIENFPGFPESLSGSELMERMRKQ
SAKFGTNIITETVSKVDLSSKPFRLWTEFNEDAEPVTTDAIILATGASAKRMHLPGEETYWQQGISACAVCDGAVPIFRNKPLAVIGGGDSACEEAE
FLTKYASKVYILVRKDHFRASVIMQRRIEKNPNIIVLFNTVALEAKGDGKLLNMLRIKNTKSNVENDLEVNGLFYAIGHSPATDIVKGQVDEEETGY
IKTVPGSSLTSVPGFFAAGDVQDSRYRQAVTSAGSGCIAALDAERYLSAQE

Modeled residue range: 126 to 330 based on template 1tdeA (2.10 Å) Sequence Identity [%]: 51 Evalue: 4.03e-49

>gi|6321903|ref|NP_011979.1| Ubiquitin activating enzyme; Uba4p [Saccharomyces cerevisiae]
MNDYHLEDTTSELEALRLENAQLREQLAKREDSSRDYPLSLEEYQRYGRQMIVEETGGVAGQVKLKNTKVLVVGAGGLGCPALPYLAGAGVGQIGIV
DNDVVETSNLHRQVLHDSSRVGMLKCESARQYITKLNPHINVVTYPVRLNSSNAFDIFKGYNYILDCTDSPLTRYLVSDVAVNLGITVVSASGLGTE
GQLTILNFNNIGPCYRCFYPTPPPPNAVTSCQEGGVIGPCIGLVGTMMAVETLKLILGIYTNENFSPFLMLYSGFPQQSLRTFKMRGRQEKCLCCGK
NRTITKEAIEKGEINYELFCGARNYNVCEPDERISVDAFQRIYKDDEFLAKHIFLDVRPSHHYEISHFPEAVNIPIKNLRDMNGDLKKLQEKLPSVE
KDSNIVILCRYGNDSQLATRLLKDKFGFSNVRDVRGGYFKYIDDIDQTIPKY

Modeled residue range: 39 to 290 based on template 1jw9B (1.70 Å) Sequence Identity [%]: 35 Evalue: 1.05e-36

>gi|6322639|ref|NP_012712.1| similar to Uba2p; Uba1p [Saccharomyces cerevisiae] (Ubiquitin activiating enzyme E1)
MSSNNSGLSAAGEIDESLYSRQLYVLGKEAMLKMQTSNVLILGLKGLGVEIAKNVVLAGVKSMTVFDPEPVQLADLSTQFFLTEKDIGQKRGDVTRA
KLAELNAYVPVNVLDSLDDVTQLSQFQVVVATDTVSLEDKVKINEFCHSSGIRFISSETRGLFGNTFVDLGDEFTVLDPTGEEPRTGMVSDIEPDGT
VTMLDDNRHGLEDGNFVRFSEVEGLDKLNDGTLFKVEVLGPFAFRIGSVKEYGEYKKGGIFTEVKVPRKISFKSLKQQLSNPEFVFSDFAKFDRAAQ
LHLGFQALHQFAVRHNGELPRTMNDEDANELIKLVTDLSVQQPEVLGEGVDVNEDLIKELSYQARGDIPGVVAFFGGLVAQEVLKACSGKFTPLKQF
MYFDSLESLPDPKNFPRNEKTTQPVNSRYDNQIAVFGLDFQKKIANSKVFLVGSGAIGCEMLKNWALLGLGSGSDGYIVVTDNDSIEKSNLNRQFLF
RPKDVGKNKSEVAAEAVCAMNPDLKGKINAKIDKVGPETEEIFNDSFWESLDFVTNALDNVDARTYVDRRCVFYRKPLLESGTLGTKGNTQVIIPRL
TESYSSSRDPPEKSIPLCTLRSFPNKIDHTIAWAKSLFQGYFTDSAENVNMYLTQPNFVEQTLKQSGDVKGVLESISDSLSSKPHNFEDCIKWARLE
FEKKFNHDIKQLLFNFPKDAKTSNGEPFWSGAKRAPTPLEFDIYNNDHFHFVVAGASLRAYNYGIKSDDSNSKPNVDEYKSVIDHMIIPEFTPNANL
KIQVNDDDPDPNANAANGSDEIDQLVSSLPDPSTLAGFKLEPVDFEKDDDTNHHIEFITACSNCRAQNYFIETADRQKTKFIAGRIIPAIATTTSLV
TGLVNLELYKLIDNKTDIEQYKNGFVNLALPFFGFSEPIASPKGEYNNKKYDKIWDRFDIKGDIKLSDLIEHFEKDEGLEITMLSYGVSLLYASFFP
PKKLKERLNLPITQLVKLVTKKDIPAHVSTMILEICADDKEGEDVEVPFITIHL

C-term Modeled residue range: 597 to 861 based on template 1z7lC (2.80 Å) Sequence Identity [%]: 44 Evalue: 1.81e-62 C

>gi|6322826|ref|NP_012899.1| Glutathione peroxidase paralogue; Gpx1p [Saccharomyces cerevisiae]
MQEFYSFSPIDENGNPFPFNSLRNKVVLIVNVASHCAFTPQYKELEYLYEKYKSHGLVIVAFPCGQFGNQEFEKDKEINKFCQDKYGVTFPILHKIR
CNGQKQDPVYKFLKNSVSGKSGIKMIKWNFEKFVVDRNGKVVKRFSCMTRPLELCPIIEELLNQPPEEQI

Modeled residue range: 2 to 159 based on template 2p5qA (2.00 Å) Sequence Identity [%]: 43 Evalue: 1.46e-27

>gi|6323001|ref|NP_013073.1| Iron Sulfur Assembly -- IscA/NifA homolog; Isa1p [Saccharomyces ce
MINTGRSRNSVLLAHRFLSTGGFWRGGTNGTMSRTINNVNPFKLKFIPKTVPAAADSVSPDSQRPGKKPFKFIVSNQSKSSKASKSPKWSSYAFPSR
ETIKSHEEAIKKQNKAIDEQIAAAVSKNDCSCTEPPKKRKRKLRPRKALITLSPKAIKHLRALLAQPEPKLIRVSARNRGCSGLTYDLQYITEPGKF
DEVVEQDGVKIVIDSKALFSIIGSEMDWIDDKLASKFVFKNPNSKGTCGCGESFMV
(Cys178)

Modeled residue range: 147 to 239 based on template 1r94B (2.30 Å) Sequence Identity [%]: 38 Evalue: 4.70e-12

>gi|6323505|ref|NP_013576.1| essential, FAD-dependent oxidase of protein disulfide isomerase; E
(This is a Ero1-p)
MRLRTAIATLCLTAFTSATSNNSYIATDQTQNAFNDTHFCKVDRNDHVSPSCNVTFNELNAINENIRDDLSALLKSDFFKYFRLDLYKQCSFWDAND
GLCLNRACSVDVVEDWDTLPEYWQPEILGSFNNDTMKEADDSDDECKFLDQLCQTSKKPVDIEDTINYCDVNDFNGKNAVLIDLTANPERFTGYGGK
QAGQIWSTIYQDNCFTIGETGESLAKDAFYRLVSGFHASIGTHLSKEYLNTKTGKWEPNLDLFMARIGNFPDRVTNMYFNYAVVAKALWKIQPYLPE
FSFCDLVNKEIKNKMDNVISQLDTKIFNEDLVFANDLSLTLKDEFRSRFKNVTKIMDCVQCDRCRLWGKIQTTGYATALKILFEINDADEFTKQHIV
GKLTKYELIALLQTFGRLSESIESVNMFEKMYGKRLNGSENRLSSFFQNNFFNILKEAGKSIRYTIENINSTKEGKKKTNNSQSHVFDDLKMPKAEI
VPRPSNGTVNKWKKAWNTEVNNVLEAFRFIYRSYLDLPRNIWELSLMKVYKFWNKFIGVADYVSEETREPISYKLDIQ

Modeled residue range: 55 to 424 based on template 1rp4A (2.20 Å) Sequence Identity [%]: 100 Evalue: 0.00e-1

>gi|6324484|ref|NP_014553.1| protein disulfide isomerase related protein; Mpd2p [Saccharomyces
MKLHGFLFSVLSTCVVILPALAYSEAVTMVKSIEQYFDICNRNDSYTMIKYYTSWCQHCKTLAPVYEELGELYAKKANKDDTPINFLEVNCEFFGPT
LCTDLPGFPIIELVKPRTKPLVLPKLDWSSMKFHERLWQRIKTWFNNPKYQLDTSRVVRFEGSRNLKSLSNFIDTVRSKDTEERFIEHIFDDSRNCN
EELRSQQLLCKAGKEYYSDTLSKLYGDVNGLEKERRRLEALIKQNGDDLSKEVKEKLKIIRLQLSLLSHIEDQLEDTSSHDEL

Modeled residue range: 24 to 110 based on template 2djjA (99.9 Å) Sequence Identity [%]: 37 Evalue: 4.92e-9

```
>gi|6325166|ref|NP_015234.1| converts oxidized glutathione and NADPH into two glutathiones and N
(GR)
MLSATKQTFRSLQIRTMSTNTKHYDYLVIGGGSGGVASARRAASYGAKTLLVEAKALGGTCVNVGCVPKKVMWYASDLATRVSHANEYGLYQNLPLD
KEHLTFNWPEFKQKRDAYVHRLNGIYQKNLEKEKVDVVFGWARFNKDGNVEVQKRDNTTEVYSANHILVATGGKAIFPENIPGFELGTDSDGFFRLE
EQPKKVVVVGAGYIGIELAGVFHGLGSETHLVIRGETVLRKFDECIQNTITDHYVKEGINVHKLSKIVKVEKNVETDKLKIHMNDSKSIDDVDELIW
TIGRKSHLGMGSENVGIKLNSHDQIIADEYQNTNVPNIYSLGDVVGKVELTPVAIAAGRKLSNRLFGPEKFRNDKLDYENVPSVIFSHPEAGSIGIS
EKEAIEKYGKENIKVYNSKFTAMYYAMLSEKSPTRYKIVCAGPNEKVVGLHIVGDSSAEILQGFGVAIKMGATKADFDNCVAIHPTSAEELVTMR
```

Modeled residue range: 22 to 483 based on template 2hqmB (2.40 Å) Sequence Identity [%]: 100 Evalue: 0.00e-1

```
>gi|6325323|ref|NP_015391.1| Required for activation of RUB1 (ubiquitin-like protein) together
MDCKILVLGAGGLGCEILKNLTMLSFVKQVHIVDIDTIELTNLNRQFLFCDKDIGKPKAQVAAQYVNTRFPQLEVVAHVQDLTTLPPSFYKDFQFII
SGLDAIEPRRFINETLVKLTLESNYEICIPFIDGGTEGLKGHVKTIIPGITACWECSIDTLPSQQDTVPMCTIANNPRCIEHVVEYVSTIQYPDLNI
ESTADMEFLLEKCCERAAQFSISTEKLSTSFILGIIKSIIPSVSTTNAMVAATCCTQMVKIYNDLIDLENGNNFTLINCSEGCFMYSFKFERLPDCT
VCSNSNSN
```

Modeled residue range: 3 to 299 based on template 2nvuB (2.80 Å) Sequence Identity [%]: 43 Evalue: 5.36e-72
