# Supplementary material for: A Structure-Based Approach for Detection of Thiol Oxidoreductases and Their Catalytic Redox-Active Cysteine Residues
Source: PLoS Comput Biol. 2009 May 8;5(5):e1000383. doi: 10.1371/journal.pcbi.1000383 (PMC2673044; doi:10.1371/journal.pcbi.1000383)
Supplement: Table S5 — Detailed results for the yeast proteome. (0.04 MB PDF) [file pcbi.1000383.s011.pdf]

Table S5. Detailed results for the yeast proteome

ID	Best scoring Cys, position	Active Site Similarity score	Cys Reactivity score	Combined Score
>gi|6319726	75	0.5	1.75	**2.25**
>gi|6319966	81	0	0	**0**
>gi|6320720	60	3	1.75	**4.75**
>gi|6320796	26	0	1.5	**1.5**
>gi|6321022	36	2.5	1.25	**3.75**
>gi|14318471	212	0.5	2	**2.5**
>gi|14318558	122	1.25	2	**3.25**
>gi|6321631	149	1.25	2	**3.25**
>gi|6321706	202	0	1.5	**1.5**
>gi|6322180	106	3	0	**3**
>gi|6322468	149	0.5	1.5	**2**
>gi|6322559	89	0	1.25	**1.25**
>gi|6323613	47	2.75	1	**3.75**
>gi|6324090	101	0.5	0	**0.5**
>gi|6324639	103	0	1.5	**1.5**
>gi|6325168	38	0	0	**0**
>gi|6325296	120	1.5	2	**3.5**
>gi|6325330	13	2	1.5	**3.5**
>gi|6319925	57	3	1.5	**4.5**
>gi|6320304	193	0.5	0.25	**0.75**
>gi|6681846	129	1.5	2	**3.5**
>gi|6321648	33	3.5	2	**5.5**
>gi|6323072	32	3.5	1.5	**5**
>gi|6324082	62	0.5	0.5	**1**
>gi|6324296	213	0	2	**2**
>gi|6324360	17	0.5	0	**0.5**
>gi|6325255	102	0	0	**0**
>gi|6325339	38	0.25	0.25	**0.5**
>gi|6319498	157	1	1.5	**2.5**
>gi|6320303	71	2.5	1	**3.5**
>gi|6320695	55	1	1.5	**2.5**
>gi|6320917	321	0	2	**2**
>gi|6320972	776	0	0	**0**
>gi|6321025	193	0.75	0.25	**1**
>gi|14318479	284	0	1.25	**1.25**
>gi|14318481	126	0	0.25	**0.25**
>gi|6321691	251	0.25	2	**2.25**
>gi|6321832	643	0.75	0	**0.75**
>gi|6681848	178	0.75	1.25	**2**
>gi|6322155	62	1	2	**3**
>gi|6322811	195	0.25	0.5	**0.75**

>gi|6322921	79	1	2	**3**
>gi|6323224	171	1	1	**2**
>gi|6323383	168	1	1.5	**2.5**
>gi|6323609	179	0	0	**0**
>gi|6323703	483	0.25	0.5	**0.75**
>gi|6324191	405	0.75	1.25	**2**
>gi|6324275	318	0.5	2	**2.5**
>gi|6324374	111	1	1	**2**
>gi|6324648	131	0.75	1	**1.75**
>gi|6324818	217	0.25	1.5	**1.75**
>gi|6324899	257	0.75	1.5	**2.25**
>gi|6324950	323	0.25	2	**2.25**
>gi|6325237	579	0	0.75	**0.75**
>gi|6319488	107	2.5	0	**2.5**
>gi|6320193	135	3.5	0	**3.5**
>gi|6320661	47	3.5	0.25	**3.75**
>gi|6320742	137	0	1.5	**1.5**
>gi|14318475	274	0	1.5	**1.5**
>gi|14318532	117	0	0	**0**
>gi|6323396	24	2	2	**4**
>gi|6323761	122	0	2	**2**
>gi|6323984	137	0	0	**0**
>gi|6324975	138	0	0	**0**
>gi|6319407	90	2.5	0.25	**2.75**
>gi|6320492	33	3.25	1	**4.25**
>gi|6320714	530	0	1	**1**
>gi|6321388	53	1.75	1	**2.75**
>gi|6322078	139	0	2	**2**
>gi|6322467	283	1	0.5	**1.5**
>gi|6322557	154	1	1	**2**
>gi|6322764	83	0	0.75	**0.75**
>gi|6323109	229	0	0	**0**
>gi|6323757	272	0	2	**2**
>gi|6323962	324	0.5	0	**0.5**
>gi|6324230	167	0	1	**1**
>gi|6324356	135	0.25	2	**2.25**
>gi|6324401	196	0	1.25	**1.25**
>gi|6324825	258	0	0.75	**0.75**
>gi|6325169	22	0.5	0	**0.5**
>gi|6319733	53	0	0	**0**
>gi|6319971	251	0.5	2	**2.5**
>gi|6320389	114	0.25	0	**0.25**
>gi|6320610	434	0	0	**0**
>gi|6320726	61	3.5	0.25	**3.75**
>gi|6320881	24	3.5	2	**5.5**
>gi|6320917	321	0.25	2	**2.25**

<table>
<tr><td>>gi|14318459</td><td>90</td><td>0</td><td>0</td><td>0</td></tr>
<tr><td>>gi|14318551</td><td>282</td><td>0</td><td>0</td><td>0</td></tr>
<tr><td>>gi|6321643</td><td>874</td><td>1</td><td>1</td><td>2</td></tr>
<tr><td>>gi|6322027</td><td>168</td><td>1.75</td><td>0</td><td>1.75</td></tr>
<tr><td>>gi|6323138</td><td>61</td><td>2.5</td><td>0.25</td><td>2.75</td></tr>
<tr><td>>gi|6323742</td><td>99</td><td>0</td><td>0</td><td>0</td></tr>
<tr><td>>gi|6323821</td><td>301</td><td>0.5</td><td>2</td><td>2.5</td></tr>
<tr><td>>gi|6323873</td><td>88</td><td>0</td><td>0.5</td><td>0.5</td></tr>
<tr><td>>gi|6323995</td><td>90</td><td>0</td><td>0.75</td><td>0.75</td></tr>
<tr><td>>gi|6324268</td><td>477</td><td>1</td><td>2</td><td>3</td></tr>
<tr><td>>gi|6325196</td><td>305</td><td>0.25</td><td>2</td><td>2.25</td></tr>
<tr><td>>gi|6226540</td><td>220</td><td>0</td><td>1.5</td><td>1.5</td></tr>
<tr><td>>gi|6319806</td><td>406</td><td>3.5</td><td>0.75</td><td>4.25</td></tr>
<tr><td>>gi|6319816</td><td>157</td><td>2</td><td>2</td><td>4</td></tr>
<tr><td>>gi|6320560</td><td>142</td><td>2</td><td>1.5</td><td>3.5</td></tr>
<tr><td>>gi|6320598</td><td>176</td><td>1.5</td><td>0</td><td>1.5</td></tr>
<tr><td>>gi|6320661</td><td>48</td><td>3</td><td>0</td><td>3</td></tr>
<tr><td>>gi|6322186</td><td>60</td><td>3</td><td>1</td><td>4</td></tr>
<tr><td>>gi|6322228</td><td>36</td><td>3</td><td>0</td><td>3</td></tr>
<tr><td>>gi|14318501</td><td>65</td><td>1</td><td>2</td><td>3</td></tr>
<tr><td>>gi|6321898</td><td>165</td><td>1.25</td><td>2</td><td>3.25</td></tr>
<tr><td>>gi|6321903</td><td>225</td><td>0</td><td>1</td><td>1</td></tr>
<tr><td>>gi|6322639</td><td>600</td><td>1.5</td><td>0.25</td><td>1.75</td></tr>
<tr><td>>gi|6322826</td><td>36</td><td>2.5</td><td>2</td><td>4.5</td></tr>
<tr><td>>gi|6323001</td><td>178</td><td>0</td><td>0</td><td>0</td></tr>
<tr><td>>gi|6323505</td><td>100</td><td>3.25</td><td>2</td><td>5.25</td></tr>
<tr><td>>gi|6324484</td><td>56</td><td>3</td><td>1.25</td><td>4.25</td></tr>
<tr><td>>gi|6325166</td><td>61</td><td>1</td><td>2</td><td>3</td></tr>
<tr><td>>gi|6325323</td><td>168</td><td>0.75</td><td>1.25</td><td>2</td></tr>
<tr><td>>gi|6319814</td><td>26</td><td>3</td><td>1</td><td>4</td></tr>
<tr><td>>gi|6320001</td><td>168</td><td>0.75</td><td>2</td><td>2.75</td></tr>
<tr><td>>gi|6324862</td><td>61</td><td>3.5</td><td>1.25</td><td>4.75</td></tr>
<tr><td>>gi|6319721</td><td>36</td><td>3</td><td>0</td><td>3</td></tr>
<tr><td>>gi|6319522</td><td>57</td><td>0</td><td>0.25</td><td>0.25</td></tr>
<tr><td>>gi|6320210</td><td>16</td><td>0</td><td>0</td><td>0</td></tr>
<tr><td>>gi|6320228</td><td>57</td><td>1</td><td>1</td><td>2</td></tr>
<tr><td>>gi|6320998</td><td>77</td><td>1</td><td>0.5</td><td>1.5</td></tr>
<tr><td>>gi|14318550</td><td>34</td><td>0</td><td>0.5</td><td>0.5</td></tr>
<tr><td>>gi|7839183</td><td>47</td><td>0</td><td>1.25</td><td>1.25</td></tr>
<tr><td>>gi|6323103</td><td>53</td><td>0</td><td>0.5</td><td>0.5</td></tr>
<tr><td>>gi|6681849</td><td>76</td><td>0.25</td><td>2</td><td>2.25</td></tr>
<tr><td>>gi|6325198</td><td>59</td><td>0.5</td><td>0</td><td>0.5</td></tr>
<tr><td>>gi|6320259</td><td>94</td><td>0</td><td>2</td><td>2</td></tr>
<tr><td>>gi|6325229</td><td>90</td><td>0</td><td>1</td><td>1</td></tr>
<tr><td>>gi|6319741</td><td>118</td><td>1</td><td>1</td><td>2</td></tr>
</table>

>gi|6320303	71	3	0	**3**
>gi|6320808	159	1	0	**1**
>gi|6320869	222	0.5	0.75	**1.25**
>gi|6322409	149	1.5	2	**3.5**
>gi|14318463	211	1.5	0.5	**2**
>gi|14318480	204	0	1.75	**1.75**
>gi|6323822	301	0.5	0.25	**0.75**
>gi|6324764	433	1	0	**1**
>gi|6323730	1	0.25	1	**1.25**
>gi|6324967	137	0	1.5	**1.5**
>gi|6323244	143	0	0.25	**0.25**
>gi|6324070	17	0	2	**2**
>gi|6320936	736	0.25	1.5	**1.75**
>gi|6321228	103	1.25	1	**2.25**
>gi|6322908	175	0	0.25	**0.25**
>gi|6324175	156	0	0.5	**0.5**
