# Supplementary material for: A Structure-Based Approach for Detection of Thiol Oxidoreductases and Their Catalytic Redox-Active Cysteine Residues
Source: PLoS Comput Biol. 2009 May 8;5(5):e1000383. doi: 10.1371/journal.pcbi.1000383 (PMC2673044; doi:10.1371/journal.pcbi.1000383)
Supplement: Table S6 — Predicted yeast thiol oxidoreductases. (0.04 MB PDF) [file pcbi.1000383.s012.pdf]

Table S6. Predicted yeast thiol oxidoreductases

<table>
<tr><th></th><th>sequence id</th><th>template (%id homology modeling template)</th><th>Best Cys</th><th>Active Site Similarity Score</th><th>Cys Reactivity score</th><th>Combined score</th><th>Predicted by the Sec/Cys method</th><th>Protein (associated gene name)</th></tr>
<tr><td>1)</td><td>>gi|6320881</td><td>1fva (36%)</td><td>24</td><td>3.5</td><td>2</td><td>5.5</td><td>YES</td><td>MsrA (Mxr1)</td></tr>
<tr><td>2)</td><td>>gi|6321648</td><td>2fa4 (100%)</td><td>33</td><td>3.5</td><td>2</td><td>5.5</td><td>YES</td><td>Thioredoxin (Trx2)</td></tr>
<tr><td>3)</td><td>>gi|6323505</td><td>1rp4 (100%)</td><td>352</td><td>3.25</td><td>2</td><td>5.25</td><td>YES</td><td>Ero1-p (Ero1)</td></tr>
<tr><td>4)</td><td>>gi|6323072</td><td>2i9h (100%)</td><td>32</td><td>3.5</td><td>1.5</td><td>5</td><td>YES</td><td>Thioredoxin (Trx1)</td></tr>
<tr><td>5)</td><td>>gi|6320720</td><td>2jac (67%)</td><td>60</td><td>3</td><td>1.75</td><td>4.75</td><td>YES</td><td>Glutaredoxin (Grx2)</td></tr>
<tr><td>6)</td><td>>gi|6324862</td><td>2dml (42%)</td><td>61</td><td>3.5</td><td>1.25</td><td>4.75</td><td>YES</td><td>PDI related protein A (Mpd1)</td></tr>
<tr><td>7)</td><td>>gi|6322826</td><td>2p5q (43%)</td><td>36</td><td>2.5</td><td>2</td><td>4.5</td><td>YES</td><td>Glutathione peroxidase (Gpx1)</td></tr>
<tr><td>8)</td><td>>gi|6319925</td><td>2fa4 (43%)</td><td>57</td><td>3.5</td><td>1</td><td>4.5</td><td>YES</td><td>Thioredoxin (Trx3)</td></tr>
<tr><td>9)</td><td>>gi|6319806</td><td>2b5e (100%)</td><td>406</td><td>3.5</td><td>0.75</td><td>4.25</td><td>YES</td><td>PDI_A family (Pdi1)</td></tr>
<tr><td>10)</td><td>>gi|6320492</td><td>1wjk (43%)</td><td>33</td><td>3.25</td><td>1</td><td>4.25</td><td>YES</td><td>Unknown/glutaredoxin like protein (YDR286C)</td></tr>
<tr><td>11)</td><td>>gi|6319816</td><td>3cez (39%)</td><td>157</td><td>2</td><td>2</td><td>4</td><td>YES</td><td>SelR/MsrB (YCL033C)</td></tr>
<tr><td>12)</td><td>>gi|6324484</td><td>2djj (38%)</td><td>56</td><td>3</td><td>1.25</td><td>4.25</td><td>YES</td><td>Protein Disulfide Isomerase (Mpd2)</td></tr>
<tr><td>13)</td><td>>gi|6322186</td><td>2b5e (35%)</td><td>60</td><td>3</td><td>1</td><td>4</td><td>YES</td><td>Thioredoxin-like (Eps1)</td></tr>
<tr><td>14)</td><td>>gi|6323396</td><td>2ht9 (30%)</td><td>24</td><td>2</td><td>2</td><td>4</td><td>YES</td><td>Glutaredoxin (Grx8)</td></tr>
<tr><td>15)</td><td>>gi|6319814</td><td>2jac (98%)</td><td>26</td><td>3</td><td>1</td><td>4</td><td>YES</td><td>Glutaredoxin (Grx1)</td></tr>
<tr><td>16)</td><td>>gi|6323613</td><td>1qmv (66%)</td><td>47</td><td>2.75</td><td>1</td><td>3.75</td><td>YES</td><td>2cys peroxiredoxin (Tsa1)</td></tr>
<tr><td>17)</td><td>>gi|6320661</td><td>1qmv (60%)</td><td>48</td><td>3.5</td><td>0.25</td><td>3.75</td><td>YES</td><td>Peroxiredoxin (Tsa2)</td></tr>
<tr><td>18)</td><td>>gi|6320726</td><td>2b5e (42%)</td><td>61</td><td>3.5</td><td>0.25</td><td>3.75</td><td>YES</td><td>PDI-A family (Eug1)</td></tr>
<tr><td>19)</td><td>>gi|6320560</td><td>1vdc (62%)</td><td>142</td><td>2</td><td>1.5</td><td>3.5</td><td>YES</td><td>Thioredoxin reductase (Trr1)</td></tr>
<tr><td>20)</td><td>>gi|6325330</td><td>1dlq (99%)</td><td>13</td><td>2</td><td>1.5</td><td>3.5</td><td>NO</td><td>phosphotyrosine phosphatase (Ltp1)</td></tr>
<tr><td>21)</td><td>>gi|6320303</td><td>1wik (60%)</td><td>72</td><td>2.5</td><td>1</td><td>3.5</td><td>YES</td><td>Glutaredoxin (Grx3)</td></tr>
<tr><td>22)</td><td>>gi|6321022</td><td>2j23 (36%)</td><td>36</td><td>2.5</td><td>1</td><td>3.5</td><td>YES</td><td>Monothiol Glutaredoxin (Grx4)</td></tr>
<tr><td>23)</td><td>>gi|6681846</td><td>1oqc (47%)</td><td>129</td><td>1.5</td><td>2</td><td>3.5</td><td>YES</td><td>Erv-1 (Erv1)</td></tr>
<tr><td>24)</td><td>>gi|6320193</td><td>2e7p (38%)</td><td>135</td><td>3.5</td><td>0</td><td>3.5</td><td>YES</td><td>Glutaredoxin (Grx6)</td></tr>
<tr><td>25)</td><td>>gi|6325296</td><td>1jr8 (100%)</td><td>120</td><td>1.5</td><td>2</td><td>3.5</td><td>YES</td><td>Erv-2 (Erv2)</td></tr>
<tr><td>26)</td><td>>gi|6322409</td><td>2i5p (76%)</td><td>149</td><td>1.5</td><td>2</td><td>3.5</td><td>NO</td><td>Glyceraldehyde-3-phosphate dehydrogenase 1 (Tdh1)</td></tr>
<tr><td>27)</td><td>>gi|14318558</td><td>1ezv (100%)</td><td>122</td><td>1.25</td><td>2</td><td>3.25</td><td>NO</td><td>ubiquinol-cytochrome c oxidoreductase subunit 6 (Qcr6)</td></tr>
<tr><td>28)</td><td>>gi|6321898</td><td>1tde (51%)</td><td>165</td><td>1.25</td><td>2</td><td>3.25</td><td>YES</td><td>mitochondrial thioredoxin reductase (Trr2)</td></tr>
<tr><td>29)</td><td>>gi|6321631</td><td>2i5p (78%)</td><td>149</td><td>1.25</td><td>2</td><td>3.25</td><td>NO</td><td>Glyceraldehyde-3-phosphate dehydrogenase 3 (Tdh3)</td></tr>
<tr><td>30)</td><td>>gi|6325166</td><td>2hqm (100%)</td><td>61</td><td>1</td><td>2</td><td>3</td><td>YES</td><td>Glutathione reductase (Glr1)</td></tr>
<tr><td>31)</td><td>>gi|14318501</td><td>1v59 (100%)</td><td>65</td><td>1</td><td>2</td><td>3</td><td>YES</td><td>Dihydrolipoamide dehydrogenase (Lpd1)</td></tr>
<tr><td>32)</td><td>>gi|6322228</td><td>2p5q (50%)</td><td>36</td><td>3</td><td>0</td><td>3</td><td>YES</td><td>Glutathione peroxidase (Hyr1)</td></tr>
<tr><td>33)</td><td>>gi|6320661</td><td>1qmv (62%)</td><td>47</td><td>3</td><td>0</td><td>3</td><td>YES</td><td>Peroxiredoxin (TSA2)</td></tr>
<tr><td>34)</td><td>>gi|6322180</td><td>2a4v (98%)</td><td>106</td><td>3</td><td>0</td><td>3</td><td>YES</td><td>alkyl hydroperoxide reductase /AhpC (Dot5)</td></tr>
<tr><td>35)</td><td>>gi|6322155</td><td>1izn (50%)</td><td>62</td><td>1</td><td>2</td><td>3</td><td>NO</td><td>capping-addition of actin subunits (Cap2)</td></tr>
</table>

**36)**	>gi|6324268	1ixk (36%)	477	1	2	3	**NO**	nucleolar function in rapid growth (Nop2)
**37)**	>gi|6322921	1wc9 (58%)	79	1	2	3	**NO**	targeting and/or fusion of ER-to-Golgi transport vesicles (Bet3)
**38)**	>gi|6319721	2p5w (51%)	37	3	0	3	YES	Glutathione peroxidase paralogue (Gpx2p)
**39)**	>gi|6319407	1prx (49%)	90	2.5	0.25	2.75	YES	Mitochondrial peroxiredoxin (Prx1)
**40)**	>gi|6323138	1tp9 (40%)	61	2.5	0.25	2.75	YES	AhpD (Ahp1)
**41)**	>gi|6321388	2it2 (28%)	53	1.75	1	2.75	**NO**	uncharacterized protein (Tyw3)
**42)**	>gi|93117370	1sfe (48%)	168	0.75	2	2.75	YES	6-O-methylguanine-DNA methylase (Mgt1)
